# Synthesis and biological evaluation of a novel MUC1 glycopeptide conjugate vaccine candidate comprising a 4’-deoxy-4’-fluoro-Thomsen–Friedenreich epitope

**DOI:** 10.3762/bjoc.11.15

**Published:** 2015-01-23

**Authors:** Manuel Johannes, Maximilian Reindl, Bastian Gerlitzki, Edgar Schmitt, Anja Hoffmann-Röder

**Affiliations:** 1 Department of Chemistry and Center of Integrated Protein Science Munich (CIPSM), Ludwig-Maximilians University, Butenandtstraße 5–13, D-81377 Munich, Germany; 2 Institute for Immunology, University Medical Center of the Johannes Gutenberg-University Mainz, Langenbeckstraße 1, Geb. 708, D-55101 Mainz, Germany

**Keywords:** cancer immunotherapy, fluorinated carbohydrates, glycoconjugates, MUC1, TACA

## Abstract

The development of selective anticancer vaccines that provide enhanced protection against tumor recurrence and metastasis has been the subject of intense research in the scientific community. The tumor-associated glycoprotein MUC1 represents a well-established target for cancer immunotherapy and has been used for the construction of various synthetic vaccine candidates. However, many of these vaccine prototypes suffer from an inherent low immunogenicity and are susceptible to rapid in vivo degradation. To overcome these drawbacks, novel fluorinated MUC1 glycopeptide-BSA/TTox conjugate vaccines have been prepared. Immunization of mice with the 4’F-TF-MUC1-TTox conjugate resulted in strong immune responses overriding the natural tolerance against MUC1 and producing selective IgG antibodies that are cross-reactive with native MUC1 epitopes on MCF-7 human cancer cells.

## Introduction

Since cancer has advanced to one of the leading causes of death in economical developed countries, the search for novel anticancer therapies is of high current interest. For instance, promising approaches such as targeted therapy with small molecule tyrosine kinase inhibitors [1–2] and active cancer immune therapy have emerged [3–4] with the latter one being particularly attractive in terms of prevention and cure. Thus, administration of a therapeutic vaccine will trigger an effective immune response to allow eradication of tumor cells, which might have evaded surgery or have metastasized into the bloodstream [5]. A prerequisite for this approach is inter alia an efficient distinction of malignant cells from healthy ones by means of tumor-associated antigens (TACA), e.g., partial structures of the mucin glycoprotein MUC1 [6–7]. Cancer-associated MUC1 is characterized by the presence of specifically altered carbohydrate side chains in its extracellular tandem-repeat domain due to fundamental changes in glycosyltransferase activity and expression [8–10]. Furthermore, the prevalence of truncated saccharide antigens such as the Tn antigen [11], the Thomsen–Friedenreich (TF) antigen, and their sialylated congeners [8–10,12] leads to an insufficient shielding of the peptide backbone, which becomes accessible to the immune system and can be used as an additional immunogenic determinant for carbohydrate-based cancer vaccines [13].

Despite encouraging results with two- and three-component MUC1 conjugate vaccines in mice models [14–25], immune tolerance to carbohydrate antigens remains a major obstacle in initiating an effective and long-lasting immune protection against malignancies. One strategy to enhance the immunogenicity of carbohydrate antigens relies upon modification of the glycan hapten [26–27], which renders it more foreign to the host [28]. Earlier studies proved that non-natural TACAs can be indeed highly immunogenic [29–32], although the elicited antibodies often failed to cross-react with the natural glycan. To overcome this drawback, the use of synthetic carbohydrate-based vaccines with TACA analogs [33–34] in combination with cancer cell glycoengineering [35–38] was recently proposed, despite uncertainties associated with insufficient in vivo selectivity for tumors, which might hamper further application of this approach.

To allow for the use of vaccines derived from TACA analogs without metabolic cell glycoengineering, the structural integrity of the saccharide epitope must be maintained and cross-reactivity of the elicited antibodies with the native tumor-associated glycans is required. In this respect, the use of fluorinated TACAs [28,39–42] is a promising strategy due to the ability of C–F moieties to serve as effective C–OH mimics and the absence of fluorine in most organisms [43]. For instance, a MUC1 glycopeptide–tetanus toxoid conjugate (MUC1–TTox) containing a difluorinated 6,6’-F_2_-TF analog inducing a strong immune response in mice was recently reported [44]. More importantly, IgG antibodies elicited from this vaccine were found to cross-react with native TF epitopes of MCF-7 cancer cells. Similarly, Yang et al. found that fluorinated sTn antigen conjugates were significantly more immunogenic than their natural congeners and also elicited antibodies cross-reactive to sTn-positive LS-C tumor cells [32]. In this context and as part of ongoing research devoted to the development of selectively fluorinated MUC1 glycopeptide-based vaccines, we report herein on the successful preparation of two novel MUC1 glycoconjugates comprising 4’-deoxy-4’-fluoro-TF antigen side chains in their B cell epitopes. Use of tetanus toxoid (TTox) and bovine serum albumin (BSA) as immunogenic carrier proteins allows application of these conjugates in immunization studies and diagnostic ELISA experiments [44].

## Results and Discussion

### Chemical synthesis of the 4’-fluoro-TF neo-glycoconjugate

**Scheme 1:**
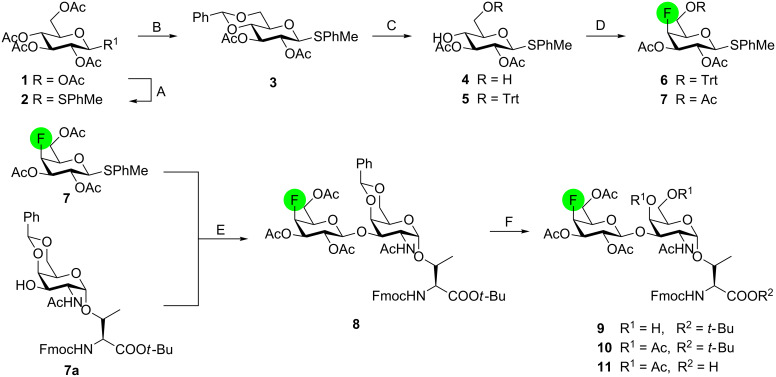
Reagents and conditions: (A) *p*-thiocresol, BF_3_∙Et_2_O, CH_2_Cl_2_, 24 h, rt, 81%; (B) i) NaOMe, MeOH, 12 h, rt; ii) benzaldehyde dimethylacetal, *p*-TsOH, DMF/MeCN 1:1, 18 h; 60 °C; iii) Ac_2_O, 4-DMAP, pyridine, 12 h, rt, 85% (3 steps); (C) i) aq 80% AcOH, 3 h, 90 °C; ii) TrtCl, 4-DMAP, pyridine, 24 h, 50 °C, 83% (2 steps); (D) i) Tf_2_O, pyridine, CH_2_Cl_2,_ 2 h, 0 °C; ii) TBAF, MS 4 Å, THF, 3 h, rt, 70% (2 steps); iii) aq 80% AcOH, 4 h, 90 °C; iv) Ac_2_O, pyridine, 4-DMAP, 12 h, rt, 78% (2 steps); (E) AgOTf, NIS, MS 4 Å, 24 h, 0 °C, 80%; (F) i) NaHSO_4_-SiO_2_, CH_2_Cl_2_/MeOH 4:1, rt, 18 h, 90%; ii) Ac_2_O, pyridine, 12 h, rt, 96%; iii) TFA, H_2_O, 2.5 h, rt, 98%.

The synthesis of the 4’-fluoro-TF SPPS building block **11** started by conversion of peracetylated D-glucose **1** into β-thio-glycoside **2** [45–46] under Lewis acid catalysis in 81% yield (Scheme 1). Subsequent Zemplén deacetylation [47], followed by 4,6-benzylidene acetal formation and acetylation provided fully protected precursor **3** [48] in excellent 85% yield over three steps. Acid-catalyzed 4,6-benzylidene deprotection and 6-*O*-tritylation then afforded alcohol **5** (83% over two steps), which was further converted into an intermediate triflate for subsequent nucleophilic fluorination with TBAF to provide the 4-fluorothioglycoside **6** in 70% yield. A two-step protecting group manipulation ultimately afforded glycosyl donor **7** with 78% yield.

The key step in the synthesis of the 4’-fluoro-TF-antigen glycosyl amino acid building block **10** is the stereoselective NIS/AgOTf-promoted coupling of **7** to the literature-known Tn antigen-threonine building block **7a** [49] affording the desired ß-configured 4’-deoxy-4’-fluorodisaccharide **8** in 80% yield. Subsequent two-step protecting group manipulation and acidolysis of the *tert*-butyl ester finally provided the orthogonally protected 4’-fluoro-TF-SPPS-building block **11** in 85% yield over three steps.

### Evaluation of in vitro stability against enzymatic degradation

**Figure 1:**
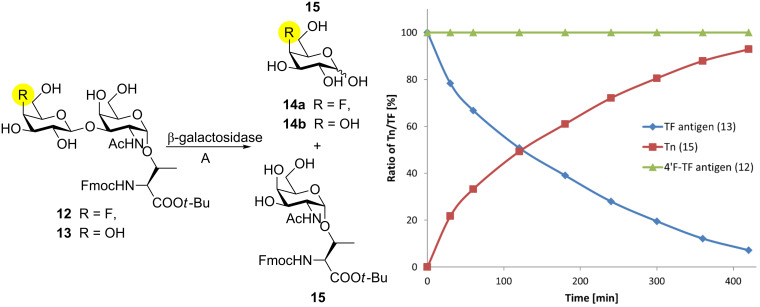
Degradation of 4’F-TF antigen derivative **12** and its natural (synthetic) congener **13** by β-galactosidase from bovine testes. The amounts of intact disaccharide and the evolving degradation product **15** were quantified by HPLC and expressed as a percentage of the corresponding value observed before exposure to enzyme digestion (*t* = 0); (A): β-cyclodextrin, MES buffer pH 4.5, 25 °C.

Besides improved immunogenicity, fluorinated TACAs may also feature an enhanced stability against enzymatic degradation, since many glycosidases do not accept fluorosugars, which can even act as mechanism-based enzyme inhibitors [50]. Based on the notion that the 4-fluoro substituent of **11** might also enhance the hapten’s bioavailability, which would be advantageous in long-term treatments, we decided to determine briefly its hydrolytic resistance by subjecting both the partially deprotected glycosyl amino acid **12** and its native counterpart **13** to enzymatic hydrolysis using a commercially available ß-galactosidase from bovine testes [E.C. 3.2.1.23] (Figure 1). Therefore, glycosyl amino acid **11** was carefully de-*O*-acetylated using NaOMe in MeOH at pH 8.5 followed by re-installment of the Fmoc protecting group. After HPLC purification, **12** was dissolved in an aqueous 2-(*N*-morpholine)ethanesulfonic acid (MES buffer) at pH 4.5 (enzyme activity optimum) and incubated with the enzyme in the presence of solubilizing 2,6-di-*O*-methyl-ß-cyclodextrin at 25 °C [51]. By virtue of analytical HPLC (UV detection based on Fmoc) and mass spectrometry, the amount of enzymatic digestion of the disaccharides **12** and **13**, which in both cases would yield the corresponding Tn monosaccharide **15** was determined over time (Figure 1 and Supporting Information File 1). Whereas half of the native TF derivative **13** was digested in approximately 3 h, the 4’F-TF antigen **12** remained intact over 7 h of incubation with the enzyme, suggesting that this compound is not a proper substrate and could presumably show an enhanced stability in vivo, as well.

### Synthesis of the fluorinated MUC1 glycopeptide–BSA/TTox conjugate vaccine candidate

**Scheme 2:**
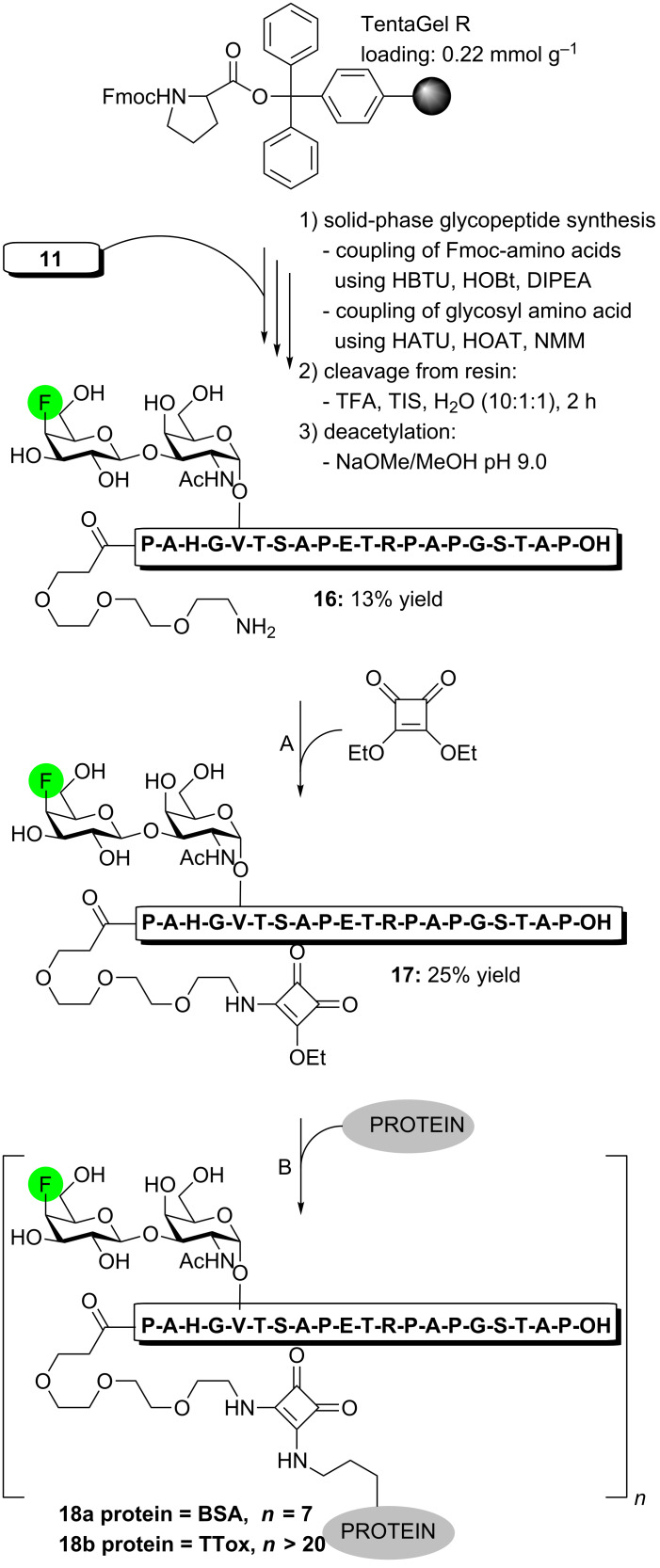
*Reagents and conditions:* (A) aq Na_2_CO_3_, pH 8.0, EtOH/H_2_O (1:1); (B) aq Na_2_HPO_4_, pH 9.5, 5 d.

Having demonstrated an improved enzymatic stability upon fluorination, glycosyl amino acid **11** was incorporated at position 6 of a full 20mer MUC1 *tandem repeat* domain by SPPS following a previously published procedure [44] (Scheme 2 and Supporting Information File 1). Thus, by using HBTU/HOBt/DIPEA in DMF for the coupling of the standard amino acids and the more reactive HATU/HOAt/NMM cocktail in NMP for attachment of building block **11** and a triethylene glycol spacer [52], the desired glycopeptide was assembled. Release from the resin using TFA/iPr_3_SiH/water (10:1:1) followed by careful de-*O*-acetylation with NaOMe/MeOH (pH 9.0) and preparative HPLC finally provided free glycopeptide **16** in 13% yield (over three steps). The latter was reacted with diethyl squarate in aqueous NaHCO_3_ solution (pH 8.0) [53] affording the corresponding squarate monoamide **17** (25% yield), which was further conjugated to bovine serum albumin (BSA) and tetanus toxoid (TTox) as carrier proteins in aqueous phosphate buffer at pH 9.5. Both 4’F-^6^TF-MUC1(20)-protein conjugates **18a/b** were obtained after ultrafiltration using a 30 kDa membrane.

MALDI–TOF mass spectrometry proved the antigen loading level of **18a** to be on average seven molecules of glycopeptide per molecule of BSA, whereas the corresponding antigen loading of the larger TTox conjugate **18b** was not likewise feasible. However, antigen loadings of >20 molecules of glycopeptide antigen per molecule of protein were estimated by ELISA binding data for similar MUC1 conjugates in earlier studies by the Kunz group [17].

### Immunological evaluation of the BSA/TTox conjugates

**Figure 2:**
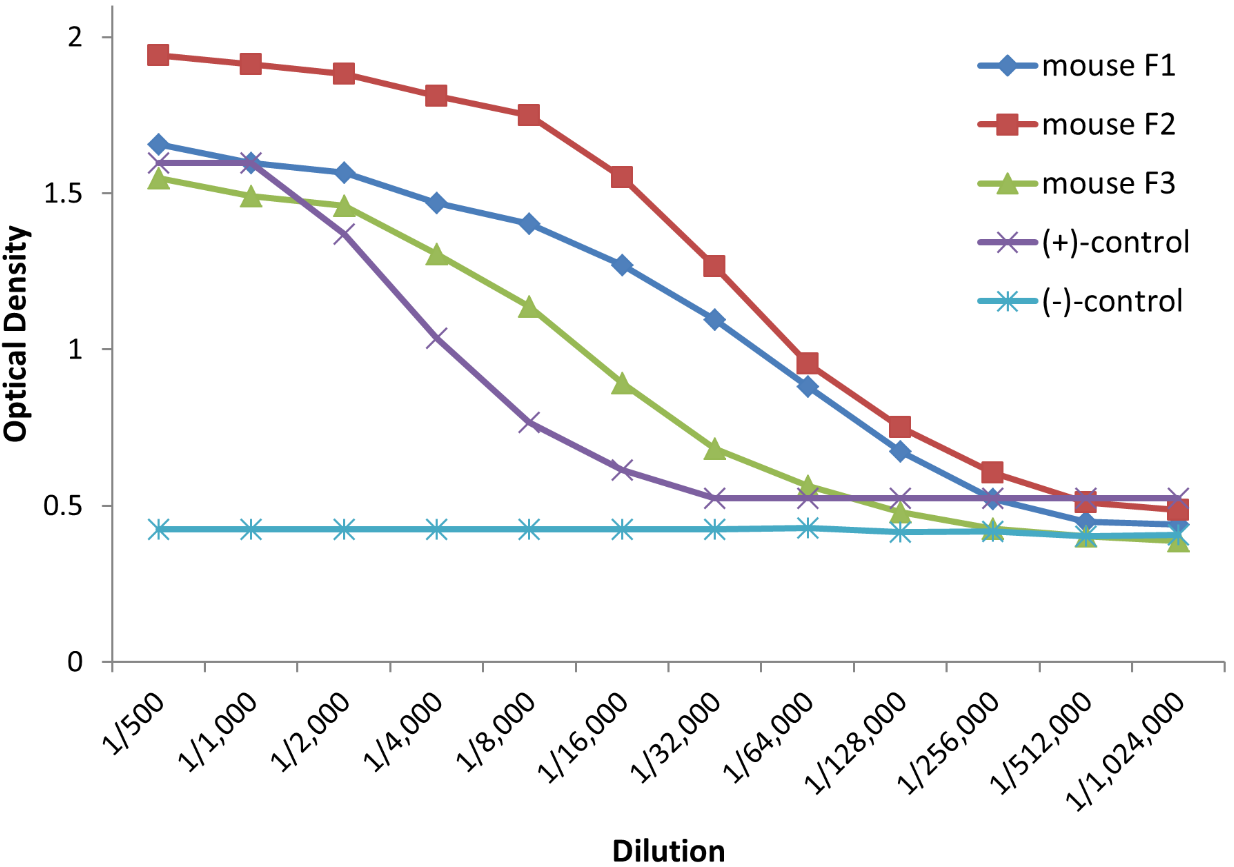
ELISA of the antiserum of mouse 2 induced by 4’F-TF-Thr^6^-MUC1(20)-TTox vaccine **18b**; coat: 5 µg/mL 4’F-TF-Thr^6^-MUC1(20)-BSA **18a** (for more details cf. Supporting Information File 1). Animals’ experiments were performed in accordance with institutional guidelines approved by Johannes Gutenberg-Universität Mainz and Landesuntersuchungsamt Koblenz.

In order to evaluate the immunological properties of the vaccine candidate **18b**, three female Balb/cj mice of 6–8 weeks were immunized subcutaneously with **18b** in the presence of complete Freund’s adjuvant (CFA). Two booster immunizations with incomplete Freund’s adjuvant (IFA) were performed by intraperitoneal applications at intervals of 21 days. Five days following the final immunization, serum antibody levels were determined by an enzyme-linked immunosorbent assay (ELISA). Therefore, blood was drawn from tail veins of the mice and the obtained sera were analysed using microtiter plates coated with the corresponding MUC1 glycopeptide–BSA conjugate **18a** (Figure 2 and Supporting Information File 1), in order to identify vaccine-induced antibodies [44]. The ELISA results of all three mice confirmed very strong immune responses capable of overcoming the natural tolerance (titers approximately 1/40000). Besides, strong immune responses against the carrier protein were determined for all mice sera (see Supporting Information File 1).

**Figure 3:**
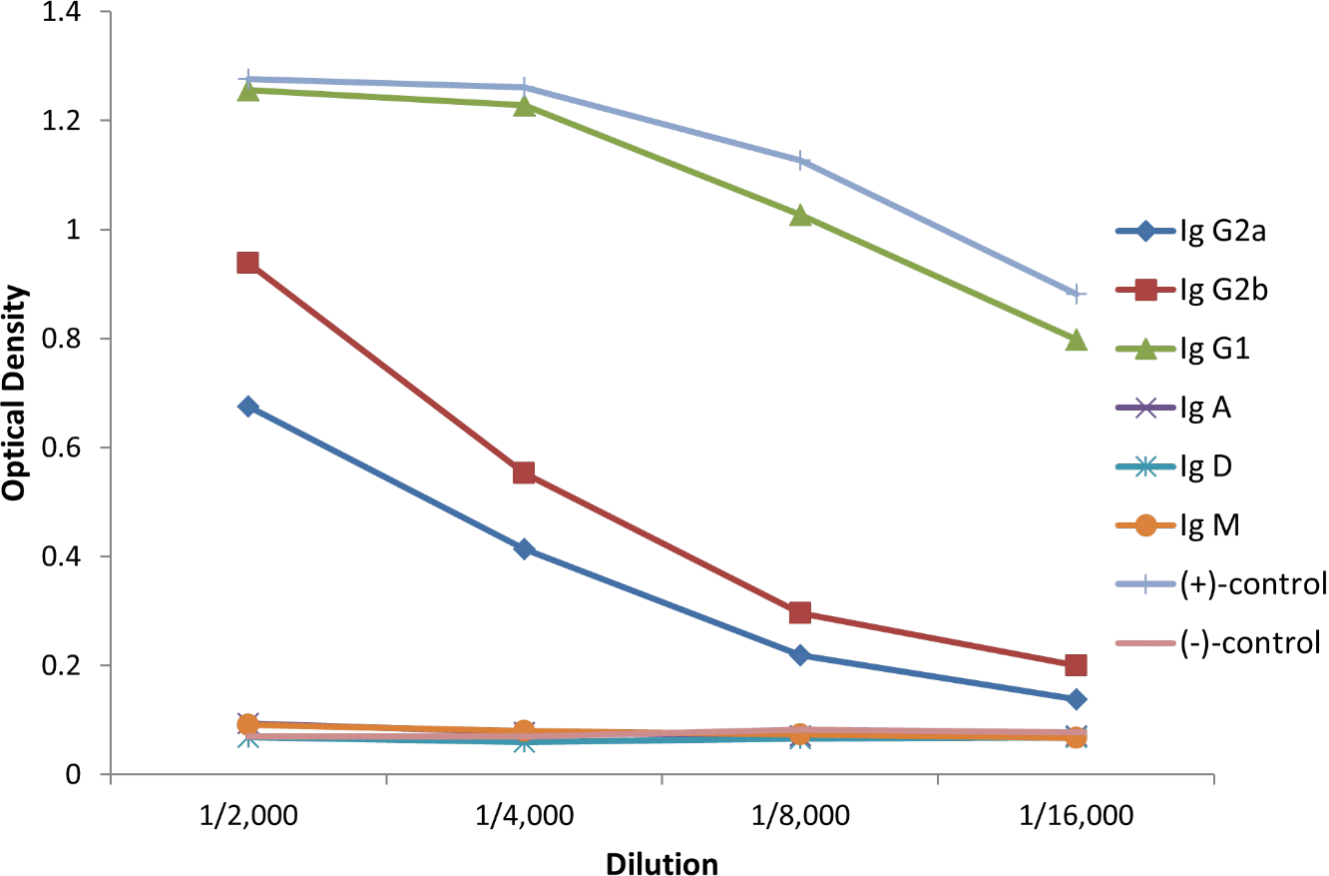
Determination of the isotypes of the antibodies induced by 4’F-TF-Thr^6^-MUC1(20)-TTox vaccine **18b** (antiserum of mice 2; for more details cf. Supporting Information File 1).

To further characterize the elicited immune responses, isotype analysis of the antisera using isotype-selective secondary antibodies was performed. ELISA experiments revealed the predominant induction of IgG1 antibodies and of a smaller IgG2a,b fraction following the third immunization (Figure 3). Moreover, with virtually no IgM antibody formation, an effective antibody class switching is assumed resulting in the desired MHCII restricted immune response, which is a crucial requirement for the establishment of an immunological memory.

**Figure 4:**
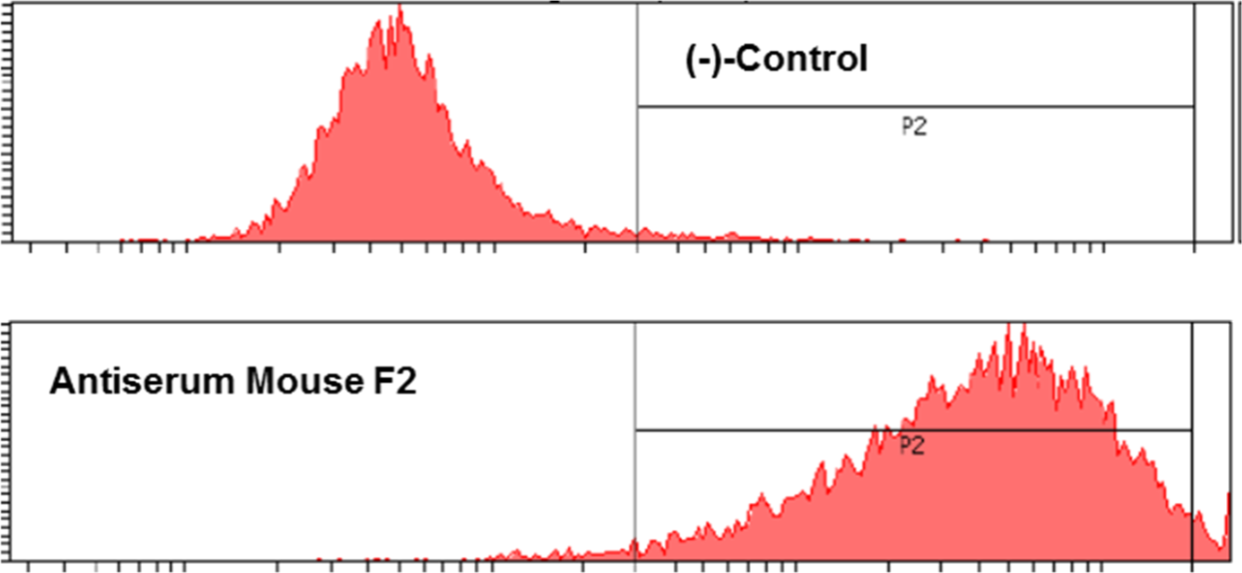
FACS analysis of the binding of MCF-7 tumor cells by the antiserum of mouse 2 induced by vaccination with **18b**: cells treated with buffer solution (top); MCF-7 cells treated with antiserum of mouse 2 (bottom); fluorescence intensity (y-axis) vs counts of cells (x-axis).

It is of major importance for the overall concept that the antisera obtained with vaccine **18b** are cross reactive to the native antigen structure exposed on the surface of tumor cells. Therefore, the binding of the induced antisera to MUC1-expressing MCF-7 human tumor cells was confirmed by flow cytometry using a fluorescently labelled goat anti-mouse-IgG antibody for visualization (see Supporting Information File 1). As shown in Figure 4, cells incubated with the 100-fold diluted antiserum of mouse 2 show fluorescence and appear all in the right area, whereas in the control experiment, cells treated with buffer solution do not show fluorescence and appear in the left area.

## Conclusion

In summary, we have synthesized novel MUC1 antitumor vaccine candidates comprising 4’-fluoro-^6^TF-antigen-MUC1 glycopeptides and BSA or TTox proteins as immunological carriers. The fluorinated MUC1 glycopeptide-TTox conjugate **18b** was successfully applied to immunizations of mice leading to strong tolerance-breaking immune responses and the production of selective IgG antibodies capable of binding to the native MUC1 antigen structures present on MCF-7 human breast cancer cells. Moreover, selective deoxyfluorination at the antigenic carbohydrate determinant might be used to improve the metabolic stability of the saccharide epitope, as was showcased by the enhanced resistance of the 4’-deoxy-4’-fluoro-TF-antigen-threonine conjugate **12** towards enzymatic degradation by ß-galactosidase from bovine testes. Thus, the work presented herein is an example of the successful application of strategically fluorinated glycopeptide derivatives as a tool to improve the immunogenicity and bioavailability of synthetic antigens and vaccines without affecting the induced antibody response and selectivity.

## Supporting Information

File 1 Experimental procedures for compounds **2**–**13**, **16**, **17**, **18a**, **18b**, **19**, **20**, protocols of biological evaluation and copies of NMR spectra of compounds **8**, **9**, **10**, **16**, **17**.
