# Supplementary material for: Synthesis and biological evaluation of a novel MUC1 glycopeptide conjugate vaccine candidate comprising a 4’-deoxy-4’-fluoro-Thomsen–Friedenreich epitope
Source: Beilstein J Org Chem. 2015 Jan 23;11:155–61. doi: 10.3762/bjoc.11.15 (PMC4311645; doi:10.3762/bjoc.11.15)
Supplement: File 1 — Experimental procedures for compounds 2–13, 16, 17, 18a, 18b, 19, 20, protocols of biological evaluation and copies of NMR spectra of compounds 8, 9, 10, 16, 17. [file Beilstein_J_Org_Chem-11-155-s001.pdf]

Supporting Information

for

Synthesis and biological evaluation of a novel MUC1 glycopeptide conjugate vaccine candidate comprising a 4'-deoxy-4'-fluoro-Thomsen–Friedenreich epitope

Manuel Johannes[§1], Maximilian Reindl[§1], Bastian Gerlitzki[2], Edgar Schmitt[2] and Aanja Hoffmann-Röder*[1]

Address: [1]Department of Chemistry and Center of Integrated Protein Science Munich (CIPS[M]), Ludwig-Maximilians University, Butenandtstraße 5–13, D-81377 Munich, Germany and [2]Institute for Immunology, University Medical Center of the Johannes Gutenberg-University Mainz, Langenbeckstraße 1, Geb. 708, D-55101 Mainz, Germany

Email: Anja Hoffmann-Röder - anja.hoffmann-roeder@cup.uni-muenchen.de

*Corresponding author

[§]Both authors contributed equally to this work.

Experimental procedures for compounds 2–13, 16, 17, 18a, 18b, 19, 20, protocols of biological evaluation and copies of NMR spectra of compounds 8, 9, 10, 16, 17

Table of Contents

1 General Remarks S3

2 Experimental Procedures S4

2.1 4-Methylphenyl 2,3,4,6-tetra-*O*-acetyl-1-thio-β-D-glucopyranoside (**2**) S4

2.2 4-Methylphenyl 2,3-di-*O*-acetyl-4,6-*O*-benzylidene-1-thio-β-D-glucopyranoside (**3**) .. S5

2.3 4-Methylphenyl 2,3-di-*O*-acetyl-1-thio-β-D-glucopyranoside (**4**) S6

2.4 4-Methylphenyl 2,3-di-*O*-acetyl-6-*O*-trityl-1-thio-β-D-glucopyranoside (**5**) S6

2.5 4-Methylphenyl 2,3-di-*O*-acetyl-4-deoxy-4-fluoro-6-*O*-trityl-1-thio-β-D-glucopyranoside (**6**) S7

2.6 4-Methylphenyl 2,3,6-tri-*O*-acetyl-4-deoxy-4-fluoro-1-thio-β-D-glucopyranoside (**7**) . S8

2.7 *N*-(9*H*-Fluoren-9-yl)-methoxycarbonyl-*O*-(2-acetamido-2-deoxy-4,6-*O*-benzylidene-3-*O*-[2,3,6-tri-*O*-acetyl-β-D-4-deoxy-4-fluoro-galactopyranosyl]-α-D-galactopyranosyl)-L-threonine *tert*-butyl ester (**8**) S10

2.8 *N*-(9*H*-Fluoren-9-yl)-methoxycarbonyl-*O*-(2-acetamido-2-deoxy-3-*O*-[2,3,6-tri-*O*-acetyl-β-D-4-deoxy-4-fluoro-galactopyranosyl]-α-D-galactopyranosyl)-L-threonine *tert*-butyl ester (**9**) S11

2.9 *N*-(9*H*-Fluoren-9-yl)-methoxycarbonyl-*O*-(4,6-di-*O*-acetyl-2-acetamido-2-deoxy-3-*O*-[2,3,6-tri-*O*-acetyl-β-D-4-deoxy-4-fluoro-galactopyranosyl]-α-D-galactopyranosyl)-L-threonine *tert*-butyl ester (**10**) S13

2.10 *N*-(9*H*-Fluoren-9-yl)-methoxycarbonyl-*O*-(4,6-di-*O*-acetyl-2-acetamido-2-deoxy-3-*O*-[2,3,6-tri-*O*-acetyl-β-D-4-deoxy-4-fluoro-galactopyranosyl]-α-D-galactopyranosyl)-L-threonine (**11**) S14

2.11 Amino-4,7,10-trioxadodecanylamido-*N*-L-prolyl-L-Alanyl-L-histidyl-L-glycyl-L-valyl-*O*-(2-acetamido-2-deoxy-3-*O*-[4-deoxy-4-fluoro-β-D-galactopyranosyl]-α-D-galactopyranosyl)-L-threonyl-L-seryl-L-alanyl-L-prolyl-L-aspartyl-L-threonyl-L-arginyl-L-porlyl-L-alanyl-L-prolyl-L-glycyl-L-seryl-L-threonyl-L-alanyl-L-proline (**16**) S15

2.12 1-(Amino-4,7,10-trioxadodecanylamido-*N*-L-prolyl-L-Alanyl-L-histidyl-L-glycyl-L-valyl-*O*-(2-acetamido-2-deoxy-3-*O*-[4-deoxy-4-fluoro-β-D-galactopyranosyl]-α-D-galactopyranosyl)-L-threonyl-L-seryl-L-alanyl-L-prolyl-L-aspartyl-L-threonyl-L-arginyl-L-porlyl-L-alanyl-L-prolyl-L-glycyl-L-seryl-L-threonyl-L-alanyl-L-prolyl)-2-ethoxycyclobutene-3,4-dione (**17**) S17

2.13 BSA-conjugate (**18a**) S18

2.14 Tetanus-Toxoid-conjugate (**18b**) S19

3 Enzymatic degradation S20

3.1 *N*-(9*H*-Fluoren-9-yl)-methoxycarbonyl-*O*-(2-acetamido-2-deoxy-4,6-*O*-benzylidene-3-*O*-[2,3,4,6-tetra-*O*-acetyl-β-D-pyranosyl]-α-D-galactopyranosyl)-L-threonine *tert*-butyl ester (**19**) S20

3.2 *N*-(9*H*-Fluoren-9-yl)-methoxycarbonyl-*O*-(2-acetamido-2-deoxy-3-*O*-[2,3,4,6-tetra-*O*-acetyl-β-D-pyranosyl]-α-D-galactopyranosyl)-L-threonine *tert*-butyl ester (**20**) S21

3.3 *N*-(9*H*-Fluoren-9-yl)-methoxycarbonyl-*O*-(2-acetamido-2-deoxy-3-*O*-[β-D-galactopyranosyl]-α-D-galactopyranosyl)-L-threonine *tert*-butyl ester (**13**) S22

3.4 *N*-(9*H*-Fluoren-9-yl)-methoxycarbonyl-*O*-(2-acetamido-2-deoxy-3-*O*-[β-D-4-deoxy-4-fluoro-galactopyranosyl]-α-D-galactopyranosyl)-L-threonine *tert*-butyl ester (**12**) S23

3.5 Enzymatic degradation assay S24

4 Immunological evaluation S26

4.1 Immunization procedure S26

4.2 Enzyme-linked immune sorbent assays S26

4.3 Analysis of the antibody binding to MCF-7 cells S29

5 NMR-Spectra S30

6 References S44

1 General Remarks

Solvents for moisture-sensitive reactions (toluene, MeCN, CH_2Cl_2, $MeNO_2$) were distilled and dried according to standard procedures. Glycosylations were performed in flame-dried glassware under inert argon atmosphere. DMF (amine-free, for peptide synthesis) and NMP were purchased from Roth, and Ac_2O in p.a. quality from Acros. Reagents were purchased in the highest available commercial quality and used as supplied except where noted. Fmoc-protected amino acids were purchased from Orpegen Pharma. For solid-phase synthesis, pre-loaded TentaGel S resin (Rapp Polymere) was employed. Reactions were monitored by TLC with pre-coated silica gel 60 F_{254} aluminium plates (Merck KGaA, Darmstadt). Flash column chromatography was performed with silica gel (230-400 mesh) from Merck. RP-HPLC analyses were performed on a JASCO-HPLC system with Phenomenex Luna C18(2) (250 x 4.6 mm, 10 μm), and Phenomenex Jupiter C18(2) (250 × 4.6 mm, 10 μm) columns at a flow rate of 1 $mLmin^{-1}$. Preparative HPLC separations were carried out on a JASCO-HPLC System with Phenomenex Luna C18(2) (250 × 30 mm, 10 μm), and Phenomenex Jupiter C18(2) (250 × 30 mm, 10 μm) columns at a flow rate of 20 $mLmin^{-1}$ or 10 $mLmin^{-1}$. Mixtures of H_2O–MeCN were used as solvents; if required 0.1% TFA were added.

Gradient **A**:

time (min)	0	10	25	60
acetonitrile (%)	50	50	77	100
water (%)	50	50	23	0

Gradient **B**:

time (min)	0	5	30	60
acetonitrile + 0.1% TFA (%)	30	30	90	100
water + 0.1% TFA (%)	70	70	10	0

Gradient **C**:

time (min)	0	1	40	60
acetonitrile + 0.1% TFA (%)	5	5	70	100
water + 0.1% TFA(%)	95	95	30	0

Gradient **D**:

time (min)	0	5	40	60
acetonitrile + 0.1% TFA (%)	5	5	25	70
water + 0.1% TFA (%)	95	95	75	30

^{1}H, ^{13}C, ^{19}F, and 2D NMR spectra were recorded on a Bruker AC-300 or a Bruker AM-400 spectrometer. The chemical shifts are reported in ppm relative to the signal of the deuterated solvent. Multiplicities are given as: s (singlet), br s (broad singlet), d (doublet), t (triplet), and m (multiplet). Assignment of proton and carbon signals was achieved by additional COSY, HMQC, and HMBC experiments when noted. The signals of the saccharide portions were denoted as follows: *N*-acetyl-D-galactosamine (no prime), and D-galactose ('). HR-ESI-mass spectra were recorded on a Micromass Q TOF Ultima 3 spectrometer, and optical rotations were measured at 546 nm and 578 nm with a Perkin-Elmer polarimeter 241.

2 Experimental Procedures

2.1 4-Methylphenyl 2,3,4,6-tetra-*O*-acetyl-1-thio-β-D-glucopyranoside (2)

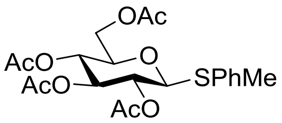

To a solution of 30.0 g (76.8 mmol, 1 eq.) penta-*O*-acetyl-α/β-D-glucopyranose (**1**) cooled to 0 °C in 150 mL abs. dichloromethane 15.2 g (122 mmol, 1.6 eq.) thiocresol and 14.0 mL $BF_3 \cdot Et_2O$ (100 mmol, 1.3 eq.) were added slowly. After the addition, the solution was allowed to warm to room temperature and was stirred 24h. The organic phase was neutralized with sat. aq. $NaHCO_3$ (3 × 70 mL), water (2 × 70 mL) and brine (50 mL). The organic layer was dried with $MgSO_4$, filtered and the solvent was removed under reduced pressure. The crude product was purified by flash chromatography on silica (^{c}Hex/EtOAc, 2:1).

Yield: 28.3 g (62.2 mmol**,** 81%), pale yellow solid. $[\alpha]_D^{22}$ = (1.00, $CHCl_3$) = -11.0. **R*f*** =0.32 (^{c}Hex/EtOAc, 2:1). $C_{21}H_{26}O_9S$ (M = 454,491 g/mol). **^{1}H-NMR** (400 MHz, HSQC, $CDCl_3$), δ [ppm] = 7.38 (d, J = 8.0 Hz, 2H, H_{Ar}), 7.12 (d, J = 7.8 Hz, 2H, H_{Ar}), 5.20 (pt, $J_{H3,H2}$ = $J_{H3,H4}$ = 9.4 Hz, 1H, H-3), 5.02 (dd, $J_{H4,H5}$ = 10.0 Hz, $J_{H4,H3}$ = 9.6 Hz, 1H, H-4), 4.93 (dd, $J_{H2,H1}$ = 10.1 Hz, $J_{H2,H3}$ = 9.3 Hz, 1H, H-2), 4.63 (d, $J_{H1,H2}$ = 10.1 Hz, 1H, H-1), 4.25–4.13 (m, 2H, H-$6_{a,b}$), 3.69 (ddd, $J_{H5,H4}$ = 10.1 Hz, $J_{H5,H6a}$ = 4.8 Hz, $J_{H5,H6b}$ = 2.7 Hz, 1H, H-5), 2.34 (s, 3H, -SPh*Me*), 2.08, 2.08, 2.01, 1.98 (4 × s, 12H, -OAc). **^{13}C-NMR** (100 MHz, $CDCl_3$), δ [ppm] = 170.7, 170.3, 169.5, 169.4 (4 × C=O), 138.9, 133.9 (2 × C_qAr), 130.0, 129.9, 129.8, 127.6 (4 × C_{Ar}), 85.9 (C-1), 75.8 (C-5), 74.1 (C-3), 70.0 (C-2), 68.3 (C-4), 62.2 (C-6), 21.3 (-SPh*Me*), 20.9, 20.8, 20.7, 20.6 (-OAc). **ESI-MS** (*positive*), *m/z*: 415.1029 ($[M+Na]^+$, calc.: 415.1033).

Further analytical data, see literature.[1]

2.2 4-Methylphenyl 2,3-di-*O*-acetyl-4,6-*O*-benzylidene-1-thio-β-D-gluco-pyranoside (3)

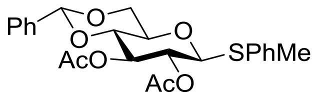

To a stirred solution of 22.0 g (48.4 mmol, 1 eq.) 4-methylphenyl-2,3,4,6-tetra-*O*-acetyl-1-thio-β-D-glucopyranoside **(2)** in 50 mL methanol were added 800 mg (14.8 mmol, 0.3 eq.) sodium methanolate at room temperature. After complete conversion within 12 h, the reaction mixture was neutralized with *Amberlite IR120* and the solvent was removed under reduced pressure. The crude product was co-evaporated with toluene (2 × 20 mL) and was used without further purification.

The crude product was dissolved in 40 mL of a 1:1 mixture of abs. DMF and abs. acetonitrile. Then, 7.97 mL (53.2 mmol, 1.1 eq.) benzaldehyde dimethylacetal and 400 mg *p*-toluene-sulfonic acid were added. The mixture was stirred at 60 °C for 18 h, before it was neutralized with 10 ml triethylamine. The solvent was removed under reduced pressure and the crude product was used without further purification.
ESI-MS (*positive*), *m/z*: 397.1083 ($[M+Na]^+$, calc.: 397.1080).

To a solution of crude methylphenyl-4,6-benzylidene-1-thio-β-D-glucopyranoside in 80 mL abs. pyridine were added 200 mg DMAP and 30 mL Ac_2O at room temperature. The reaction mixture was stirred for 12 h. After complete conversion, the solution was diluted with 100 mL CH_2Cl_2 and poured into ice water. The organic layer was washed with 1 M HCl (2 × 50 mL), sat. aq. $NaHCO_3$ (2 × 70 mL) and brine (80 mL) and was dried with $MgSO_4$. The solvent was removed under reduced pressure and the crude product was purified by flash chromatography on silica (^{c}Hex/EtOAc, 2:1).

Yield: 18.8 g (41.0 mmol, 85% over 3 steps). $[\alpha]_D^{22}$ = (1.00, $CHCl_3$) = -41.6. **R*f*** = 0.61 (^{c}Hex/EtOAc, 2:1). $C_{24}H_{26}O_7S$ (M =458,524 g/mol). **^{1}H-NMR** (400 MHz, COSY, HMBC, HSQC, $CDCl_3$), δ [ppm] = 7.44–7.32 (m, 7H, H_{Ar}), 7.18–7.10 (m, 2H, H_{Ar}), 5.48 (s, 1H, C*H*Ph), 5.32 (dd, $J_{H3,H4}$ = 9.6 Hz, $J_{H3,H2}$ = 8.9 Hz, 1H, H-3), 4.97 (dd, $J_{H2,H1}$ = 10.0 Hz, $J_{H2,H3}$ = 8.9 Hz, 1H, H-2), 4.73 (d, $J_{H1,H2}$ = 10.0 Hz, 1H, H-1), 4.38 (dd, J = 10.5 Hz, J_H = 4.9 Hz, 1H, H-6_a), 3.78 (dd, J = 10.6 Hz, J = 9.8 Hz, 1H, H-6_b), 3.64 (t, J = 9.5 Hz, 1H, H-4), 3.55 (td, J = 9.6 Hz, J_H = 4.9 Hz, 1H, H-5), 2.36 (s, 3H, -SPh*Me*), 2.11, 2.03 (2 × s, 6H, -OAc). **^{13}C-**

NMR (100 MHz, $CDCl_3$), δ [ppm] =.170.3, 169.7 (2 × C=O(OAc)), 139.0, 136.9, 133.8, 129.9, 129.3, 128.4, 127.8, 126.3 (12 × C_{Ar}), 101.6 (-*C*HPh), 86.9 (C-1), 78.2 (C-4), 73.1 (C-3), 70.9 (C-2), 70.8 (C-5), 68.6 (C-6), 21.3 (-SPh*Me*), 21.0, 20.9 (2 × -OAc). **EI-MS** (*negative*), *m/z*: 458.1387 ($[M-H]^-$, calc.: 458.1399).
Further analytical data, see literature.[2]

2.3 4-Methylphenyl 2,3-di-*O*-acetyl-1-thio-β-D-glucopyranoside (4)

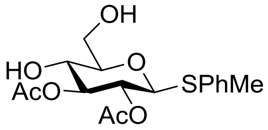

A solution of 16.9 g (36.9 mmol) 4-methylphenyl-2,3-di-*O*-acetyl-4,6-*O*-benzylidene-1-thio-β-D-glucopyranoside **(3)** in 200 ml aq. AcOH (80%) was stirred at 90 °C for 3 h. The solvent was removed under reduced pressure and the residue was co-evaporated with toluene (2 × 50 mL). The crude product was purified by flash chromatography on silica (EtOAc).

Yield: 12.8 g (34.4 mmol, 93%). $[\alpha]_D^{22}$ = (1.00, $CHCl_3$) = -22.2. **R*f*** = 0.12 (^{c}Hex/EtOAc, 2:1). $C_{17}H_{22}O_7S$ (M = 370,417 g/mol). **^{1}H-NMR** (400 MHz, COSY, HMBC, HSQC, $CDCl_3$), δ [ppm] = 7.35 (d, *J* = 8.0 Hz, 2H, H_{Ar}), 7.12(d, *J* = 8.51 Hz, 2H, H_{Ar}), 5.05 (t, $J_{H3,H2} = J_{H3,H4}$ = 9.3 Hz, 1H, H-3), 4.88 (dd, $J_{H2,H1}$ = 10.0 Hz, $J_{H2,H3}$ = 9.3 Hz, 1H, H-2), 4.67 (d, $J_{H1,H2}$ = 10.0 Hz, 1H, H-1), 3.92 (dd, $J_{H6a,H6b}$ = 12.1 Hz, $J_{H6a,H5}$ = 3.3 Hz, 1H, H-6_a), 3.80 (dd, $J_{H6b,H6a}$ = 12.1 Hz, $J_{H6b,H5}$ = 4.6 Hz, 1H, H-6_b), 3.69 (t, $J_{H4,H3} = J_{H4,H5}$ = 9.5 Hz, 1H, H-4), 3.42 (ddd, $J_{H5,H4}$ = 9.7 Hz, $J_{H5,H6b}$ = 4.6 Hz, $J_{H5,H6a}$ = 3.3 Hz, 1H, H-5), 2.33 (s, 3H, -SPh*Me*), 2.09, 2.06 (2 × s, 6H, -OAc). **^{13}C-NMR** (100 MHz, $CDCl_3$), δ [ppm] = 171.6, 169.7 (2 × C=O(OAc)), 138.8, 133.4, 129.9, 128.1 (4 × C_{Ar}), 86.1 (C-1), 79.7 (C-5), 77.1 (C-3), 70.2 (C-2), 69.2 (C-4), 62.3 (C-6), 21.3 (-SPh*Me*), 21.0 (2 × -OAc). **EI-MS** (*positive*), *m/z*: 370.1088 ($[M]^{\cdot+}$, calc.: 370.1086).
Further analytical data, see literature.[3]

2.4 4-Methylphenyl 2,3-di-*O*-acetyl-6-*O*-trityl-1-thio-β-D-glucopyranoside (5)

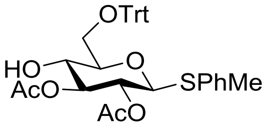

To a stirred solution of 9.55 g (25.8 mmol, 1 eq.) 4-methylphenyl-2,3-di-*O*-acetyl-1-thio-β-D-glucopyranoside **(4)** in 70 mL abs. pyridine were added 200 mg DMAP and 7.19 g (25.8 mmol, 1 eq.) triphenylmethyl chloride. The reaction mixture was stirred for 24 h at 50 °C. The solvent was removed under reduced pressure and the residue was taken up in 100 mL CH_2Cl_2. The organic phase was washed with 1 M HCl (50 mL), sat. aq. $NaHCO_3$ (2 × 70 mL), brine (100 mL) and was dried with $MgSO_4$. The solvent was removed under reduced pressure and the crude product was purified by flash chromatography on silica (^{c}Hex/EtOAc, 1:1).

Yield: 14.1 g (22.9 mmol, 89%). $[\alpha]_D^{22}$ = (1.00, $CHCl_3$) = -22.8. **R*f*** = 0.48 (^{c}Hex/EtOAc, 2:1). $C_{36}H_{36}O_7S$ (M = 612,732 g/mol). **^{1}H-NMR** (400 MHz, COSY, HMBC, HSQC, $CDCl_3$), *δ* [ppm] = 7.48–7.44 (m, 7H, H_{Ar}), 7.32–7.25 (m, 10H, H_{Ar}), 7.09 (d, *J* = 7.9 Hz, 2H, H_{Ar}), 5.02 (pt, $J_{H4,H3}$ = $J_{H4,H5}$ = 9.3 Hz, 1H, H-4), 4.91 (dd, $J_{H3,H2}$ = 9.9 Hz, $J_{H3,H4}$ = 9.3 Hz, 1H, H-3), 4.62 (pd, $J_{H2,H3}$ = $J_{H2,H1}$ = 9.9 Hz, 1H, H-2), 3.72 (pt, $J_{H5,H4}$ = $J_{H5,H6a/b}$ = 9.1 Hz, 1H, H-5), 3.53–3.47 (m, 1H, H-6$_a$), 3.43–3.37 (m, 2H, {3.40, d, $J_{H1,H2}$ = 8.8 Hz, H-1}, H-6$_b$), 2.34 (s, 3H, -SPh*Me*), 2.10, 2.04 (2 × s, 6H, -OAc). **^{13}C-NMR** (100 MHz, $CDCl_3$), *δ* [ppm] = 171.3, 169.7 (2 × C=O(OAc)), 143.7, 138.57, 133.8, 129.8, 128.8, 128.1, 127.3 (25 × C_{Ar}), 87.2 (C_q), 85.8 (C-2), 78.7 (C-1), 77.0 (C-4), 70.1 (H-3), 70.0 (C-5), 63.5 (C-6), 21.3 (-SPh*Me*), 21.0 (2 × -OAc). **EI-MS** (positive), *m/z*: 612.2187 ([M]$^{\cdot+}$, calc.: 612.2182.

2.5 4-Methylphenyl 2,3-di-*O*-acetyl-4-deoxy-4-fluoro-6-*O*-trityl-1-thio-β-D-glucopyranoside (6)

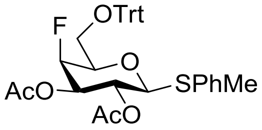

A stirred solution of 7.23 g (11.8 mmol, 1 eq) 4-methylphenyl-2,3-di-*O*-acetyl-6-*O*-trityl-1-thio-β-D-glucopyranoside **(5)** in 100 mL abs. CH_2Cl_2 and 20 mL abs. pyridine was cooled to 0 °C and 2.09 mL (14.2 mmol, 1.2 eq.) trifluoromethanesulfonic anhydride were added drop wise. The reaction mixture was stirred for 2 h, before it was diluted with 100 mL CH_2Cl_2. The organic phase was washed with 1 M HCl (40 mL), sat. aq. $NaHCO_3$ (2 × 60 mL), water (50 mL) and was dried with $MgSO_4$. The solvent was removed under reduced pressure and the crude product was used without further purification.

Meanwhile, 4.84 g (15.3 mmol, 1.3 eq.) TBAF·3 H_2O were co-evaporated with abs. toluene (2 × 20 mL) and abs. THF (30 mL) and the residue was taken up in abs. THF (50 mL). After addition of activated molecular sieves (4 Å, 2.00 g) the mixture was stirred for 2 h at room temperature. The crude triflate, dissolved in abs. THF (30 mL), was added drop wise and the mixture was again stirred for 3 h at room temperature. The molecular sieves (4Å) were filtered off, the solvent was removed under reduced pressure and the crude product was purified by flash chromatography on silica (^{c}Hex/EtOAc, 3:1).

Yield: 5.06 g (8.23 mmol, 70%). $[\alpha]_D^{22}$ = (1.00, $CHCl_3$) = +18.2. **R*f*** = 0.59 (^{c}Hex/EtOAc, 2:1). $C_{36}H_{35}FO_6S$ (M = 614,723 g/mol). **^{1}H-NMR** (400 MHz, COSY, HMBC, HSQC, $CDCl_3$), *δ* [ppm] = 7.46–7.42 (m, 5H, H_{Ar}), 7.40 (d, *J* = 8.1 Hz, 2H, H_{Ar}), 7.34–7.24 (m, 10H, H_{Ar}), 7.09 (d, *J* = 7.9 Hz, 2H, H_{Ar}), 5.22 (pd, $J_{H2,H1}$ = $J_{H2,H3}$ = 10.0 Hz, 1H, H-2), 4.98 (ddd, $J_{H3,F}$ = 27.7 Hz, $J_{H3,H2}$ = 9.9 Hz, $J_{H3,H4}$ = 2.7 Hz, 1H, H-3), 4.88 (dd, $J_{H4,F}$ = 50.0 Hz, $J_{H4,H3}$ = $J_{H4,H5}$= 2.7 Hz, 1H, H-4), 4.62 (d, $J_{H1,H2}$ = 10.0 Hz, 1H, H-1), 3.63–3.50 (m, 2H, H-5, H-6_a), 3.30 (td, $J_{H6b,H6a}$ = 8.8 Hz, $J_{H6b,H5}$ = 2.5 Hz, 1H, H-6_b), 2.33 (s, 3H, -SPh*Me*), 2.10, 2.09 (2 × s, 6H, -OAc). **^{13}C-NMR** (100 MHz, $CDCl_3$), *δ* [ppm] = 170.4, 169.1 (2 × C=O(OAc)), 143.7, 138.4, 133.1, 129.9, 128.8, 128.7, 128.1, 127.3 (24 × C_{Ar}), 87.3 (C_q), 86.8 (C-1), 86.7 (d, $J_{C4,F}$ = 185.6 Hz, C-4)), 76.4 (d, $J_{C5,F}$ = 18.1 Hz, C-5), 72.7 (d, $J_{C3,F}$ = 17.7 Hz, C-3) , 67.6 (C-2), 61.8 (C-6), 21.3 (-SPh*Me*), 21.0, 20.9 (2 × -OAc). **EI-MS** (positive), *m/z*: 614.2145 $([M+Na]^+$, calc.: 614.2138.

2.6 4-Methylphenyl 2,3,6-tri-*O*-acetyl-4-deoxy-4-fluoro-1-thio-β-D-glucopyranoside (7)

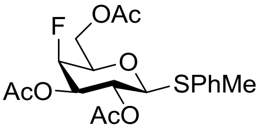

A solution of 4.57 g (7.43 mmol) 4-methylphenyl-2,3-di-*O*-acetyl-4-deoxy-4-fluoro-6-*O*-trityl-1-thio-β-D-glucopyranoside **(6)** in 200 mL aq. AcOH (80%) was stirred for 4 h at 90 °C. After complete conversion, the solvent was removed under reduced pressure and the residue was co-evaporated with toluene (2 × 30 mL). The crude product was used without further purification.

To a solution of the crude product in 80 mL abs. pyridine, 20 mL of Ac_2O and 100 mg DMAP were added and the mixture was stirred for 12 h at room temperature. The reaction mixture

was diluted with 100 mL CH_2Cl_2, poured onto ice water and the organic layers were washed with 1 M HCl (2 × 50 mL), sat. aq. $NaHCO_3$ (2 × 70 mL), water (2 × 50 mL) and brine (100 mL). The organic phase was dried with $MgSO_4$, the solvent was removed under reduced pressure and the crude product was purified by flash chromatography on silica (^{c}Hex/EtOAc, 3:1).

Yield: 2.40 g (5.79 mmol, 78% over 2 steps). $[\alpha]_D^{22}$ = (1.00, $CHCl_3$) = +24.6. **R*f*** = 0.66 (^{c}Hex/EtOAc, 1:1). $C_{19}H_{23}FO_7S$ (M = 414,445 g/mol). **^{1}H-NMR** (400 MHz, COSY, HMBC, HSQC, $CDCl_3$), δ [ppm] = 7.39 (d, J = 8.1 Hz, 2H, H_{Ar}), 7.11 (d, J = 7.9 Hz, 1H, H_{Ar}), 5.24 (pd, $J_{H2,H1}$ = $J_{H2,H3}$ = 10.0 Hz, 1H, H-2), 4.98 (ddd, $J_{H3,F}$ = 27.7 Hz, $J_{H3,H2}$ = 10.0 Hz, $J_{H3,H4}$ = 2.6 Hz, 1H, H-3), 4.85 (dd, $J_{H4,F}$ = 50.3 Hz, $J_{H4,H3}$ = 2.6 Hz, 1H, H-4), 4.63 (dd, $J_{H1,H2}$ = 10.0 Hz, 1H, H-1), 4.36 (ddd, $J_{H6a,H6b}$ = 11.4 Hz, $J_{H6a,H5}$ = 6.8 Hz, $J_{H6a,F}$ = 1.1 Hz, 1H, H-6_a), 4.21 (dd, $J_{H6b,H6a}$ = 11.4 Hz, $J_{H6b,H5}$ = 6.3 Hz, 1H, H-6_b), 3.81 (dt, $J_{H5,F}$ = 26.4 Hz, $J_{H5,H6a/b}$ = 6.7 Hz, 1H, H-5), 2.33 (s, 3H, -SPh*Me*), 2.10, 2.09, 2.07 (3 × s, 9H, -OAc). **^{13}C-NMR** (100 MHz, $CDCl_3$), δ [ppm] =.170.5, 170.4, 169.3 (3 × C=O(OAc)), 138.7, 133.6, 129.8, 128.1 (4 × C_{Ar}), 86.6 (C-1), 86.1 (d, $J_{C4,F}$ = 186.0 , C-4), 74.6 (d, $J_{C5,F}$ = 18.3 Hz, C-5), 72.6 (d, $J_{C3,F}$ = 17.7 Hz, C-3), 67.3 (C-2), 61.7 (d, $J_{C6,F)}$ = 5.6 Hz, C-6), 21.3 (-SPh*Me*), 20.9, 20.8 (3 × -OAc). **EI-MS** (positive), *m/z*: 414.1151 ($[M]^{\cdot+}$, calc.: 414.1149.

2.7 *N*-(9*H*-Fluoren-9-yl)-methoxycarbonyl-*O*-(2-acetamido-2-deoxy-4,6-*O*-benzylidene-3-*O*-[2,3,6-tri-*O*-acetyl-β-D-4-deoxy-4-fluoro-galacto-pyranosyl]-α-D-galactopyranosyl)-L-threonine *tert*-butyl ester (8)

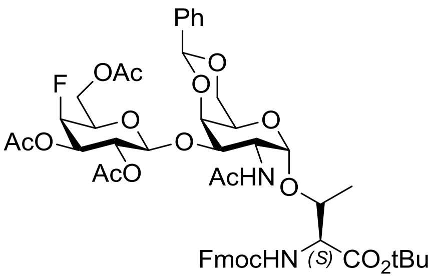

A mixture of 518 mg (0.75 mmol, 1 eq.) Fmoc-Thr-(α-4,6-Bzn-GalNAc)-O*t*Bu and 625 mg (0.90 mmol, 1.2 eq.) thiodonor **7** in 15 mL abs. CH_2Cl_2 were stirred for 30 min in the presence of freshly activated molecular sieves (4Å, 1.50 g). The reaction mixture was cooled to 0 °C, before 255 mg (0.67 mmol, 1.4 eq.) *N*-Iodsuccinimide and 83 µg (0.19 mmol, 0.4 eq.) AgOTf were added. The mixture was allowed to warm to room temperature and stirred for 24 h. The reaction mixture was diluted with 50 ml CH_2Cl_2, filtered through Celite and washed with sat. aq. $NaHCO_3$ (70 mL), sat. aq. $Na_2S_2O_3$ (50 mL) and brine (50 mL). The organic phase was dried with $MgSO_4$, the solvent was removed under reduced pressure and the crude product was purified by flash chromatography on silica (^{c}Hex/EtOAc, 2:3).

Yield: 580 mg (0.60 mmol, 80%), colorless amorphous solid. $[\alpha]_D^{23}$ = (1.00, $CHCl_3$) = +59.39. **R*f*** = 0.24 (^{c}Hex/EtOAc, 2:3). ***RP-HPLC*** (Phenomenex Luna C18, λ = 264 nm): t_R= 25.9 min.

time (min)	0	10	25	60
acetonitrile (%)	50	50	77	100
water (%)	50	50	23	0

$C_{50}H_{59}FN_2O_{17}$ (M=979.005 g/mol). **^{1}H-NMR** (400 MHz, $CDCl_3$, COSY, HMQC), δ [ppm] = 7.76 (d, 2H, $J_{H4,H3}$ = $J_{H5,H6}$ = 7.54 Hz, H-4/H-5-Fmoc), 7.61 (d, 2H, $J_{H1,H2}$ = $J_{H8,H7}$ = 6.05 Hz, H-1/H-8-Fmoc), 7.51 (dd, 2H, $J_{H,H}$ = 7.90 Hz, $J_{H,H}$ = 1.54 Hz, H_{Ar}), 7.39 (t, 2H, $J_{H3,H4}$ = $J_{H6,H5}$ = 6.86 Hz, H-3/H-6-Fmoc), 7.36–7.26 (m, 5H, 2-H-, H-7-Fmoc, H_{Ar}), 5.91 (d, 1H, $J_{NH,H2}$ = 9.54 Hz, NH-Ac), 5.84 (d, 1H, $J_{NH,T\alpha}$ = 9.44 Hz, NH-urethane), 5.52 (s, 1H, C*H*Ph), 5.23 (dd, 1H, $J_{H2',H1'}$ = 9.90 Hz, $J_{H2',H3'}$ = 8.27 Hz, H-2'), 4.97–4.71 (m, 4H, 1-H, H-1', H-3', H-4'), 4.71–4.62 (dd, 1H, $J_{H2,NH}$ = 9.75 Hz, $J_{H2,H3}$ = 8.27 Hz, $J_{H2,H1}$ = 3.55 Hz, H-2), 4.68–4.60 (m,

3H, CH_2-Fmoc, H-6a’), 4.32–4.10 (m, 6H, T^{α}, CH-Fmoc, H-4, T^{β}, H-6a, H-6b’), 4.10–3.93 (m, 2H, H-3, H-6b), 3.80 (ddd, 1H, $J_{H5',F}$ = 26.33 Hz, $J_{H5',H6a'}$ = 6.22 Hz, $J_{H2',H6b'}$ = 6.22 Hz, H-5’), 3.73–3.60 (m, 1H, H-5), 2.07, 2.03, 1.99, 1.98 (4 × s, 12H, -OAc), 1.43 (s, 9H 3 × CH_3(*t*Bu)), 1.26 (d, 3H, $J_{T\gamma,T\beta}$ = 5.94 Hz, T^{γ}). **^{13}C-NMR** (100.6 MHz, DEPT, HMQC, $CDCl_3$), δ [ppm] = 170.5 (C=O(NHAc)), 170.0, 169.8, 169.6 (3 × C=O(OAc, Ester)), 156.7 (C=O(urethane)), 143.8, 143.8 (C-1a-, C-8a-Fmoc), 141.3 (C-4a-, C-5a-Fmoc), 137.6 (C*q*), 128.8, 128.1, 127.8 (C_{Ar}), 127.1 (C-3-, C-6-Fmoc), 126.3 (C-2-, C-7-Fmoc), 126.0 (C_{Ar}), 125.0 (C-1-, C-8-Fmoc), 120.1 (C-4-, C-5-Fmoc), 100.8 (*C*HPh), 100.6 (C-1), 100.2 (C-1’), 85.9 (d, $J_{C4',F}$ = 186.62 Hz, C-4’), 83.1 (C*q*(*t*Bu)), 76.3 (T^{β}), 75.5 (C-4), 72.5 (C-3), 71.4 (d, 2C, $J_{C5',F}$ = 17.73 Hz, $J_{C3',F}$ = 17.73 Hz, C-3’, C-5’), 69.1 (C-6), 68.5 (C-2’), 66.9 (CH_2-Fmoc), 63.7 (C-5), 61.6 (d, $J_{C6',F}$ = 5.15 Hz, C-6’), 59.2 (T^{α}), 47.8 (C-2), 47.3 (CH-(Fmoc)), 28.1 (CH_3 (*t*Bu)), 20.8, 20.7, 20.7, 20.7 (4 × -OAc), 19.2 (T^{γ}). **^{19}F-NMR** (376.5 MHz, $CDCl_3$), δ [ppm] = -215.9–-216.6 (m, $J_{F,H4'}$ = 50.29 Hz, $J_{F,H5'}$ = 26.69 Hz, $J_{F,H3'}$ = 25.83 Hz; a further set of signals indicates the presence of a rotamer). **ESI-MS** (positive), *m/z*: 979.43 ($[M]^+$, calc.: 979.38), 1001.35 ($[M+Na]^+$, calc.: 1001.37), 1979.80 ($[2 \times M+Na]^+$, calc.: 1979.75). **HR-ESI-MS** (positive), *m/z*: 1001.3712 ($[M+Na]^+$, calc.: 1001.3695).

2.8 *N*-(9*H*-Fluoren-9-yl)-methoxycarbonyl-*O*-(2-acetamido-2-deoxy-3-*O*-[2,3,6-tri-*O*-acetyl-β-D-4-deoxy-4-fluoro-galactopyranosyl]-*α*-D-galactopyranosyl)-L-threonine *tert*-butyl ester (9)

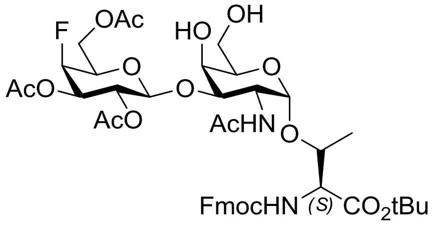

To a solution of disaccharide **8** (560 mg, 0.57 mmol) in a mixture of CH_2Cl_2 and MeOH (4:1, 75 mL) were added $NaHSO_4$–SiO_2 (500 mg) and the suspension was stirred for 18 h at room temperature. The catalyst was filtered off and the filtrate was washed with sat. aq. $NaHCO_3$ (3 × 50 mL) and brine (50 mL), dried over $MgSO_4$, filtered and concentrated under reduced pressure. The crude product was purified by flash chromatography on silica (EtOAc).

Yield: 484 mg (0.54 mmol, 90%) colorless amorphous solid. $[\alpha]_D^{23}$ = (1.00, $CHCl_3$) = +35.6. $\mathbf{R_f}$ = 0.23 (EtOAc). ***RP-HPLC*** (Phenomenex Luna C18, λ = 264 nm): t_R= 16.1 min.

TIME (min)	0	10	25	60
AcetonitrilE (%)	50	50	77	100
water (%)	50	50	23	0

$C_{43}H_{55}FN_2O_{17}$ (M = 890.898 g/mol). **^{1}H-NMR** (400 MHz, $CDCl_3$) δ [ppm] = 7.72 (d, 2H, $J_{H4,H3}$ = $J_{H5,H6}$ = 7.52 Hz, H-4, H-5-Fmoc), 7.57 (d, 2H, $J_{H1,H2}$ = $J_{H8,H7}$ = 6.57 Hz, 1-H-, H-8-Fmoc), 7.35 (t, 2H, $J_{H3,H4}$ = $J_{H6,H5}$ = 7.44 Hz, 3-H-, H-6-Fmoc), 7.26 (t, 2H, $J_{H2,H1}$ = $J_{H7,H8}$ = 7.15 Hz, 2-H-, H-7-Fmoc), 6.19 (d, 1H, $J_{NH,H2}$ = 9.55 Hz, NH-Ac), 5.83 (d, 1H, $J_{NH,T\alpha}$ = 9.04 Hz, NH-urethane), 5.20–5.01 (m, 1H, H-2'), 5.00–4.66 (m, 3H, H-1, H-3', H-4'), 4.59 (d, 1H, $J_{H1',H2'}$ = 7.68 Hz, H-1'), 4.53–4.23 (m, 4H, 2-H, H-6a', CH_2-Fmoc), 4.24–3.98 (m, 5H, H-4, H-6b', T^α, T^β, CH-Fmoc), 3.93–3.72 (m, 4H, H-6a/b, H-5, H-5'), 3.72–3.59 (m, 1H, H-3), 2.03, 2.03, 1.98, 1.95 (4 × s, 12H, -OAc), 1.40 (s, 9H; 3 × CH_3(*t*Bu)), 1.24 (d, 3H, $J_{T\gamma,T\beta}$ = 5.78 Hz, T^γ). **^{13}C-NMR** (100.6 MHz, DEPT, HMQC, $CDCl_3$), δ [ppm] = 170.6 (C=O(NHAc)), 170.4, 170.1, 169.7 (3 × C=O(OAc, Ester)), 156.6 (C=O(urethane)), 143.7, 143.7 (C-1a-, C-8a-Fmoc), 141.3 (C-4a-, C-5a-Fmoc), 127.8 (C-3-, C-6-Fmoc), 127.1 (C-2-, C-7-Fmoc), 125.0 (C-1-, C-8-Fmoc), 120.1 (C-4-, C-5-Fmoc), 101.3 (C-1'), 100.0 (C-1), 85.8 (d, $J_{C3',F}$ = 186.30 Hz, C-4'), 83.0 (C*q*(*t*Bu)), 77.8 (C-3), 76.0 (T^β), 71.1 (d, $J_{C5',F}$ = 17.46 Hz, C-5'), 70.8 (d, $J_{C3',F}$ = 17.99 Hz, C-3'), 70.1 (C-5), 69.3 (C-4), 68.5 (C-2'), 66.9 (CH_2-Fmoc), 62.4 (C-6), 61.2 (d, $J_{C6',F}$ = 3.78 Hz, C-6'), 59.2 (T^α), 47.7 (C-2), 47.2 (CH-(Fmoc)), 28.0 (CH_3(*t*Bu)), 20.6 (4 × -OAc), 18.8 (T^γ). **^{19}F-NMR** (376.5 MHz, $CDCl_3$), δ [ppm] = -216.1 – -216.4 (m, $J_{F,H4'}$ = 50.65 Hz, $J_{F,H5'}$ = 27.26 Hz, $J_{F,H3'}$ = 25.97 Hz; a further set of signals indicates the presence of a rotamer). **ESI-MS** (positive), *m/z*: 913.36 ($[M+Na]^+$, calc.: 913.34), 1803.74 ($[2 \times M+Na]^+$, calc.: 1803.69). **HR-ESI-MS** (positive), *m/z*: 913.3371 ($[M+Na]^+$, calc.: 913.3382).

2.9 *N*-(9*H*-Fluoren-9-yl)-methoxycarbonyl-*O*-(4,6-di-*O*-acetyl-2-acetamido-2-deoxy-3-*O*-[2,3,6-tri-*O*-acetyl-β-D-4-deoxy-4-fluoro-galactopyranosyl]-α-D-galactopyranosyl)-L-threonine *tert*-butyl ester (10)

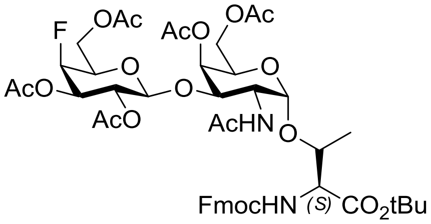

To a solution of 286 mg (0.32 mmol, 1.0 eq.) disaccharide **9** in 10 mL of pyridine were added 5 mL acetic anhydride and the solution was stirred 12 h at ambient temperature. The solvent was removed under reduced pressure and the residue was co-evaporated with toluene (3 × 30 mL). The crude product was purified by flash chromatography on silica (^{c}Hex/EtOAc, 1:3).

Yield: 301 mg (0.31 mmol, 96%) colorless amorphous solid. $[\alpha]_D^{23}$ = (1.00, $CHCl_3$) = +43.1. $\mathbf{R_f}$ = 0.38 (^{c}Hex/EtOAc, 1:3). ***RP-HPLC*** (Phenomenex Luna C18, λ = 264 nm): t_R= 22.8 min.

time (min)	0	10	25	60
acetonitrile (%)	50	50	77	100
water (%)	50	50	23	0

$C_{47}H_{59}FN_2O_{19}$ (M = 974.972 g/mol). **^{1}H-NMR** (400 MHz, $CDCl_3$) δ [ppm] = 7.76 (d, 2H, $J_{H4,H3}$ = $J_{H5,H6}$ = 7.44 Hz, H-4-, H-5-Fmoc), 7.60 (d, 2H, $J_{H1,H2}$ = $J_{H8,H7}$ = 6.89 Hz, H-1-, H-8-Fmoc), 7.40 (t, 2H, $J_{H3,H4}$ = $J_{H6,H5}$ = 7.34 Hz, 3-H-, H-6-Fmoc), 7.31 (t, 2H, $J_{H2,H1}$ = $J_{H7,H8}$ = 6.43 Hz, 2-H-, H-7-Fmoc), 6.01 (d, 1H, $J_{NH,H2}$ = 9.36 Hz, NH-Ac), 5.85 (d, 1H, $J_{NH,T\alpha}$ = 9.04 Hz, NH-urethane), 5.33 (s, 1H, H-4), 5.18–5.11 (m, 1H, H-2'), 5.00–4.67 (m, 3H, 1-H, H-3', H-4'), 4.60 (d, 1H, $J_{H1',H2'}$ = 7.55 Hz, H-1'), 4.56 – 4.43 (m, 3H, H-2, CH_2-Fmoc), 4.37 (dd, 1H, $J_{H6a',H6b'}$ = 11.19 Hz, $J_{H6a',H5}$ = 5.69 Hz, H-6a'), 4.29 – 4.05 (m, 6H, H-5, H-6b', H-6a, T^a, T^β, CH-Fmoc), 4.02–3.89 (m, 1H, H-6b), 3.88–3.68 (m, 2H, H-3, H-5'), 2.10, 2.07, 2.07, 2.05, 2.03, 1.99 (6 × s, 18H, -OAc), 1.43 (3 × s, 9H, CH_3(*t*Bu)), 1.28 (d, 3H, $J_{T\gamma,T\beta}$ = 6.00 Hz, T^γ). **^{13}C-NMR** (100.6 MHz, DEPT, HMQC, $CDCl_3$), δ [ppm] = 170.8 (C=O(NHAc)), 170.7, 170.3, 170.3, 170.2, 169.8 (5 × C=O(OAc, Ester)), 156.7 (C=O(urethane)), 143.9 (C-1a-, C-8a-Fmoc), 141.6 (C-4a-, C-5a-Fmoc), 128.1 (C-3-, C-6-Fmoc), 127.3 (C-2-, C-7-Fmoc), 125.1 (C-1-, C-8-Fmoc), 120.3 (C-4-, C-5-Fmoc), 100.7 (C-1'), 100.4 (C-1), 85.7 (d, $J_{C4',F}$ = 187.41 Hz, C-4'), 83.5 (C*q*(*t*Bu)), 77.4 (T^β), 72.8 (C-3), 71.5 (d, $J_{C3',F}$ = 17.73 Hz, C-3'), 71.1 (d, $J_{C5',F}$ = 18.07 Hz, C-5'), 69.3 (C-4), 68.5 (C-2'), 68.1 (C-

5), 67.2 (CH_2-Fmoc), 63.0 (C-6), 63.4 (d, $J_{C6',F}$ = 5.77 Hz, C-6'), 59.3 (T^{α}), 48.6 (C-2), 47.4 (CH-(Fmoc)), 28.3 (CH_3(*t*Bu)), 20.7 (6 × CH_3(OAc)), 18.9 (T^{γ}). **^{19}F-NMR** (376.5 MHz, $CDCl_3$), δ [ppm] = -216.4 (dt, $J_{F,H4'}$ = 51.58 Hz, $J_{F,H5'}$ = 26.63 Hz, $J_{F,H3'}$ = 26.63 Hz), -217.2 (dt, $J_{F,H4'}$ = 51.58 Hz, $J_{F,H5'}$ = 26.63 Hz, $J_{F,H3'}$ = 26.63 Hz); Two conformational isomers were observed. **ESI-MS** (positive), *m/z*: 997.31 ([M+Na]$^+$, calc.: 997.36), 1971.72 ([2 × M+Na]$^+$, calc.: 1971.73). **HR-ESI-MS** (positive), *m/z*: 997.3576 ([M+Na]$^+$, calc.: 997.3594).

2.10 *N*-(9*H*-Fluoren-9-yl)-methoxycarbonyl-*O*-(4,6-di-*O*-acetyl-2-acetamido-2-deoxy-3-*O*-[2,3,6-tri-*O*-acetyl-β-D-4-desoxy-4-fluorgalactopyranosyl]-α-D-galactopyranosyl)-L-threonine (11)

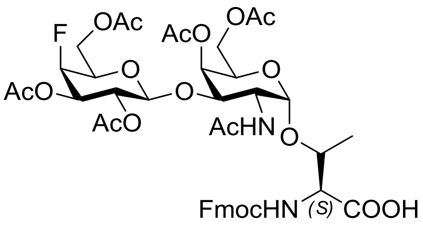

To a mixture of 301 mg (0.31 mmol, 1.0 eq.) of the fully protected disaccharide **10** in 0.6 mL water were added 6.0 mL TFA and the solution was stirred for 2.5 h at room temperature. The mixture was subsequently co-evaporated with toluene (5 × 30 mL) and dichloromethane (3 × 30 mL) and the residue was purified by flash chromatography on silica (EtOAc/MeOH/AcOH, 9:1:0.1).

Yield: 278 mg (0.30 mmol, 98%) colorless amorphous solid. **R_f** = 0.13 (EtOAc/MeOH/HOAc, 9:1:0.1). ***RP-HPLC*** (Phenomenex Luna C18, λ = 264 nm): t_R= 19.1 min.

time (min)	0	5	30	60
acetonitrile + 0.1% TFA (%)	30	30	90	100
water + 0.1% TFA (%)	70	70	10	0

$C_{43}H_{51}FN_2O_{19}$ (M = 918.865 g/mol). **ESI-MS** (positive), *m/z*: 941.13 ([M+Na]$^+$, calc.: 941.30), 1859.35 ([2 × M+Na]$^+$, calc.: 1859.60). **HR-ESI-MS** (positive), *m/z*: 941.2968 ([M+Na]$^+$, calc.: 941.2968).

2.11 Amino-4,7,10-trioxadodecanylamido-*N*-L-prolyl-L-alanyl-L-histidyl-L-glycyl-L-valyl-*O*-(2-acetamido-2-deoxy-3-*O*-[4-deoxy-4-fluoro-β-D-galactopyranosyl]-α-D-galactopyranosyl)-L-threonyl-L-seryl-L-alanyl-L-prolyl-L-aspartyl-L-threonyl-L-arginyl-L-porlyl-L-alanyl-L-prolyl-L-glycyl-L-seryl-L-threonyl-L-alanyl-L-proline (16)

The synthesis was carried out in an Applied Biosystems ABI 433A peptide synthesizer (standard program Fastmoc 0.1 mmol) using pre-loaded Fmoc-Pro-Trt-Tentagel S resin (417 mg, 0.10 mmol; loading: 0.24 mmol/g). For the coupling reactions, the amino acids Fmoc-Ala-OH, Fmoc-Arg(Pmc)-OH, Fmoc-Asp-OH, Fmoc-Gly-OH, Fmoc-His(Trt)-OH, Fmoc-Pro-OH, Fmoc-Ser(*t*Bu)-OH, Fmoc-Thr(*t*Bu)-OH, and Fmoc-Val-OH were employed. In every coupling cycle, the *N*-terminal Fmoc group was removed by treatment of the resin with a solution of piperidine (20%) in NMP for at least 3 × 2.5 min. The coupling of the amino acids (1.00 mmol or 10 eq. based on the loaded resin) was carried out with HBTU (1.00 mmol), HOBt (1.00 mmol) and DIPEA (2.00 mmol) in DMF (20−30 min vortex). After every coupling step, unreacted amino groups were capped by treatment with a mixture of Ac_2O (0.5 M), DIPEA (125 μM), and HOBt (1.50 μM) in NMP (10 min vortex).
Coupling of the protected T building block **11** (155 mg, 0.17 mmol, 1.69 eq. based on the loaded resin) was performed using HATU (1.2 eq. with respect to **11**), HOAt (1.2 eq.) and NMM (2.4 eq.) for activation (8 h vortex). After coupling of the remaining five amino acids by the standard procedure, the triethylene glycol spacer (1.00 mmol, 10 eq. based on the loaded resin) was coupled using HBTU (1.00 mmol), HOBt (1.00 mmol) and DIPEA (2.00 mmol) in DMF (20−30 min vortex) and the *N*-terminal Fmoc group was removed by piperidine (20%) in NMP. The peptide was detached from resin with simultaneous removal of all side chain protecting groups by shaking with TFA (15 mL), TIS (0.8 mL) and H_2O (0.8 mL) for 2 h. The solution was filtered, the resin was washed with TFA (2 × 10 mL) and the combined solutions were concentrated in vacuo, H_2O was added (10 mL) and the crude glycopeptide was subjected to lyophilisation.

The obtained peptide was dissolved in 30 mL of MeOH (HPLC grade). A fresh solution of NaOMe in MeOH (0.5 g Na in 25 mL MeOH (HPLC grade)) was added dropwise until pH 9.0 was reached. The reaction mixture was stirred over night and neutralized with a few drops of 1M HOAc. The solvent was removed in vacuo, H_2O was added (10 mL) and the residue was subjected to lyophilisation. The crude product was purified by preparative HPLC.

Yield: 33 mg (1.4 µmol, 13%) colorless lyophilisate. $[\alpha]_D^{23}$ = (1.00, H_2O) = -103.0. ***RP-HPLC*** (Phenomenex Luna C18, λ = 214 nm): t_R= 8.3 min.

time (min)	0	1	40	60
acetonitrile + 0.1% TFA (%)	5	5	70	100
water + 0.1% TFA(%)	95	95	30	0

$C_{103}H_{166}FN_{27}O_{41}$(M= 2457.575 g/mol). **^{1}H-NMR** (400 MHz, D_2O, COSY, HMQC), δ [ppm] = 8.50 (d, 1H, $J_{H\varepsilon,H\delta}$ = 1.31 Hz, H_ε), 7.19 (s, 1H, H_δ), 4.82 (d, 1H, $J_{H1,H2}$ = 3.80 Hz, H-1), 4.65–4.00 (m, 23H, H-4' {4.69}, D_α {4.62}, H_α {4.57}, R_α {4.52}, $T_{T*\alpha}$ {4.52}, $A_{3\alpha}$ {4.49}, $A_{2\alpha}$ {4.43}, H-1' {4.41}, $S_{2\alpha}$ {4.38}, $A_{4\alpha}$ {4.36}, $S_{1\alpha}$ {4.33}, $P_{1\text{-}5\alpha}$ {4.35, 4.32, 4.28, 4.23, 4.22}, $T_{2\alpha}$ {4.24}, $T_{1\alpha}$ {4.21}, V_α {4.19}, $T_{T*\beta}$ {4.19}, $A_{1\alpha}$ {4.12}, $T_{1\beta}$ {4.10}, H-2 {4.10}), 3.98–3.45 (m, 38H, $T_{2\beta}$ {3.98}, H-3 {3.92}, $G_{1\alpha}$ {3.92}, H-5 {3.92}, $G_{2\alpha}$ {3.84}, $S_{2\beta}$ {3.77}, H-6a,b {3.69}, $S_{1\beta}$ {3.69}, H-6a,b' {3.67}, H-5` {3.64}, $CH_2$-spacer {3.64}, H-3` {3.60, d, $J_{H3',F}$ = 17.42 Hz}, 4 × CH_2O-spacer {3.59–3.49}, H-4 {3.59}, $P_{1\text{-}5\delta}$ {3.68, 3.54, 3.48, 3.45}), 3.45–3.35 (m, 1H, H-2`), 3.18 (dd, 1H, $J_{H\beta a,H\beta b}$ = 15.45 Hz, $J_{H\beta a,H\alpha}$ = 5.50 Hz, $H_{\beta a}$), 3.13–3.01 (m, 7H, CH_2-spacer {3.11}, $H_{\beta b}$ {3.06}, R_δ {3.08}), 2.91–2.71 (m, 2H, $D_{\beta a}$, $D_{\beta b}$), 2.70–2.47 (m, 2H, CH_2-spacer), 2.29–2.06 (m, 6H, $P_{1\text{-}3\beta}$ {2.22, 2.18, 2.15}), 2.04–1.66 (m, 20H, $P_{1\text{-}5\gamma}$ {1.98–1.82}, V_β {1.96}, $P_{4\text{-}5\beta}$ {1.91, 1.79}, AcNH (s, 1.89}, $R_{\beta a}$ {1.73}), 1.68 – 1.48 (m, 3H, $R_{\beta b}$ {1.63}, Rγ {1.53}), 1.30–1.16 (m, 12H, $A_{2\beta}$, {1.29}, $A_{3\beta}$ {1.26}, $A_{4\beta}$ {1.22}, {1.20, d, $J_{A\beta,A\alpha}$ = 7.38 Hz, $A_{1\beta}$}), 1.14 (d, 3H, $J_{T*\gamma,T\beta}$ = 6.17 Hz, $T_{T*\gamma}$), 1.10–1.02 (m, 6H, $T_{2\gamma}$, $T_{1\gamma}$), 0.84 (t, 6H, $J_{V\gamma,V\beta}$ = 6.94 Hz, Vγ). **^{13}C-NMR** (100.6 MHz, D_2O, DEPT, HMQC), δ [ppm] = 175.78, 174.89, 174.79, 174.38, 173.92, 173.70, 173.61, 173.45, 173.09, 172.65, 172.61, 172.46, 172.36, 172.06, 171.98, 171.93, 171.54, 171.52, 171.32, 171.20, 171.11, 170.89, 170.86 (C=O, C=O(acetyl)), 156.66 (C=NH), 133.55 ($H_{C\gamma}$), 128.20 ($H_{C\varepsilon}$), 117.13 ($H_{C\delta}$), 104.31 (C-1'), 99.05 (C-1), 89.41 (d, $J_{C\text{-}4',F}$ = 176.24 Hz, C-4`), 77.20 (C-3), 76.92 ($T_{T*\beta}$), 73.50 (d, $J_{C\text{-}5',F}$ = 18.01 Hz, C-5`), 71.20 (d, $J_{C\text{-}3',F}$ = 17.42 Hz, C-3`), 70.97 (C-5), 70.48 (C-2`), 69.56, 69.49, 69.44, 69.41 (CH_2-spacer), 68.82 (C-4), 66.99 ($T_{1\beta}$), 66.90 ($T_{2\beta}$), 66.24 (CH_2-spacer), 61.14, 61.11 ($S_{1\beta}$, $S_{2\beta}$), 60.76, 59.99, 59.79, 59.33 ($P_{1\text{-}5\alpha}$), 60.76 (C-6), 60.02 (d, $J_{C\text{-}6',F}$ = 5.12 Hz, C-6'), 58.83 (V_α), 58.88 ($T_{1\alpha}$), 58.75 ($T_{2\alpha}$), 56.93 ($T_{T*\alpha}$), 55.84 ($S_{1\alpha}$), 55.53 ($S_{2\alpha}$), 52.27 (H_α), 51.08 (R_α), 50.06 (D_α), 49.59 (C-2), 48.66 ($A_{1\alpha}$), 47.67 ($A_{2\alpha}$), 47.59 ($A_{3\alpha}$), 47.38 ($A_{4\alpha}$), 47.73, 47.67, 47.59, 47.38 ($P_{1\text{-}5\delta}$), 42.37 ($G_{2\alpha}$), 42.31 ($G_{1\alpha}$), 40.48 (R_δ), 39.30 (CH_2-spacer), 34.90 (D_β), 34.00 (CH_2-spacer), 30.23 (V_β), 29.62, 29.31, 29.25, 29.17, 28.72 ($P_{1\text{-}5\beta}$), 27.44 (R_β), 26.18 (H_β), 24.70, 24.66, 24.60, 24.52, 24.31 ($P_{1\text{-}5\gamma}$), 23.97 (R_γ), 22.28

(CH_3-AcNH), 18.81 ($T_{1\gamma}$), 18.73 ($T_{2\gamma}$), 18.47 ($V_{\gamma a}$), 18.34 ($T_{T*\gamma}$), 17.80 ($V_{\gamma b}$), 16.29, 15.19, 15.07 ($A_{1\text{-}4\beta}$). **^{19}F-NMR** (376.5 MHz, $CDCl_3$), δ [ppm] = -217.21 (ddd, $J_{F4,4'\text{-}H}$ = 50.84 Hz, $J_{F4,3'\text{-}H}$ = 31.63 Hz, $J_{F4,3'\text{-}H}$ = 29.62 Hz, 4-F). **ESI-MS** (positive), *m/z*: 1229.15 ($[M+2H]^{2+}$, calc.: 1229.09); 819.77 ($[M+3H]^{3+}$, calc.: 819.72). **HR-ESI-MS** (positive), *m/z*: 1229.0951 ($[M+2H]^{2+}$, calc.: 1229.0937). **MALDI-TOF-MS** (dhb, positive) m/z: 2458.45 ($[M+H]^{+}$, calc.: 2458.57).

2.12 1-(Amino-4,7,10-trioxadodecanylamido-*N*-L-prolyl-L-alanyl-L-histidyl-L-glycyl-L-valyl-*O*-(2-acetamido-2-deoxy-3-*O*-[4-deoxy-4-fluoro-β-D-galactopyranosyl]-α-D-galactopyranosyl)-L-threonyl-L-seryl-L-alanyl-L-prolyl-L-aspartyl-L-threonyl-L-arginyl-L-porlyl-L-alanyl-L-prolyl-L-glycyl-L-seryl-L-threonyl-L-alanyl-L-prolyl)-2-ethoxycyclobutene-3,4-dione (17)

To a solution of 23.0 mg (9.36 μmol) of the glycopeptide **16** in a mixture of 5 mL ethanol and water (1:1) 1.45 μl (9.83 μmol) 3,4-diethoxy-3-cyclobutene-1,3-dione was added. Then 15 μl of sat. aqueous $NaHCO_3$ was added until pH 8.0 was reached and the mixture was stirred at room temperature for 3 h. The reaction mixture was neutralized with diluted acetic acid and subjected to lyophilisation. The crude product was purified by preparative RP-HPLC.

Yield: 6.0 mg (2.32 μmol, 25%) colorless lyophilisate. $[\alpha]_D^{23}$ = (0.60, H_2O) = -71.0. ***RP-HPLC*** (Phenomenex Luna C18, λ = 214 nm): t_R = 21.3 min.

time (min)	0	5	40	60
acetonitrile + 0.1% TFA (%)	5	5	25	70
water + 0.1% TFA (%)	95	95	75	30

$C_{109}H_{170}FN_{27}O_{44}$(M= 2581.669 g/mol). **^{1}H-NMR** (400 MHz, D_2O, COSY, HMQC), δ [ppm] = 8.51 (d, 1H, $J_{H\varepsilon,H\delta}$ = 1.35 Hz, H_ε), 7.20 (d, 1H, $J_{H\delta,H\varepsilon}$ = 1.08 Hz, H_δ), 4.83 (d, 1H, $J_{H1,H2}$ = 4.00 Hz, H-1), 4.70 - 4.00 (m, 25H, H-4' {4.70}, CH_2O-squarate {4.62}, D_α {4.59}, H_α {4.58}, R_α {4.53}, $T_{T*\alpha}$ {4.53}, $A_{3\alpha}$ {4.49}, H-1' {4.42}, $A_{2\alpha}$ {4.40}, $S_{2\alpha}$ {4.39}, $A_{4\alpha}$ {4.36}, $S_{1\alpha}$ {4.36}, $P_{1\text{-}5\alpha}$ {4.35, 4.31, 4.26, 4.24, 4.22}, $T_{1\alpha}$ {4.22}, $T_{T*\beta}$ {4.20}, V_α {4.19}, $T_{2\alpha}$ {4.14}, $A_{1\alpha}$ {4.12}, 2-H {4.12} $T_{1\beta}$ {4.09}), 3.98–3.48 (m, 42H, $T_{2\beta}$ {3.98}, $G_{1\alpha}$ {3.96}, H-5 {3.93}, H-3 {3.92}, $G_{2\alpha}$ {3.83}, $S_{2\beta}$ {3.77}, $S_{1\beta}$ {3.71}, H-6a,b' {3.68}, CH_2-spacer {3.68}, CH_2-spacer {3.65}, H-6a,b {3.62}, H-4 {3.61}, H-5` {3.60}, H-3` {3.60}, 4 × CH_2O-spacer {3.59–3.50}, $P_{1\text{-}5\delta}$ {3.76, 3.71, 3.57, 3.51}, 3.57 {CH_2-spacer}), 3.48–3.39 (m, 1H, H-2`),

3.25–3.15 (m, 1H, $H_{\beta a}$), 3.13–3.01 (m, 3H, R_{δ} {3.10}, $H_{\beta b}$ {3.09}), 2.91–2.68 (m, 2H, $D_{\beta a}$, $D_{\beta b}$), 2.68–2.49 (m, 2H, CH_2-spacer), 2.26–2.07 (m, 5H, $P_{1\text{-}5\beta a}$ {2.27, 2.22, 2.18, 2.15, 2.08}), 2.02–1.68 (m, 20H, $P_{1\text{-}5\gamma}$ {1.97–1.85}, V_{β} {1.96}, $P_{1\text{-}5\beta b}$ {1.93, 1.91, 1.86 1.79, 1.74}, NHAc (s, 1.88}, $R_{\beta a}$ {1.70}), 1.67–1.48 (m, 3H, $R_{\beta b}$ {1.59}, Rγ {1.54}), 1.32 (t, 3H, $J_{CH3,CH2}$ = 6.98 Hz, H_3C-CH_2-squarate), 1.29–1.18 (m, 12H, $A_{2\beta}$, {1.29}, $A_{3\beta}$ {1.27}, $A_{4\beta}$ {1.22}, {1.21, d, $J_{A\beta,A\alpha}$ = 7.22 Hz, $A_{1\beta}$}), 1.15 (d, 3H, $J_{T*\gamma,T\beta}$ = 6.32 Hz, $T_{T*\gamma}$), 1.11–1.03 (m, 6H, $T_{2\gamma}$, $T_{1\gamma}$), 0.84 (t, 6H, $J_{V\gamma,V\beta}$ = 6.83 Hz, Vγ). **^{13}C-NMR** (100.6 MHz, D_2O, DEPT, HMQC), δ [ppm] = 175.78, 174.89, 174.79, 174.38, 173.92, 173.70, 173.61, 173.45, 173.09, 172.65, 172.61, 172.46 172.36, 172.06, 171.98, 171.93, 171.54, 171.52, 171.32, 171.20, 171.11, 170.89, 170.86 (C=O, C=O(acetyl)), 156.66 (C=NH), 133.57 ($H_{C\gamma}$), 128.20 ($H_{C\varepsilon}$), 116.74 ($H_{C\delta}$), 104.21 (C-1’), 98.85 (C-1), 89.30 (d, $J_{C\text{-}4',F}$ = 174.58 Hz, C-4`), 77.25 (C-3), 76.88 ($T_{T*\beta}$), 73.57 (C-5`), 70.95 (C-5), 70.89 (C-3`), 70.62 ($CH_2$-squarate), 69.87 (C-2`), 69.64, 69.56, 69.44, 69.41 (CH_2-spacer), 68.69 (C-4), 68.66 ($T_{2\beta}$), 66.87 ($T_{1\beta}$), 66.10 (CH_2-spacer), 61.19 (C-6), 61.15, 61.11 ($S_{1\beta}$, $S_{2\beta}$), 60.79, 60.29, 60.08, 59.28 ($P_{1\text{-}5\alpha}$), 59.81 (C-6’), 58.96 ($T_{1\alpha}$), 58.94 (V_{α}), 58.75 ($T_{2\alpha}$), 56.87 ($T_{T*\alpha}$), 55.48 ($S_{1\alpha}$), 55.05 ($S_{2\alpha}$), 52.30 (H_{α}), 51.07 (R_{α}), 50.07 (D_{α}), 49.47 (C-2), 48.41 ($A_{1\alpha}$), 47.72 ($A_{2\alpha}$), 47.61 ($A_{3\alpha}$), 47.52 ($A_{4\alpha}$), 47.72, 47.67, 47.56, 47.39 ($P_{1\text{-}5\delta}$), 43.83 (CH_2-spacer), 42.25 ($G_{2\alpha}$), 42.21 ($G_{1\alpha}$), 40.44 (R_{δ}), 35.37 (D_{β}), 33.86 (CH_2-spacer), 30.23 (V_{β}), 29.36, 29.27, 29.18, 29.09, 28.77 ($P_{1\text{-}5\beta}$), 27.51 (R_{β}), 26.31 (H_{β}), 24.70, 24.68, 24.57, 24.51, 24.33 ($P_{1\text{-}5\gamma}$), 23.92 (R_{γ}), 22.28 (CH_3(NHAc)), 18.77 ($T_{1\gamma}$), 18.56 ($T_{2\gamma}$), 18.40 ($T_{T*\gamma}$), 18.36 ($V_{\gamma a}$), 17.73 ($V_{\gamma b}$), 16.23, 15.22, 15.11 ($A_{1\text{-}4\beta}$), 15.00 (H_3C-CH_2-squarate). **^{19}F-NMR** (376.5 MHz, $CDCl_3$), δ [ppm] = -217.2 (ddd, $J_{F4,4'\text{-}H}$ = 50.87 Hz, $J_{F4,3'\text{-}H}$ = 30.78 Hz, $J_{F4,3'\text{-}H}$ = 29.86 Hz, 4-F). **ESI-MS** (positive), *m/z*: 1291.09 ($[M+2H]^{2+}$, calc.: 1291.10); 868.39 ($[M+2H+Na]^{3+}$, calc.: 868.39). **HR-ESI-MS** (positive), *m/z*: 1291.1011 ($[M+2H]^{2+}$, calc.: 1291.1018). **MALDI-TOF-MS** (dhb, positive) m/z: 2583.44 ($[M+H]^{+}$, calc.: 2582.67).

2.13 BSA-conjugate (18a)

To a solution of 130 mg Na_2HPO_4 in 2 mL of water (pH 9.5) were added 2.0 mg (0.031 μmol) BSA and 2.0 mg (0.78 μmol) of the glycopeptide-squarate **17**. The mixture was stirred for 5 d at room temperature and was then subjected to dialysis (membrane 30 kDa). The crude product was filtrated with deionized water (3 × 50 mL) and subjected to lyophilisation to yield 5.6 mg (0.083 μmol) of the BSA conjugate as a colorless foam. **MALDI-TOF-MS**

(dhb, positive) m/z: 85290.99. An average of 7.3 molecules **17** per BSA could be assigned by MALDI-TOF-MS.

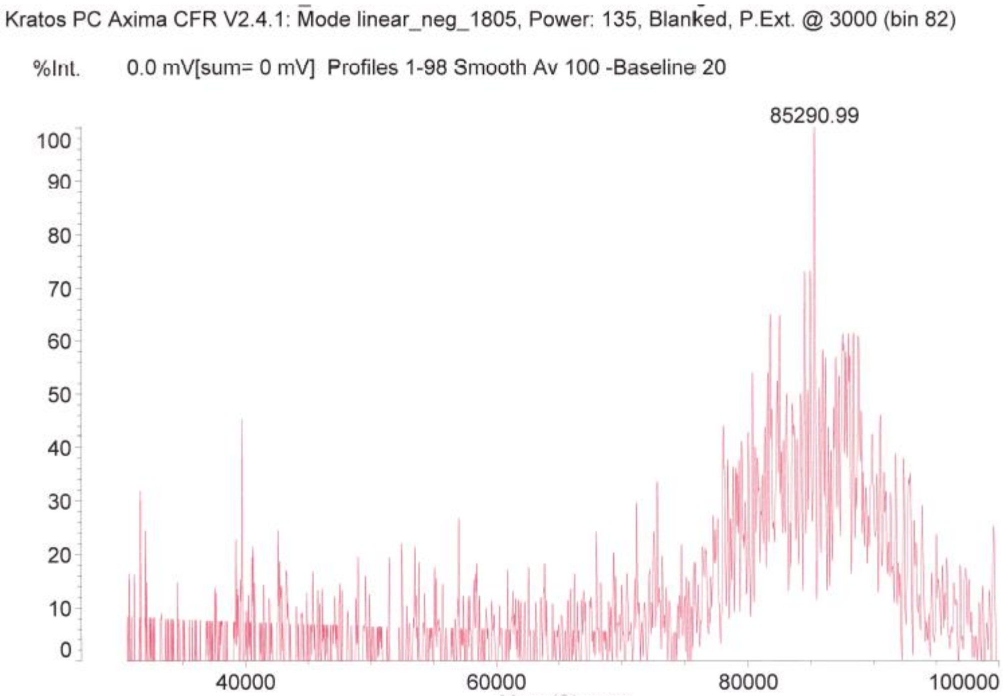

2.14 Tetanus-Toxoid-conjugate (18b)

To a solution of 130 mg Na_2HPO_4 in 1 mL of water (pH 9.5) were added 2.0 mg (0.014 μmol) Tetanus-Toxoid and 2.0 mg (0.78 μmol) of the glycopeptide-squarate **17**. The mixture was stirred for 5 d at room temperature and was then subjected to dialysis (membrane 30 kDa). The crude product was filtrated with deionized water (3 × 50 mL) and subjected to lyophilisation to yield 6.2 mg (0.041 μmol) of the TTox-conjugate as a colorless fluffy solid.

3 Enzymatic degradation

3.1 *N*-(9*H*-Fluoren-9-yl)-methoxycarbonyl-*O*-(2-acetamido-2-deoxy-4,6-*O*-benzylidene-3-*O*-[2,3,4,6-tetra-*O*-acetyl-β-D-pyranosyl]-α-D-galactopyranosyl)-L-threonine *tert*-butyl ester (19)

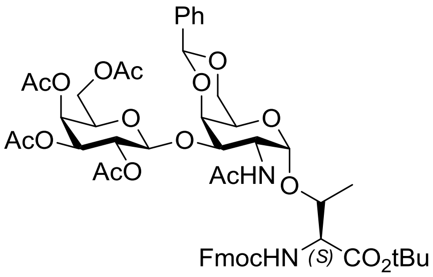

A suspension of 443 mg (0.64 mmol) Fmoc-Thr(α-4,6-Bzn-GalNAc)-*Ot*Bu **7a**, 33 mg (1.29 mmol) mercury(II) cyanide and freshly activated molecular sieves (4Å, 400 mg) in 10 mL abs. Dichloromethane and abs. Nitromethane (2:3) was stirred for 1 h. Then, 529 mg (1.26 mmol) 2,3,4,6-Tetra-*O*-acetyl-α-D-galactosyl bromide, dissolved in 10 mL abs. Dichloromethane and abs. Nitromethane (2:3), were added. The mixture was stirred for 2 d under an argon atmosphere at room temperature, before it was filtered through *Hyflo* into 80 mL sat. aq. $NaHCO_3$. The aqueous phase was extracted with 80 mL dichloromethane and the combined organic layers were washed successively with sat. aq. $NaHCO_3$ (40 mL) and sat. aq. NaCl (40 mL). The organic layer was dried over $MgSO_4$, filtered and the solvent was removed under reduced pressure. The crude product was purified by flash chromatography on silica (^{c}Hex/EtOAc, 1:1).

Yield: 425 mg (0.42 mmol, 65%) colorless amorphous solid. $\mathbf{R}_f$ = 0.27 (^{c}Hex/EtOAc, 1:1). $C_{53}H_{64}N_2O_{18}$ (M=1019.051 g/mol). **^{1}H-NMR** (300 MHz, $CDCl_3$), δ [ppm] = 7.78 (d, 2H, $J_{H4,H3} = J_{H5,H6}$ = 7.42 Hz, H-4-, H-5-Fmoc), 7.63 (d, 2H, $J_{H1,H2} = J_{H8,H7}$ = 6.65 Hz, H-1-, H-8-Fmoc), 7.56–7.48 (m, 2H, H-3-, H-6-Fmoc), 7.47–7.28 (m, 7H, H-2-, H-7-Fmoc, H_{Ar}), 5.96 (d, 1H, $J_{NH,H2}$ = 9.21 Hz, NH-urethane), 5.89 (d, 1H, $J_{NH,T\alpha}$ = 9.62 Hz, NH-Ac), 5.56 (s, 1H, C*H*Ph), 5.39 (d, 1H, $J_{H4',H3'}$ = 2.75 Hz, H-4’), 5.22–5.10 (m, 1H, H-2’), 5.00–4.89 (m, 2H, H-3’ {4.99}, H-1 {4.94}), 4.82–4.65 (m, 2H, H-1’ {4.74}, H-2 {4.70}), 4.62–4.39 (m, 3H, T^α {4.22}, CH_2-Fmoc {4.54}), 4.30–4.18 (m, 5H, CH-Fmoc, T^β {4.27}, H-4 {4.26}, H-6a {4.22}, H-6a’ {4.24}), 4.15–4.03 (m, 2H, H-6b {4.07}, H-6b’ {4.12}), 3.92–3.84 (m, 2H, H-3 {3.88}, H-5), 3.73–3.62 (m, 1H, H-5’), 2.17 (s, 3 H, -OAc), 2.04 (s, 3 H, CH_3(NHAc)), 2.00, 1.97 (2 × s, 6H, -OAc), 1.47 (s, 9H, CH_3(*t*Bu)), 1.27 (d, 3H, $J_{T\gamma,T\beta}$ = 6.23 Hz, T^γ).

3.2 *N*-(9*H*-Fluoren-9-yl)-methoxycarbonyl-*O*-(2-acetamido-2-deoxy-3-*O*-[2,3,4,6-tetra-*O*-acetyl-β-D-pyranosyl]-α-D-galactopyranosyl)-L-threonine *tert*-butyl ester (20)

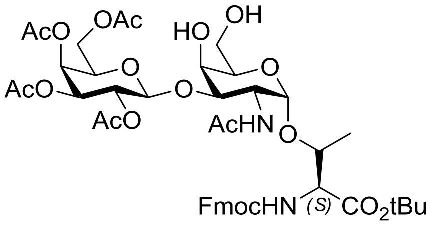

To a solution of disaccharide **19** (538 mg, 0.53 mmol) in a mixture of CH_2Cl_2 and MeOH (4:1, 50 mL) were added $NaHSO_4$-SiO_2 (500 mg) and the suspension was stirred for 18 h at room temperature. The catalyst was filtered off and the filtrate was washed with sat. aq. $NaHCO_3$ (3 × 50 mL) and brine (50 mL), dried over $MgSO_4$, filtered and concentrated under reduced pressure. The crude product was purified by flash chromatography on silica (EtOAc). **Yield:** 383 mg (0.41 mmol, 78%) colorless amorphous solid. **R*f*** = 0.31 (EtOAc). $C_{45}H_{58}N_2O_{19}$(M= 930.944 g/mol). **^{1}H-NMR** (300 MHz, $CDCl_3$), δ [ppm] = 7.91 (d, 2H, $J_{H4,H3} = J_{H5,H6}$ = 8.66 Hz, H-4, H-5-Fmoc), 7.75 (d, 2H, $J_{H1,H2} = J_{H8,H7}$ = 7.42 Hz, H-1-, H-8-Fmoc), 7.53–7.46 (m, 2H, NH-urethane {7.51, d, $J_{NH,Ta}$ = 8.87 Hz}, NH-Ac {7.48, d, $J_{NH,H2}$ = 9.61 Hz}), 7.45–7.38 (m, 2H, H-3-, H-6-Fmoc), 7.36–7.28 (m, 2H, H-2-, H-7-Fmoc), 5.28 (d, 1H, $J_{H3',H4'}$ = 3.42 Hz, H-3'), 5.05–4.94 (m, 2H, H-4' {5.02, d, $J_{H4',H3'}$ = 3.43 Hz}, H-2' {4.99}), 4.74 (d, 1H, $J_{H1',H2'}$ = 7.67 Hz, H-1'), 4.58 (d, 1H, $J_{H1,H2}$ = 4.15 Hz, H-1), 4.54 – 4.40 (m, 2H, CH_2-Fmoc), 4.32 (t, 1H, $J_{CH,CH2}$ = 6.7 Hz, CH-Fmoc), 4.28–4.19 (m, 2H, H-2 {4.24}, T^{β} {4.22}), 4.18–4.11 (m, 2H, H-6a {4.15}, H-5 {4.15}), 4.07 (dd, 1H, $J_{T\alpha,H2}$ = 9.96 Hz, $J_{T\alpha,T\beta}$ = 1.7 Hz, T^{α}), 4.02–3.94 (m, 1H, H-6b), 3.91–3.88 (m, 1H, H-4), 3.64 (t, 1H, $J_{H5,H6a/b}$ = 6.72 Hz, H-5), 3.56 (dd, 1H, $J_{H3,H2}$ = 11.27 Hz, $J_{H3,H4}$ = 2.7 Hz, H-3), 3.50–3.44 (m, 2H, H-6'a/b), 2.12, 2.07, 2.00, 1.99, 1.91 (4 × s, 12H, -OAc), 1.84 (s, 3H, CH_3(NHAc)), 1.34 (s, 9H, CH_3(*t*Bu)), 1.14 (d, 3H, $J_{T\gamma,T\beta}$ = 6.44 Hz, T^{γ}).

3.3 *N*-(9*H*-Fluoren-9-yl)-methoxycarbonyl-*O*-(2-acetamido-2-deoxy-3-*O*-[β-D-galactopyranosyl]-α-D-galactopyranosyl)-L-threonine *tert*-butyl ester (13)

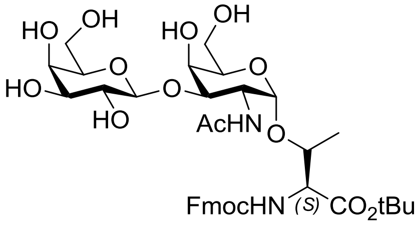

A solution of 200 mg (0.23 mmol) Fmoc-Thr(βAc$_4$-Gal-(1→3)-α-GalNAc)-O*t*Bu **20** in 20 mL methanol was treated drop wise with a freshly prepared NaOMe solution (0.5 g Na in 25 mL methanol) until a pH of 8.5 was reached. The mixture was stirred at room temperature overnight, neutralized with diluted acetic acid and the solvent was removed under reduced pressure. The crude product was purified by RP-HPLC.

Yield: 14 mg (19 µmol, 48%) colorless amorphous solid. $[\alpha]_D^{23}$ = (1.00, $CHCl_3$) = +49.3. ***RP-HPLC*** (Phenomenex Luna C18, l = 264 nm): t_R = 11.6 min.

time (min)	0	5	30	60
acetonitril (%)	30	30	90	100
water (%)	70	70	10	0

$C_{37}H_{50}N_2O_{15}$ (M = 762.797 g/mol). **^{1}H-NMR** (400 MHz, $CDCl_3$, COSY, HMQC) δ [ppm] = 7.67 (d, 2H, $J_{H4,H3}$ = $J_{H5,H6}$ = 7.51 Hz, H-4-, H-5-Fmoc), 7.58 (d, 1H, $J_{H1,H2}$ = 7.05 Hz, H-1-Fmoc), 7.45 (d, 1H, $J_{H8,H7}$ = 6.95 Hz, H-8-Fmoc), 7.31 (t, 2H, $J_{H3,H4}$ = $J_{H6,H5}$ = 7.06 Hz, H-3-, H-6-Fmoc), 7.28 – 7.26 (m, 2H, H-2-, H-7-Fmoc), 6.95 (bs, 1H, NH-Ac), 6.53, 6.40 (2bs, 1H, NH-urethane), 4.80, 4.71 (2s, 1H, H-1), 4.51–4.23 (m, 5H, H-1', H-2', H-2, CH_2-Fmoc), 4.23–4.01 (m, 3H, CH-Fmoc, T^α, T^β), 4.01–3.72 (m, 6H, H-3, H-4, H-5, H-6a/b, H-4'), 3.72–3.46 (m, 4H, H-3', H-5', H-6a/b'), 3.01 (bs, 6H, 6 × OH), 2.01 (s, 3H, -CH_3), 1.33 (s, 9H, 3 × CH_3(*t*Bu)), 1.20 (d, 3H, $J_{T\gamma,T\beta}$ = 6.89 Hz, T^γ). **^{13}C-NMR** (100.6 MHz, DEPT, HMQC, $CDCl_3$), δ [ppm] = 170.3 (C=O(NHAc)), 156.9 (C=O(urethane)), 143.9 (C-1a-, C-8a-Fmoc), 141.2 (C-4a-, C-5a-Fmoc), 127.7 (C-3-, C-6-Fmoc), 127.1 (C-2-, C-7-Fmoc), 125.2, 124.8 (C-1-, C-8-Fmoc), 119.9 (C-4-, C-5-Fmoc), 105.3 (C-1'), 99.9 (C-1), 82.7 (C*q*(*t*Bu)), 79.2 (C-3), 77.2 (T^β), 74.7 (C-5'), 73.1 (C-3'), 71.0 (C-5), 70.1 (C-4'), 68.9 (C-4), 68.4 (C-2'), 67.1 (CH_2-Fmoc), 61.4 (C-6), 60.8 (C-6'), 59.5 (T^α), 48.4 (C-2), 47.1 (CH-Fmoc), 28.0 (CH_3(*t*Bu)), 23.3 (CH_3(NHAc)), 18.9 (T^γ). **ESI-MS** (positive), *m/z*: 785.31 ($[M+Na]^+$, calc.:

785.31), 1547.64 ([2 × M+Na]$^+$, calc.: 1547.63), 2311.00 ([3 × M+Na]$^+$, calc.: 2310.96). **HR-ESI-MS** (positive), *m/z*: 785.3119 ([M+Na]$^+$, calc.: 785.3109).

3.4 *N*-(9*H*-Fluoren-9-yl)-methoxycarbonyl-*O*-(2-acetamido-2-deoxy-3-*O*-[β-D-4-deoxy-4-fluoro-galactopyranosyl]-α-D-galactopyranosyl)-L-threonine *tert*-butyl ester (12)

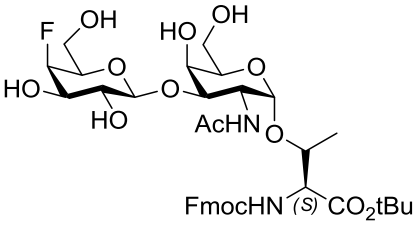

A solution of 200 mg (0.23 mmol) Fmoc-Thr(βAc$_3$-GalF-(1→3)-α-GalNAc)-O*t*Bu **10** in 20 mL methanol was treated drop wise with a freshly prepared NaOMe solution (0.5 g Na in 25 mL methanol) until a pH of 8.5 was reached. The mixture was stirred at room temperature overnight, neutralized with diluted acetic acid and the solvent was removed under reduced pressure. The crude product was purified by RP-HPLC.

Yield: 22.9 mg (0.03 mmol, 13%) colorless amorphous solid. $[\alpha]_D^{23}$ = (1.00, MeOH) = +42.5.

RP-HPLC (Phenomenex Luna C18, λ = 264 nm): t_R = 14.1 min.

time (min)	0	5	30	60
acetonitrile (%)	30	30	90	100
water (%)	70	70	10	0

$C_{37}H_{49}FN_2O_{14}$ (M = 764.788 g/mol). **^{1}H-NMR** (400 MHz, DMSO, COSY, HMQC) δ [ppm] = 7.91 (d, 2H, $J_{H4,H3}$ = $J_{H5,H6}$ = 7.71 Hz, H-4-, H-5-Fmoc), 7.74 (d, 2H, $J_{H1,H2}$ = $J_{H8,H7}$ = 6.79 Hz, H-1-, H-8-Fmoc), 7.61 (d, 1H, $J_{NH,T\alpha}$ = 9.83 Hz, NH-urethane), 7.47 (d, 1H, $J_{NH,H2}$ = 10.10 Hz, NH-Ac), 7.43 (t, 2H, $J_{H3,H4}$ = $J_{H6,H5}$ = 7.66 Hz, H-3-, H-6-Fmoc), 7.36–7.28 (m, 2H, H-2-, H-7-Fmoc), 5.27 (d, 1H, $J_{prim.-OH,H6}$ = 5.42 Hz, prim.-OH), 4.89 (dd, 1H, $J_{prim.-OH,H6}$ = $J_{OH,H6}$ = 5.33 Hz, prim.-OH), 4.69–4.49 (m, 5H, H-1, H-4', 3 × sek.-OH), 4.49–4.37 (m, 2H, CH_2-Fmoc), 4.37–4.17 (m, 4H, H-1', 2-H, CH-Fmoc, T^β), 4.08 (dd, 1H, $J_{HT^\alpha,NH}$ = 9.92 Hz, J_{HT^α,HT^β} = 1.51 Hz, T^α), 3.94–3.89 (m, 1H, H-4), 3.71–3.58 (m, 2H, H-3, H-5), 3.55–3.39 (m, 5H, H-5', H-6a/b', H-6a/b), 3.39–3.27 (m, 2H, H-2', H-3'), 1.82 (s, 3H, -CH_3), 1.35 (3 × s, 9H, -CH_3(*t*Bu)), 1.15 (d, 3H, $J_{T\gamma,T\beta}$ = 6.39 Hz, T^γ). **^{13}C-NMR** (100.6 MHz, DEPT, HMQC, DMSO), δ [ppm] = 169.5 (C=O(NHAc)), 157.3 (C=O(urethane)), 144.2 (C-1a-, C-8a-Fmoc),

141.3 (C-4a-, C-5a-Fmoc), 128.2 (C-3-, C-6-Fmoc), 127.5 (C-2-, C-7-Fmoc), 125.8, 125.7 (C-1-, C-8-Fmoc), 120.7 (C-4-, C-5-Fmoc), 105.0 (C-1'), 99.8 (C-1), 89.4 (d, $J_{C4',F}$ = 179.56 Hz, C-4'), 81.7 (C*q*(*t*Bu)), 78.1 (C-3), 74.5 (T^{β}), 73.8 (d, $J_{C5',F}$ = 18.36 Hz, C-5'), 72.2 (C-5), 72.0 (d, $J_{C3',F}$ = 17.60 Hz, C-3'), 71.1 (C-2'), 68.2 (C-4), 66.1 (CH_2-Fmoc), 61.2 (C-6), 59.8 (T^{α}), 59.6 (d, $J_{C6',F}$ = 4.84 Hz, C-6'), 47.8 (C-2), 47.2 (CH(Fmoc)), 28.1 (CH_3(*t*Bu)), 23.5 (CH_3(NHAc)), 19.6 (T^{γ}). **^{19}F-NMR** (376.5 MHz, $CDCl_3$), δ [ppm] = -216.6 (ddd, $J_{F,H4'}$ = 50.66 Hz, $J_{F,H3'}$ = 30.63 Hz, $J_{F,H5'}$ = 30.63 Hz). **ESI-MS** (positive), *m/z*: 787.30 ($[M+Na]^+$, calc.: 787.31), 1551.67 ($[2 \times M+Na]^+$, calc.: 1551.62). **HR-ESI-MS** (positive), *m/z*: 787.3071 ($[M+Na]^+$, calc.: 787.3066).

3.5 Enzymatic degradation assay

A stock solution of the buffer was prepared at first. Therefore 21.3 mg (0.11 mmol) 2-(*N*-morpholino)ethanesulfonic acid (MES-buffer) was dissolved in 10 mL millipore water (10 mM solution). The antigens (natural TF antigen: 3.81 mg, 5.00 mmol and 4'F-TF antigen: 3.82 mg,5.00 mmol, respectively) were dissolved in 1 mL freshly prepared buffer solution in a test tube. Then, 13.3 mg (10.0 mmol) dimethyl-β-cyclodextrin and 0.50 mg (0.08 μmol) BSA were added and the mixture was shaked thoroughly at room temperature until a clear solution was obtained. The pH of the solutions was kept at 4.5 all the time. Then 0.1 units of the β-galactosidase from bovine testes (0.11 mL, Suspension in 3.2 M $(NH_4)_2SO_4$-Puffer) was added and aliquots of 30 μL were taken periodically and subjected to HPLC analysis (RP-HPLC, Phenomenex Luna C18, λ = 264 nm) and finally to mass spectrometry.

RP-HPLC (Phenomenex Luna C18, λ = 264 nm):

time (min)	0	5	30	60
acetonitril (%)	30	30	90	100
water (%)	70	70	10	0

Data of Fig. 1: Data for enzymatic hydrolysis of the native TF antigen **13**.

time [min]	native TF antigen	Tn
0	100,000	0
30	78,295	21,705
60	66,739	33,261
120	50,764	49,236
180	39,048	60,952
240	27,962	72,038
300	19,518	80,482
360	12,142	87,858
420	7,167	92,833
960	0,000	100

Data for the enzymatic hydrolysis of the 4'F-TF antigen **12**.

time [min]	Ratio 4‘F-TF/Tn
0	1,000
30	1,000
60	1,000
120	1,000
240	1,000
420	1,000

4 Immunological evaluation

4.1 Immunization procedure

The vaccine adjuvant samples were prepared using 250 µL of a solution of the vaccine **18b** (0.25 µg/mL) in phosphate buffered saline (PBS) and either 250 µL of complete Freund's adjuvant CFA (DIFCO) or 250 µL incomplete Freund's adjuvant IFA (Sigma-Aldrich). The emulsions were transferred to syringes and stored on ice until administered.

Three female Balb/c mice at the age of 6-8 weeks, which were kept under sterile conditions, were immunized three times at intervals of three weeks with 40 µL of the above described emulsions (ca. 10 µg **18b**). The first immunization was administered subcutaneously with CFA, the following boost immunizations were administered intraperitoneally with IFA. Five days after the boost immunizations blood was collected from the tail vein and subsequently centrifuged twice at 10000 rpm for 10 min. After each centrifugation the supernatant was collected to obtain the serum antibodies.

4.2 Enzyme-linked immune sorbent assays

Determination of MUC1-specific serum antibodies:

96-well plates (Nunc MaxiSorp® flat-bottom) were incubated with 50 µL per well of a solution of MUC1 glycopeptide-BSA conjugate **18a** (2.5 µL/mL) in coating buffer (0.1 M Na_2HPO_4 in water, pH 9.3) at 37 °C. The coated plates were washed three times with 100 µL blocking buffer (1% BSA, 0.2% Tween-20 in PBS) and incubated for 30 min with 50 µL of blocking buffer at 37 °C in order to saturate free binding sites. The serum samples were applied in the first column of the 96-well plate with an initial dilution of 1:50 in blocking buffer and then serially diluted in a ratio of 1:1. After incubation for 1 h at 37 °C the plate was washed three times with 100 µL blocking buffer. Then, the samples were incubated with biotinylated sheep-a-mouse antibody (c = 0.48 µg/mL) for 1 h at 37 °C in 50 µL blocking buffer. Again, the samples were washed three times with 100 µL blocking buffer and subsequently incubated at 37 °C with streptavidin-horseradish peroxidase (c = 0.5 µg/mL) in 50 µL blocking buffer per well. After washing three times with 100 µL blocking buffer each well was treated with a solution of 1 mg/mL 2,2'-azino-bis(3-ethylbenzothiazoline-6-sulphonic acid) (ABTS) , 0.01% hydrogen peroxide in citrate buffer (40 mM citric acid, 60 nM $Na_2HPO_4 \times H_2O$, pH 4.4-4.5). After minutes of incubation at room temperature, the

optical density of each well at 410 nm was measured with a spectrophotometer (Tecan Reader, Genios).

Data of Fig. 2: ELISA binding studies of the antiserum induced by vaccine **18b** (coat: BSA conjugate **18a**).

dilution	*1/500*	*1/1,000*	*1/2,000*	*1/4,000*	*1/8,000*	*1/16,000*
mouse F1	1.6571	1.5965	1.5652	1.4698	1.4029	1.2699
mouse F2	1.9411	1.9138	1.8812	1.8116	1.7490	1.5503
mouse F3	1.5476	1.4913	1.4600	1.3050	1.1381	0.8931
(+)-control		1.5982	1.3693	1.0362	0.7664	0.6140
(-)-control						
dilution	*1/32,000*	*1/64,000*	*1/128,000*	*1/256,000*	*1/512,000*	*1/1,024,000*
mouse F1	1.0961	0.8812	0.6742	0.5222	0.4499	0.4411
mouse F2	1.2664	0.9544	0.7513	0.6054	0.5102	0.4858
mouse F3	0.6827	0.5628	0.4799	0.4260	0.4026	0.3866
(+)-control	0.5232					
(-)-control	0.4239	0.4290	0.4166	0.4169	0.4033	0.4065

(+)-control: MUC1-specific mouse serum antibodies induced by vaccination with a previously reported glycopeptide-TTox conjugate.[4]
(-)-control: ELISA performed on wells lacking the MUC1 glycopeptide-BSA coat.

Determination of TTox-specific serum antibodies:

ELISA binding studies were performed using the protocol described above and tetanus toxoid instead of the glycopeptide-BSA conjugate.

dilution	*1/500*	*1/1,000*	*1/2,000*	*1/4,000*	*1/8,000*	*1/16,000*	*1/32,000*
mouse F1	2.5654	2.5990	2.6522	2.6706	2.6638	2.6038	2.3519
mouse F2	2.2910	2.2970	2.4405	2.4188	2.3850	2.3292	2.1173
mouse F3	2.2389	2.1929	2.1602	2.1647	2.1257	1.9895	1.6461
(+)-control		2.1187	2.1175	2.1709	2.1025	1.8531	1.5304
(-)-control		0.0602	0.0608	0.0640	0.0672	0.0709	0.0655
dilution	*1/64,000*	*1/128,000*	*1/256,000*	*1/512,000*	*1/1,024,000*	*1/2,048,000*	
mouse F1	1.8796	1.4062	0.8734	0.5470	0.3178		
mouse F2	1.6999	1.1742	0.7542	0.4355	0.2630		
mouse F3	1.2455	0.7783	0.5563	0.3478	0.2344		
(+)-control	1.1358	0.7353	0.4401	0.2723	0.1706	0.1255	
(-)-control	0.0794	0.0801	0.0702	0.0655	0.0716	0.0730	

(+)-control: MUC1-specific mouse serum antibodies induced by vaccination with a previously reported glycopeptide-TTox conjugate.[5]
(-)-control: ELISA performed on wells lacking the TTox coat.

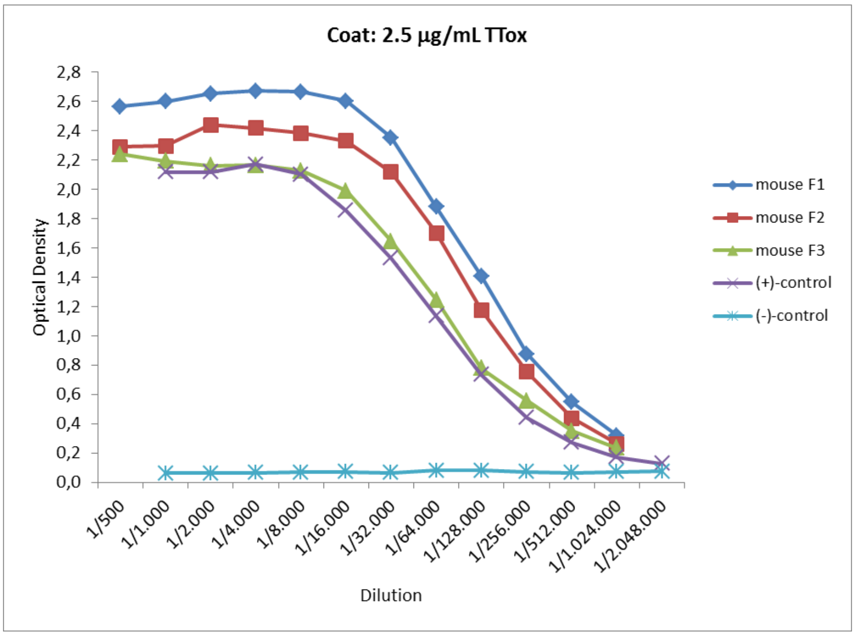

Determination of the isotypes of the antibodies induced by vaccine 18b

The isotypes of the elicited MUC1-specific antibodies were determined by ELISA using the protocol described above**.** The following secondary antibodies were used: biotinylated anti-mouse-IgG2a (BD Pharmingen, clone R19-15, *c* = 1 µg/mL), biotinylated anti-mouse-IgG2b (BD Pharmingen, clone G15-337, *c* = 0.5 µg/mL), biotinylated anti-mouse-IgG1 (BD Pharmingen, *c* = 1 µg/mL), biotinylated anti-mouse-IgA (eBioscience, clone 11-44-2, *c* = 1 µg/mL), biotinylated anti-mouse-IgD (eBioscience, clone B121-15F9, *c* = 1 µg/mL).

Data of Fig. 3: antiserum of mouse F2 (coat: BSA conjugate **18a**)

dilution	*1/2,000*	*1/4,000*	*1/8,000*	*1/16,000*
Ig G2a	0.6756	0.4138	0.2196	0.1384
Ig G2b	0.9384	0.5529	0.2959	0.2007
Ig G1	1.2567	1.2279	1.0274	0.7982
Ig A	0.0942	0.0777	0.0708	0.0711
Ig D	0.0687	0.0603	0.0667	0.0687
Ig M	0.0922	0.0807	0.0743	0.0677
(+)-control	1.2762	1.2610	1.1268	0.8813
(-)-control	0.0707	0.07024	0.0826	0.0774

(+)-control: anti-mouse (sheep) antibody (*c* = 0.48 µg/mL)[6]
(-)-control: pre-immunized mouse serum

4.3 Analysis of the antibody binding to MCF-7 cells

For the flowcytometric analysis of the serum antibody binding to the human breast cancer cell line MCF-7 10^5 cells per sample were transferred to a 96-well plate. The cells were washed with 100 µL PBS buffer and incubated with 50 µL serum (diluted 1:100 in PBS + 0.5% BSA) for 20 min at a temperature of 4 °C. The cells were washed twice with 100 µL PBS and again were incubated for 20 min at a temperature of 4 °C with a goat-α-mouse-IgG-Alexa Fluor 488 antibody (c = 2 µg/mL in PBS, Invitrogen A11029). The cells were washed again twice with 100 µL PBS, taken up in 100 µL PBS and pipetted into a FACS tube for analysis. For each sample 104 cells were analyzed on a BD Bioscience FACSVerse machine.

2nd Ab (αmouse IgG-FITC) **pos. control (αMUC1 1.30)[7]**

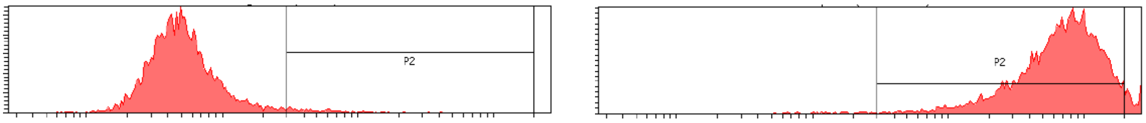

Mice Gr.F: TTox-MUC1-4´F-TF (5d after 1st boost)

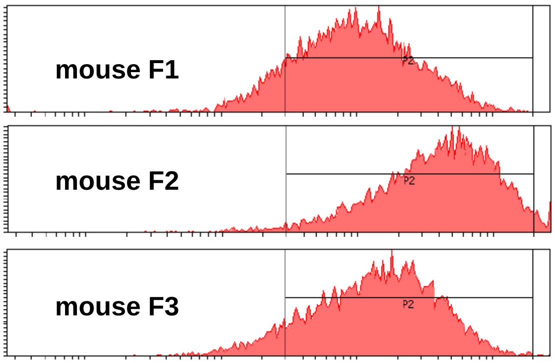

Mice Gr.F: TTox-MUC1-4´F-TF (5d after 2nd boost)

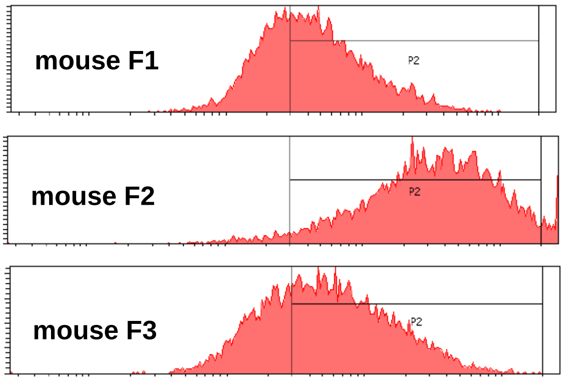

5 NMR-Spectra

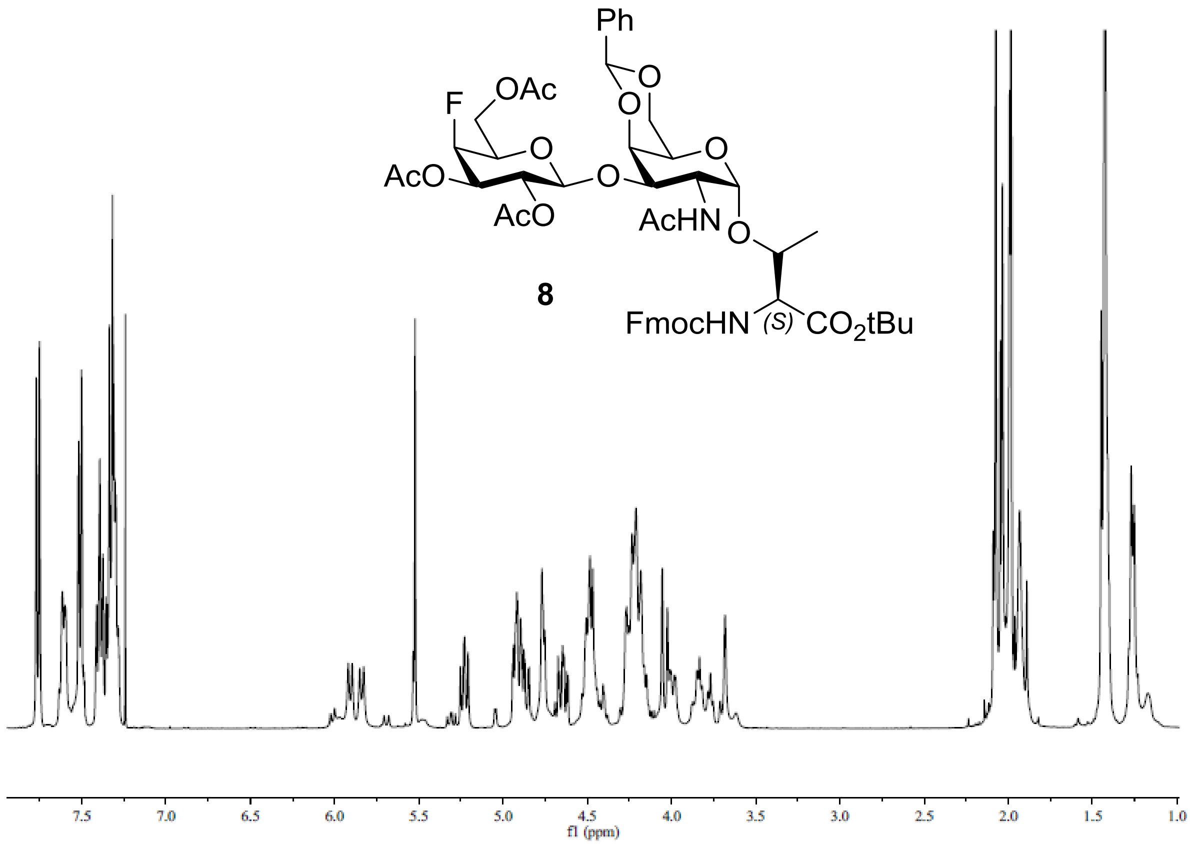

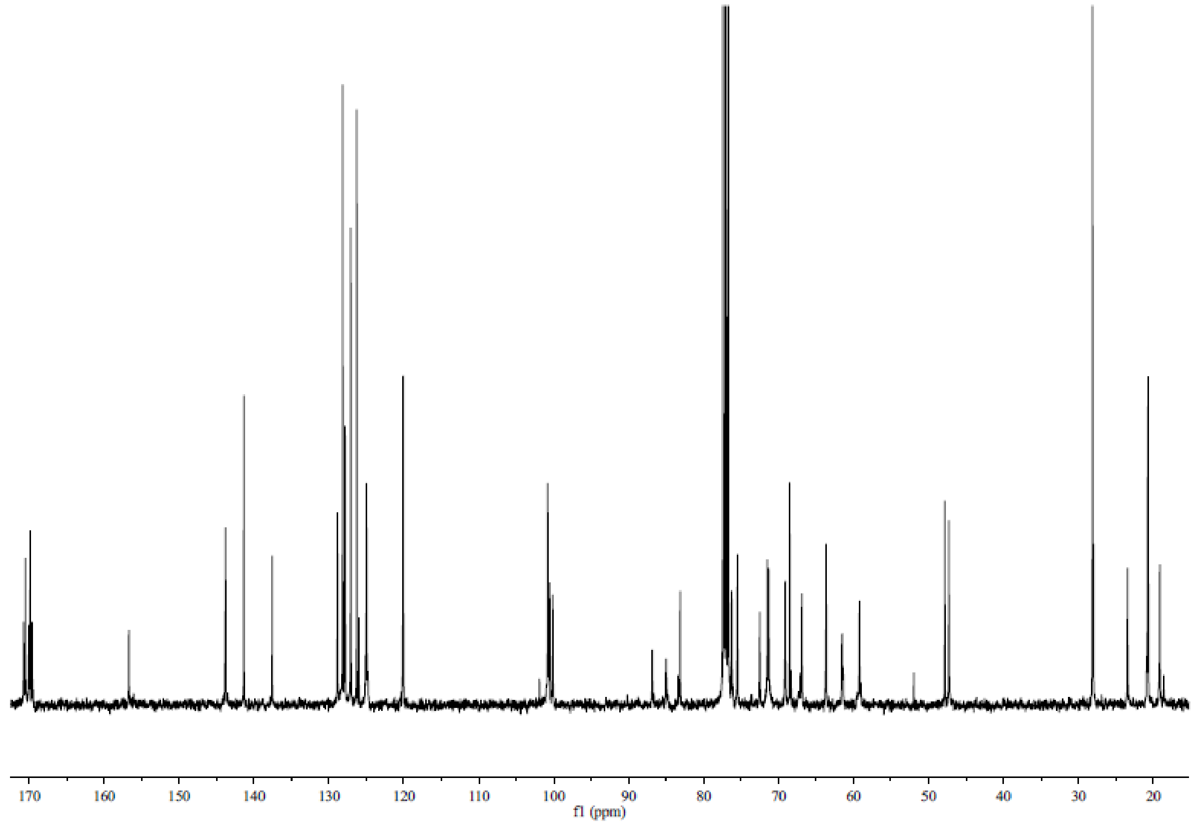

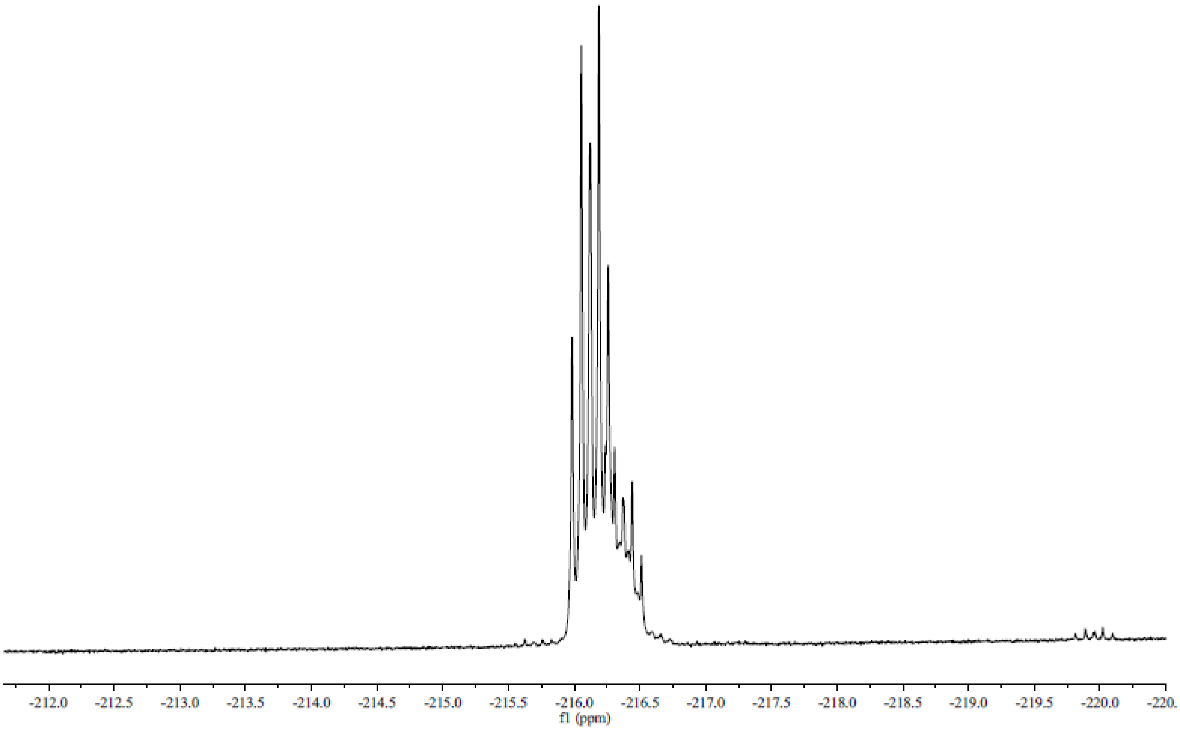

-212.0
-212.5
-213.0
-213.5
-214.0
-214.5
-215.0
-215.5
-216.0
-216.5
-217.0
-217.5
-218.0
-218.5
-219.0
-219.5
-220.0
-220.
f1 (ppm)

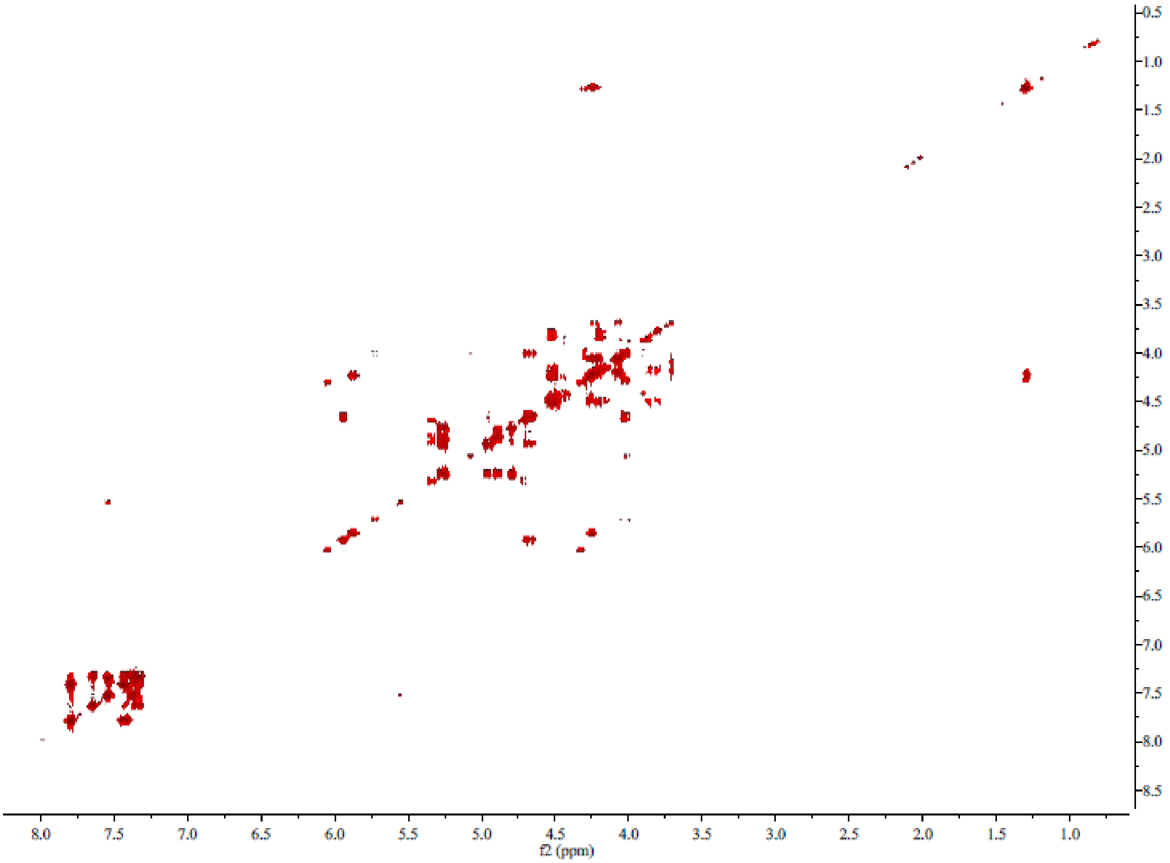

0.5
1.0
1.5
2.0
2.5
3.0
3.5
4.0
4.5
5.0
5.5
6.0
6.5
7.0
7.5
8.0
8.5
8.0
7.5
7.0
6.5
6.0
5.5
5.0
4.5
4.0
3.5
3.0
2.5
2.0
1.5
1.0
f2 (ppm)

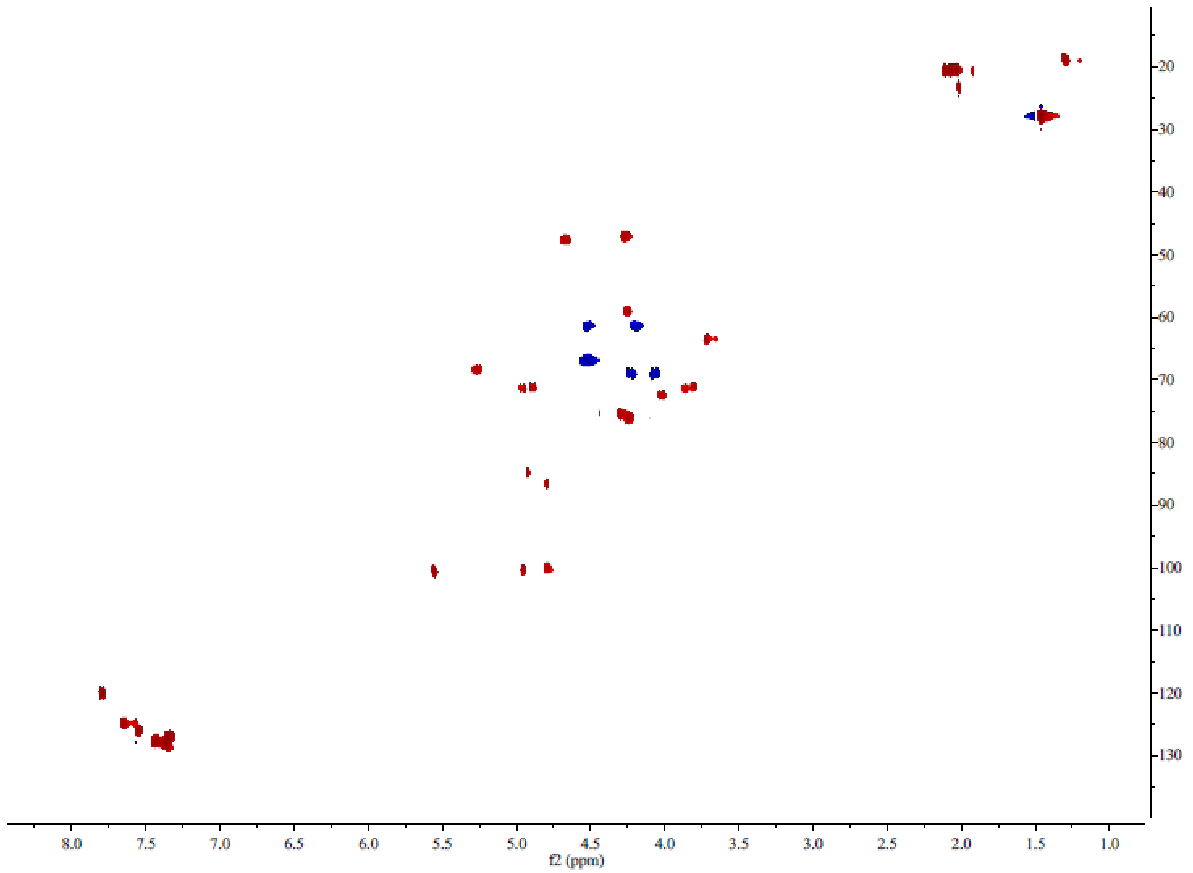
f2 (ppm)

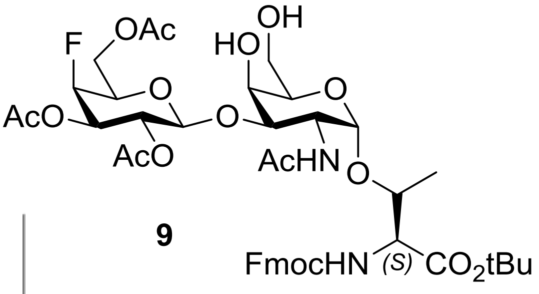
F
OAc
OH
HO
O
AcO
AcO
O
O
AcHN
O
FmocHN
(S)
CO_2tBu

9

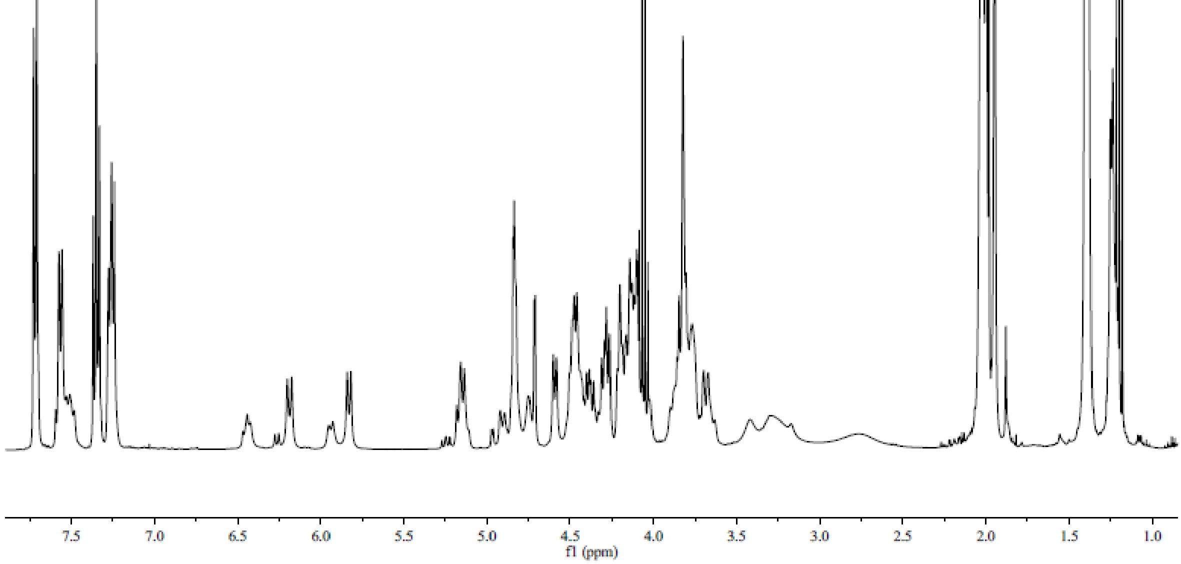
7.5
7.0
6.5
6.0
5.5
5.0
4.5
4.0
3.5
3.0
2.5
2.0
1.5
1.0
f1 (ppm)

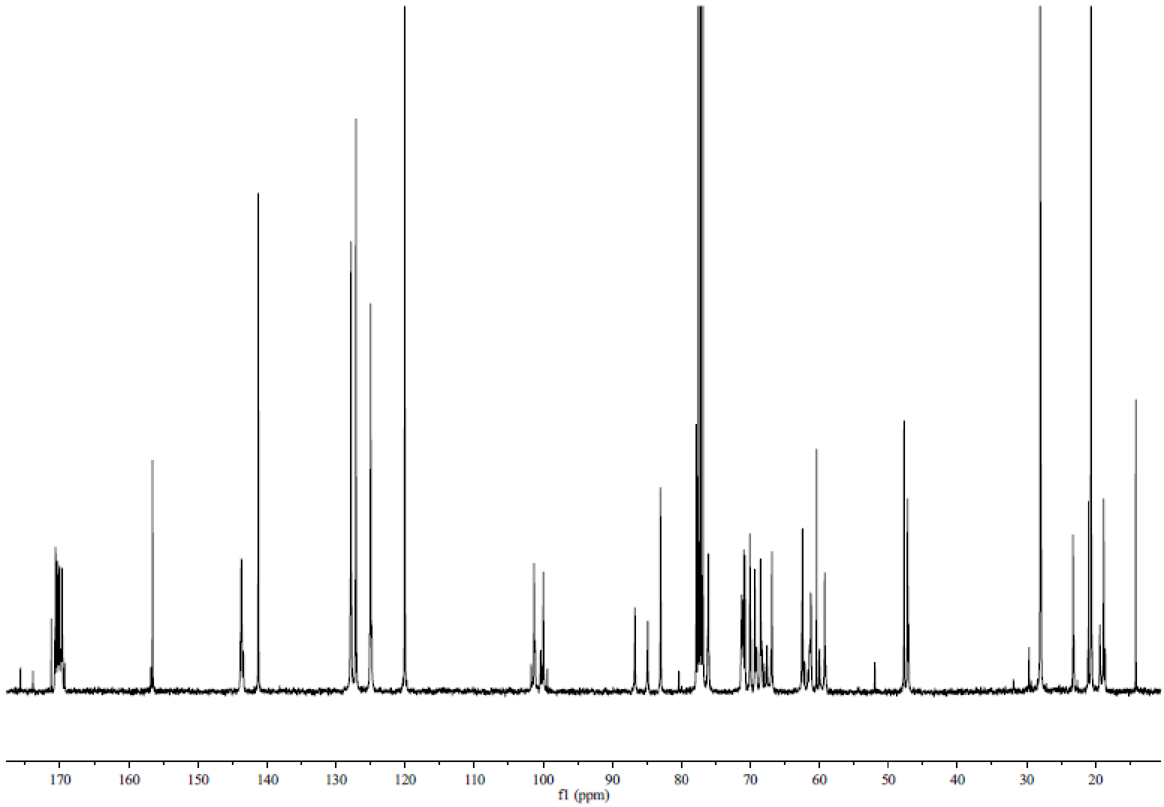
170
160
150
140
130
120
110
100
90
80
70
60
50
40
30
20
f1 (ppm)

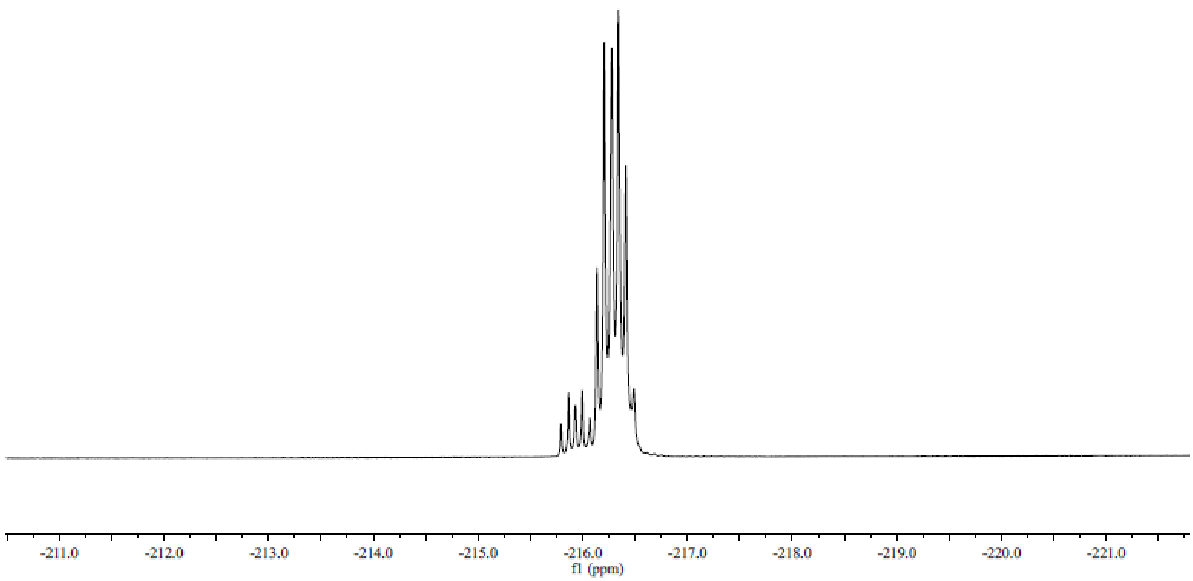

-211.0
-212.0
-213.0
-214.0
-215.0
-216.0
-217.0
-218.0
-219.0
-220.0
-221.0
f1 (ppm)

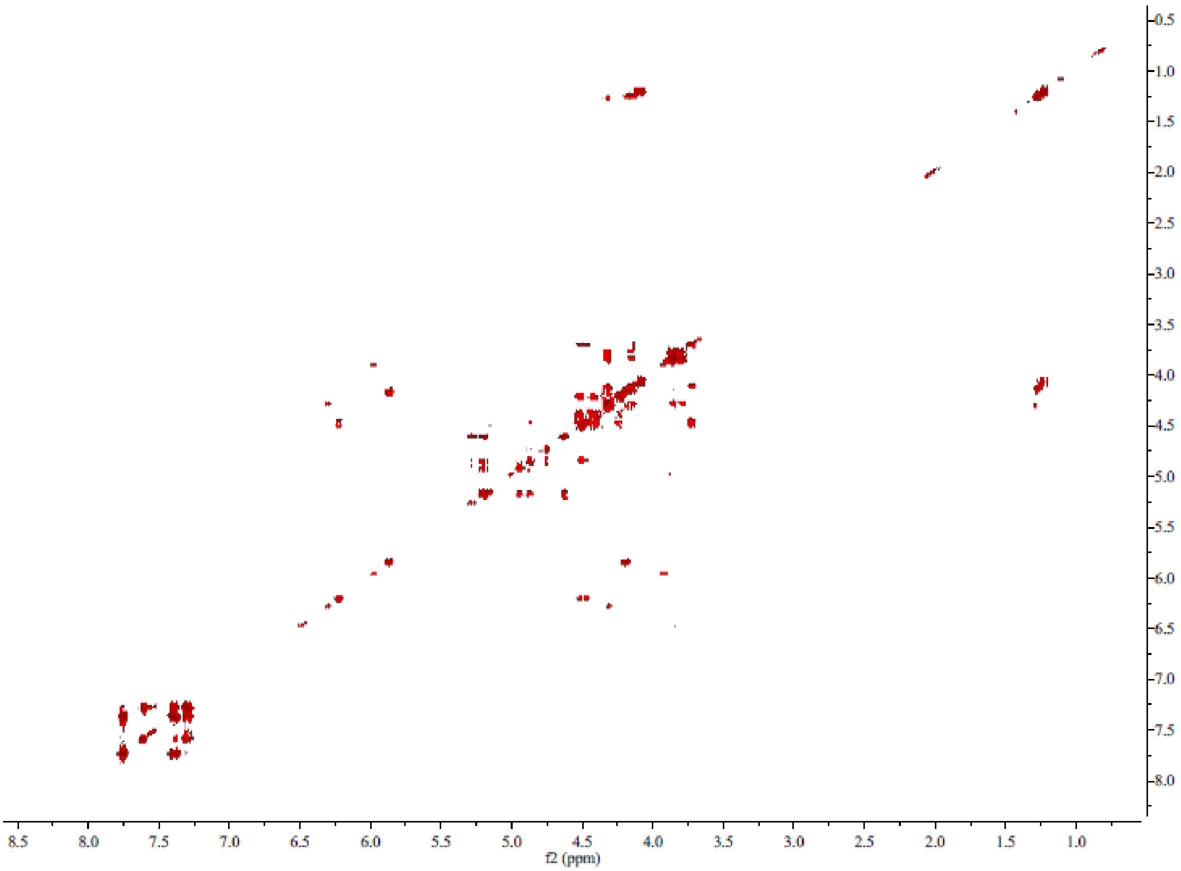

8.5
8.0
7.5
7.0
6.5
6.0
5.5
5.0
4.5
4.0
3.5
3.0
2.5
2.0
1.5
1.0
f2 (ppm)

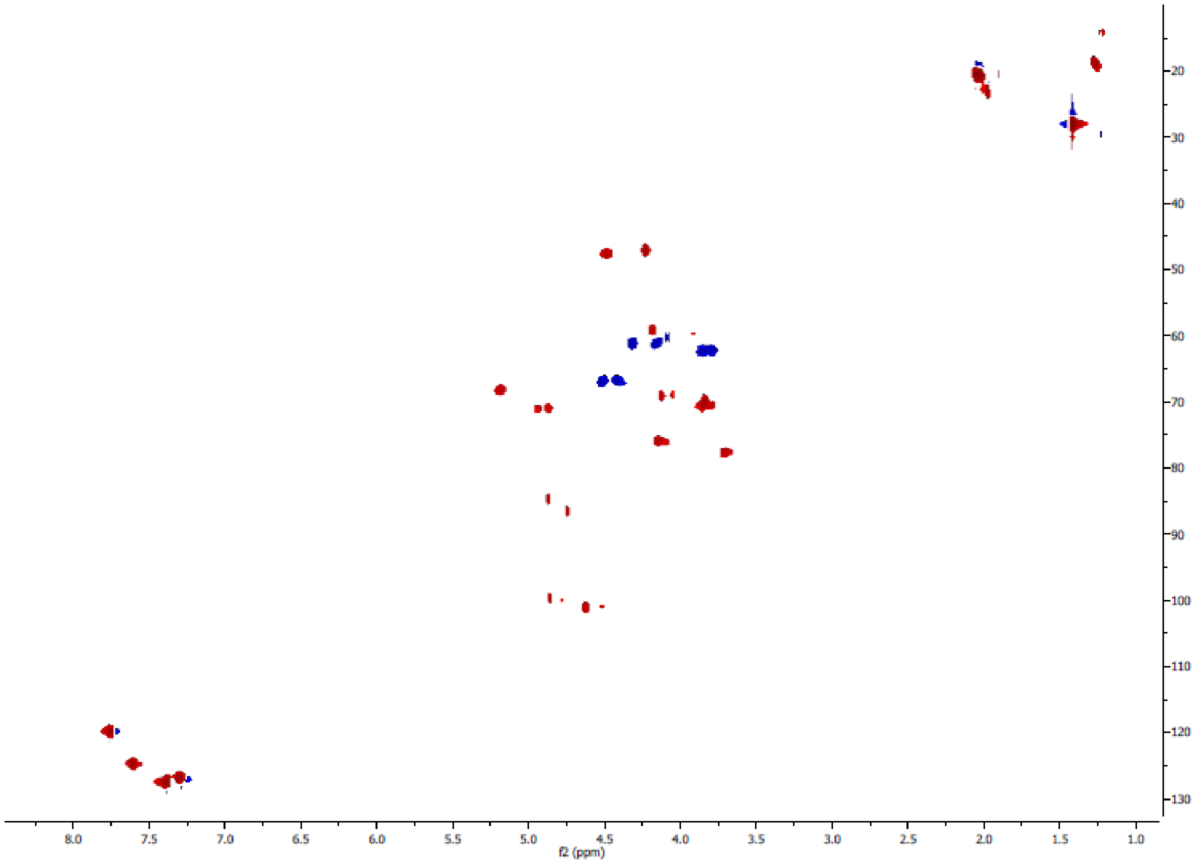

f2 (ppm)

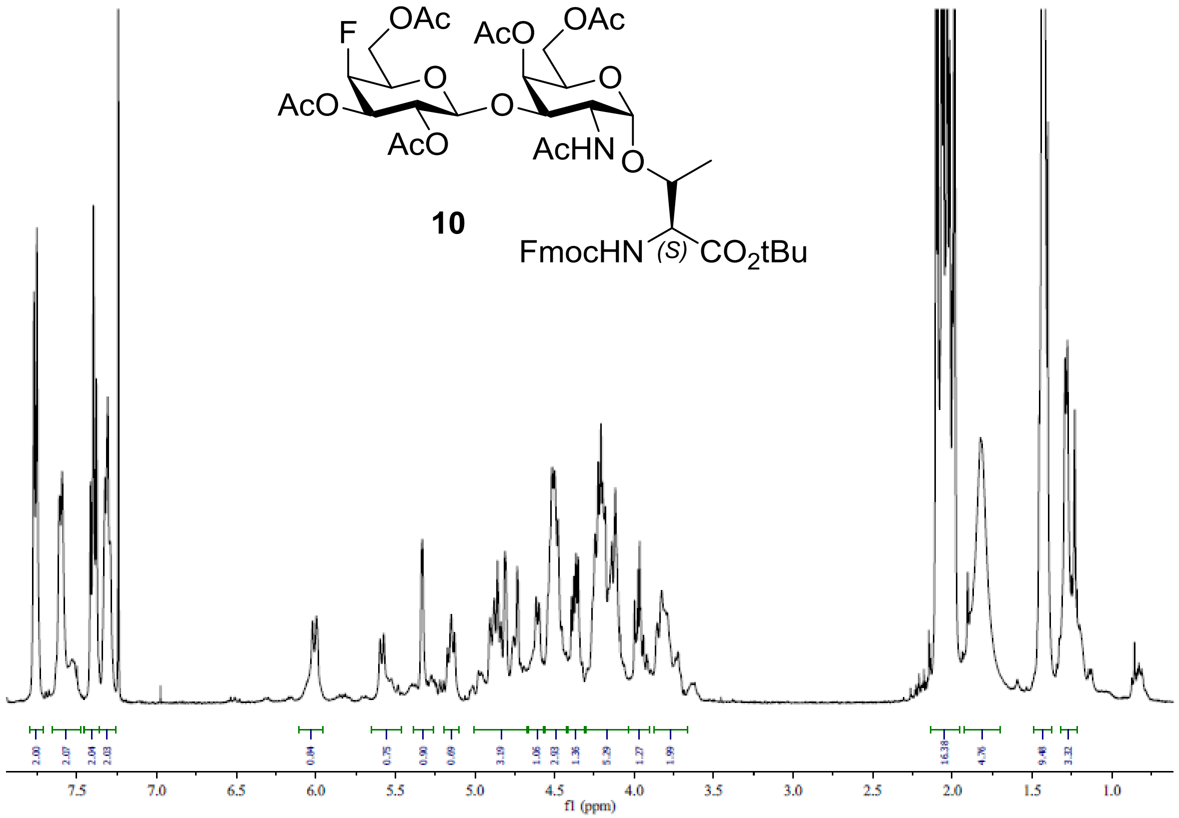
F
OAc
AcO
OAc
AcO
O
AcO
O
AcO
O
AcHN
O
10
FmocHN
(S)
CO_2tBu
f1 (ppm)

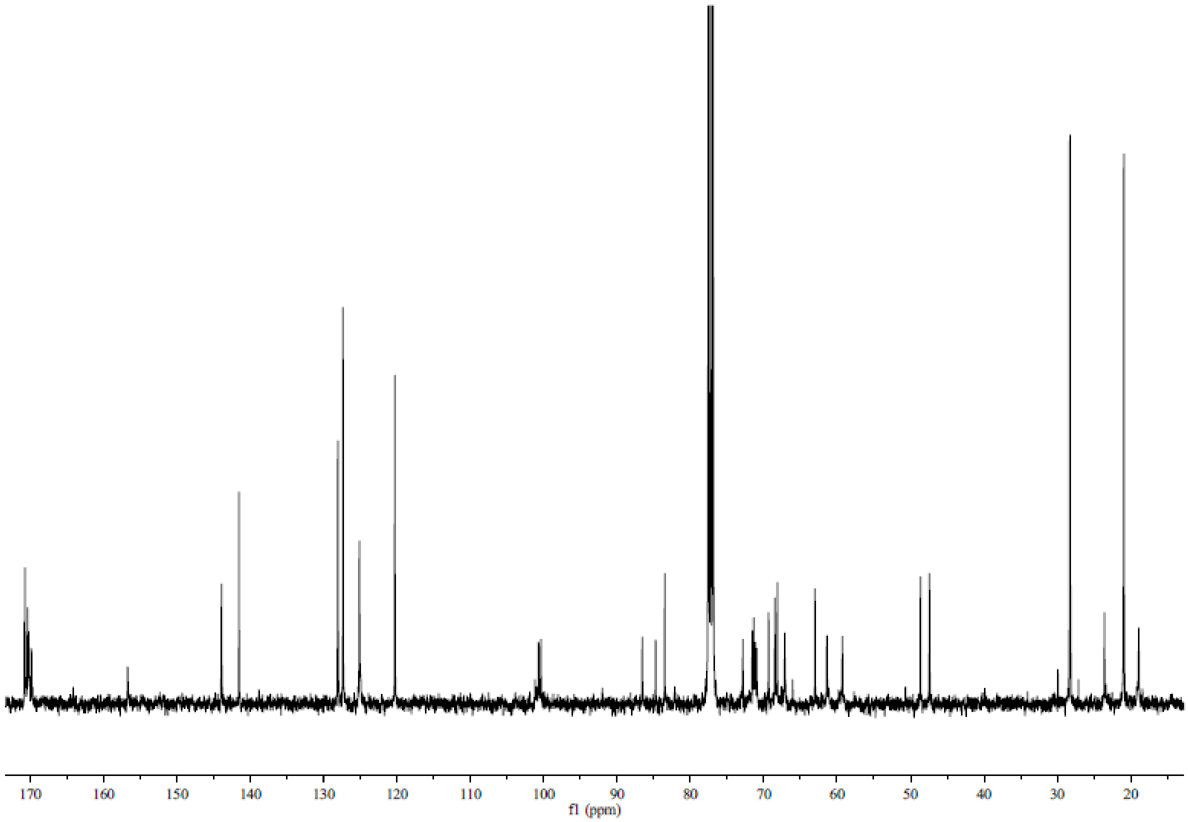
f1 (ppm)

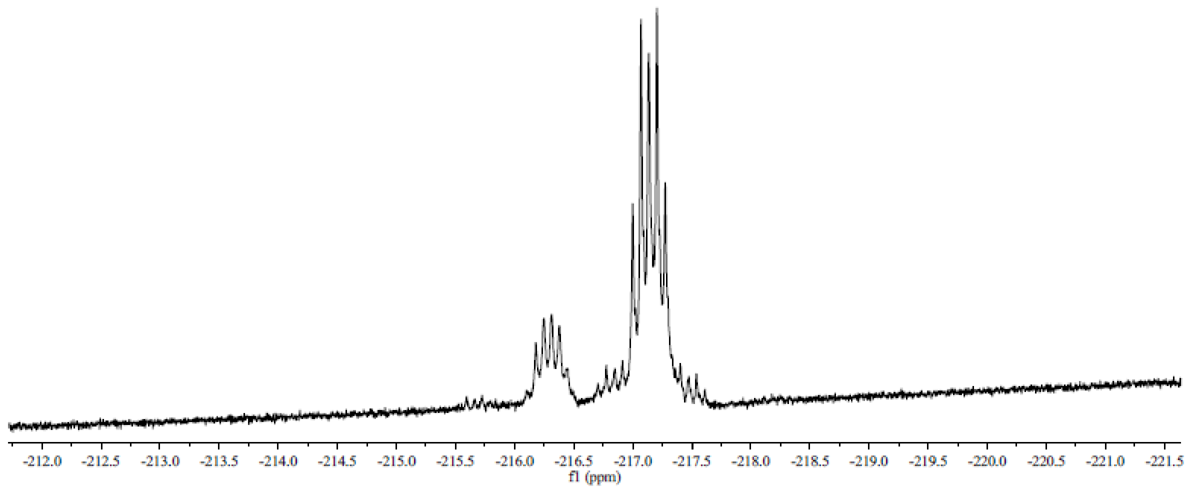
-212.0 -212.5 -213.0 -213.5 -214.0 -214.5 -215.0 -215.5 -216.0 -216.5 -217.0 -217.5 -218.0 -218.5 -219.0 -219.5 -220.0 -220.5 -221.0 -221.5
f1 (ppm)

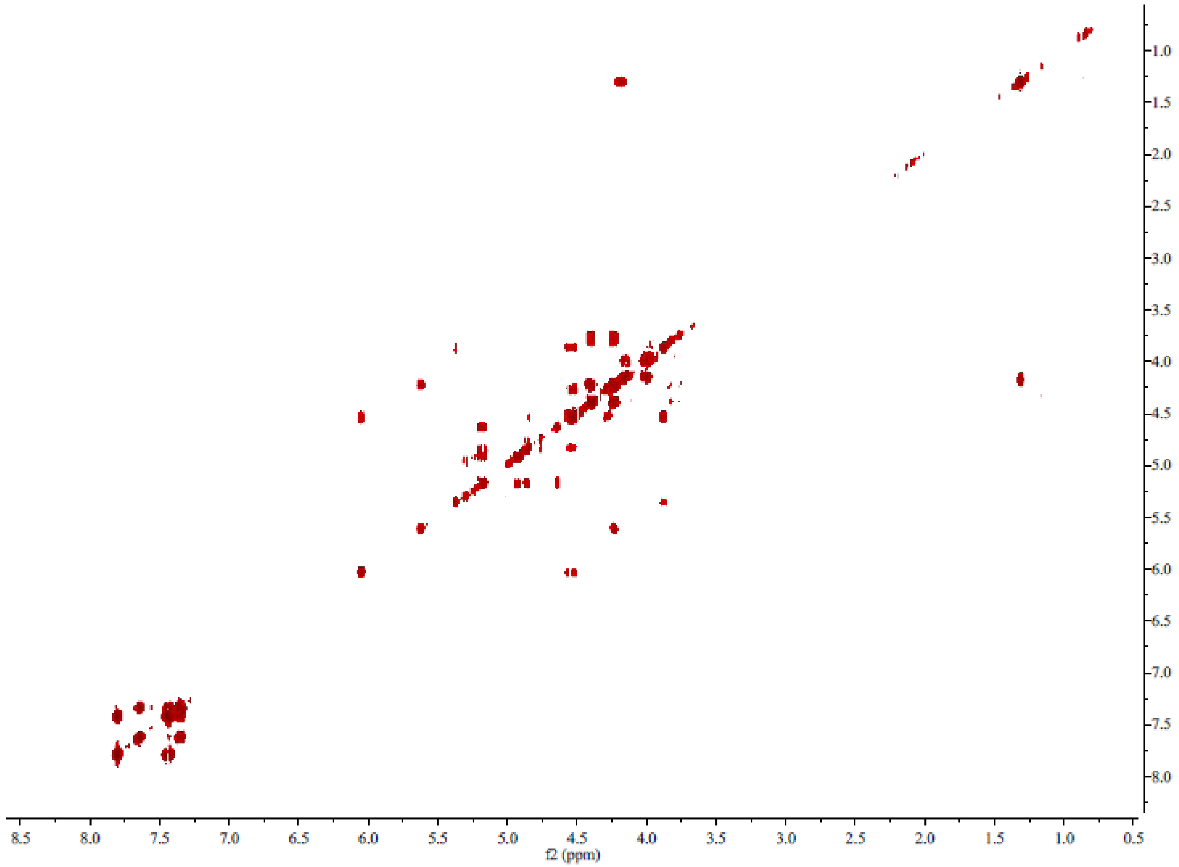
1.0 1.5 2.0 2.5 3.0 3.5 4.0 4.5 5.0 5.5 6.0 6.5 7.0 7.5 8.0
8.5 8.0 7.5 7.0 6.5 6.0 5.5 5.0 4.5 4.0 3.5 3.0 2.5 2.0 1.5 1.0 0.5
f2 (ppm)

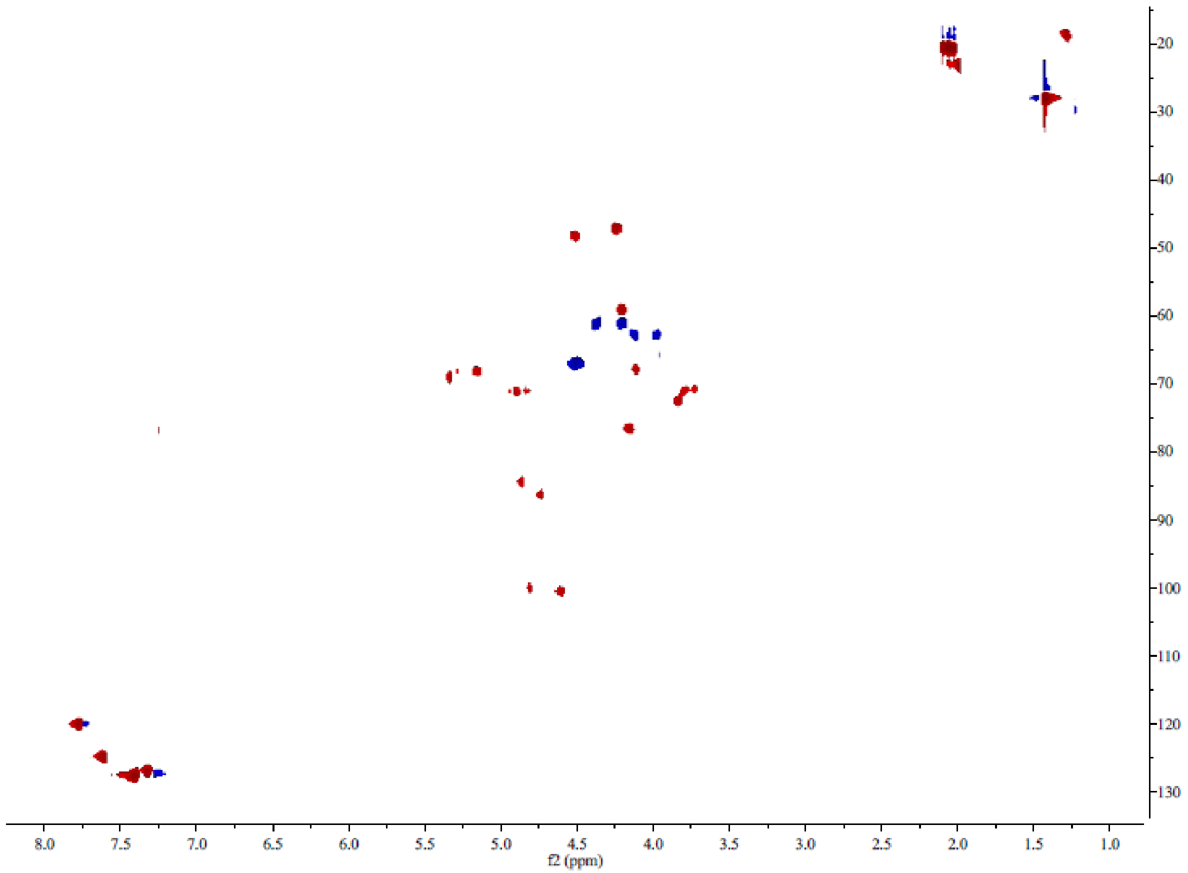

20
30
40
50
60
70
80
90
100
110
120
130
8.0
7.5
7.0
6.5
6.0
5.5
5.0
4.5
4.0
3.5
3.0
2.5
2.0
1.5
1.0
f2 (ppm)

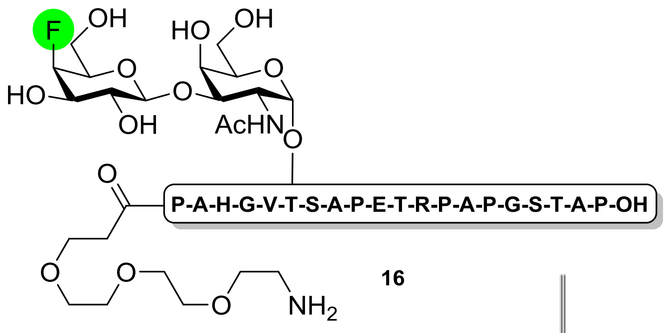
F
OH
HO
OH
O
HO
OH
AcHN
O
P-A-H-G-V-T-S-A-P-E-T-R-P-A-P-G-S-T-A-P-OH
NH2

16

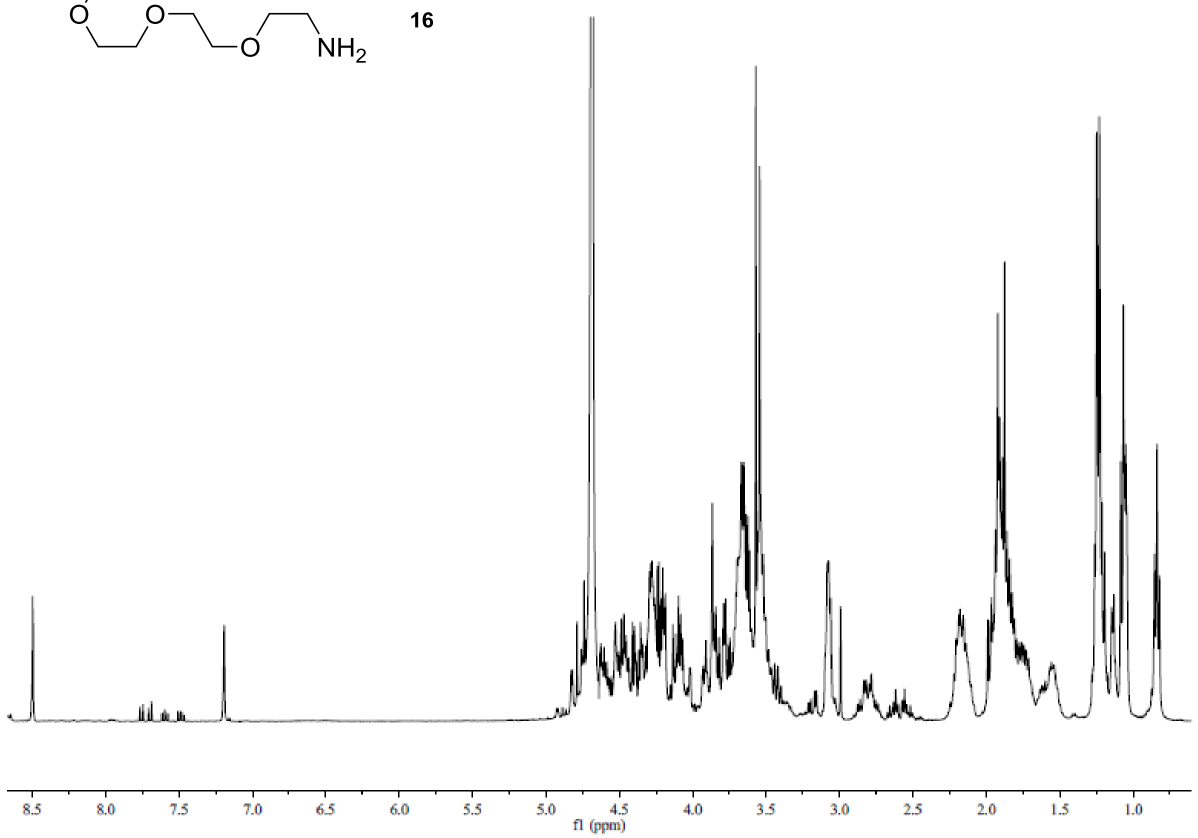
8.5 8.0 7.5 7.0 6.5 6.0 5.5 5.0 4.5 4.0 3.5 3.0 2.5 2.0 1.5 1.0
f1 (ppm)

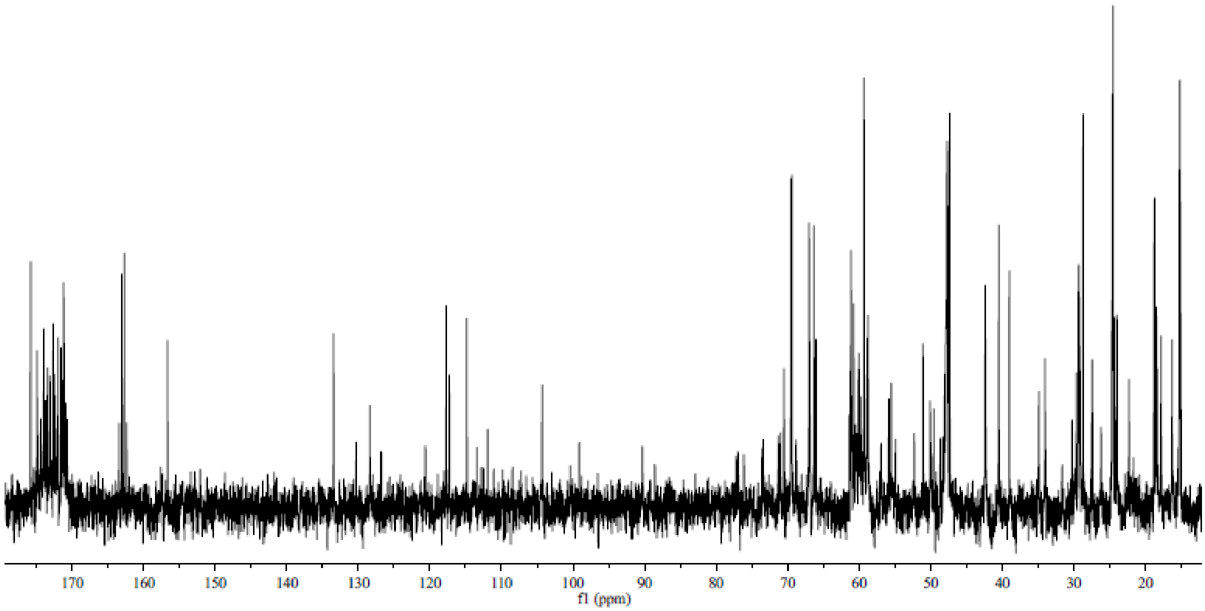
170 160 150 140 130 120 110 100 90 80 70 60 50 40 30 20
f1 (ppm)

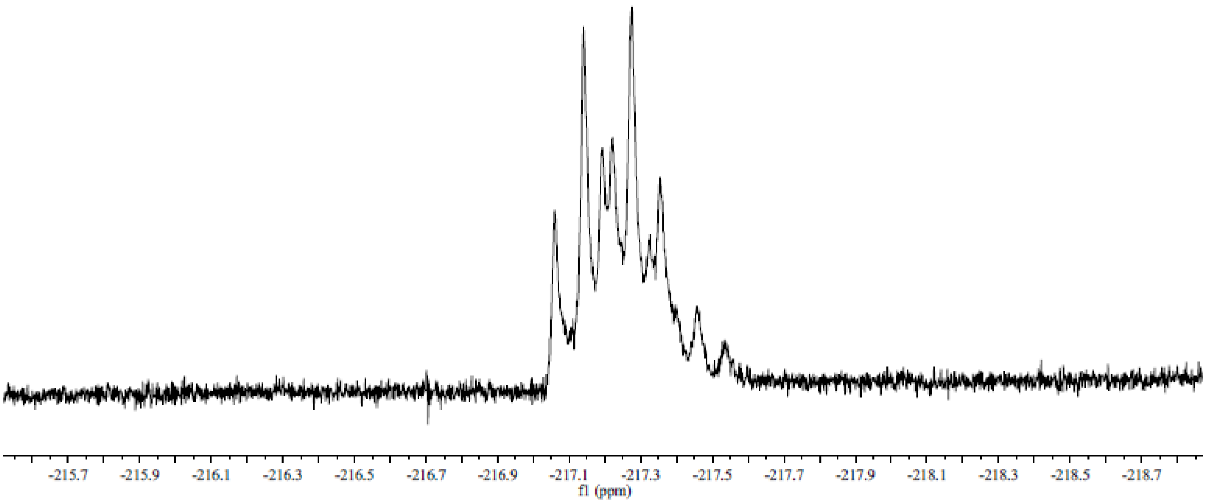
-215.7
-215.9
-216.1
-216.3
-216.5
-216.7
-216.9
-217.1
-217.3
-217.5
-217.7
-217.9
-218.1
-218.3
-218.5
-218.7
f1 (ppm)

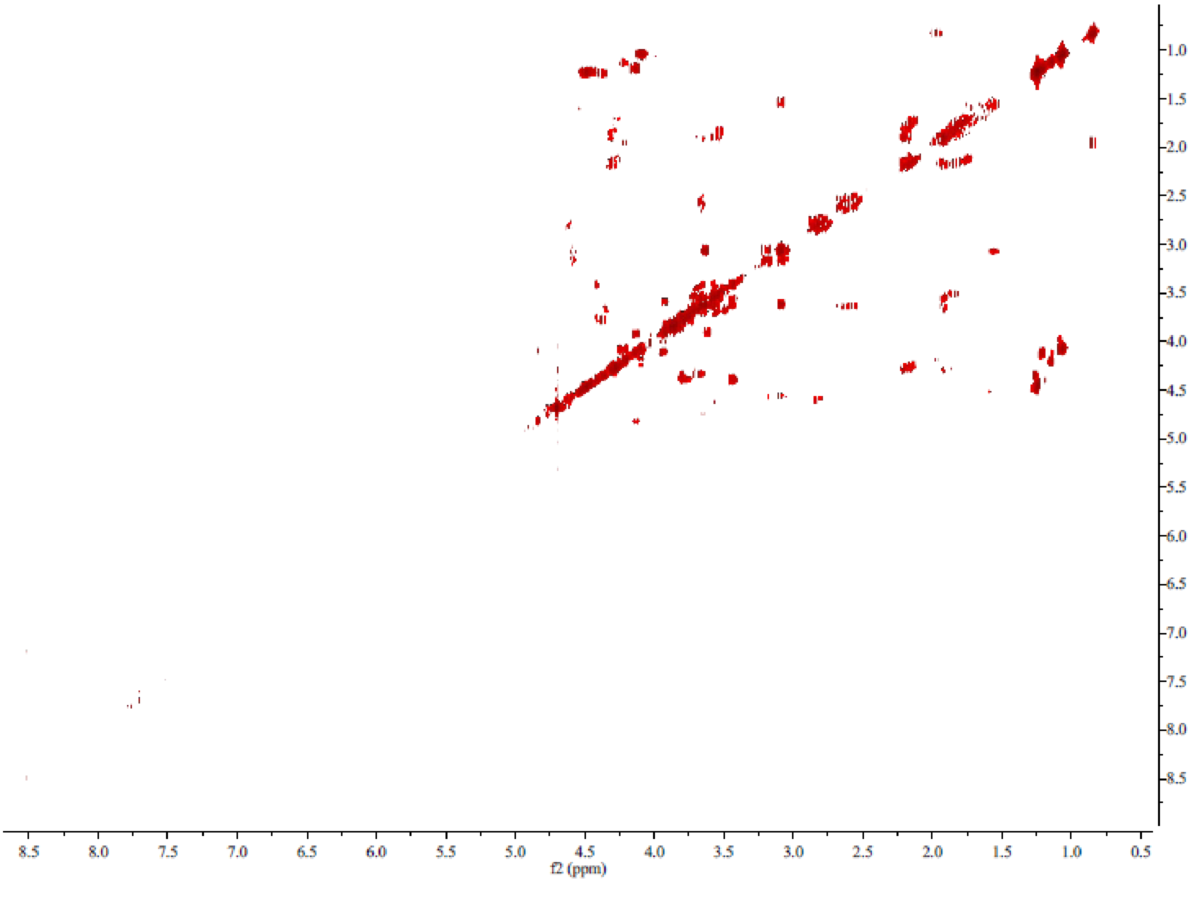
8.5
8.0
7.5
7.0
6.5
6.0
5.5
5.0
4.5
4.0
3.5
3.0
2.5
2.0
1.5
1.0
0.5
f2 (ppm)

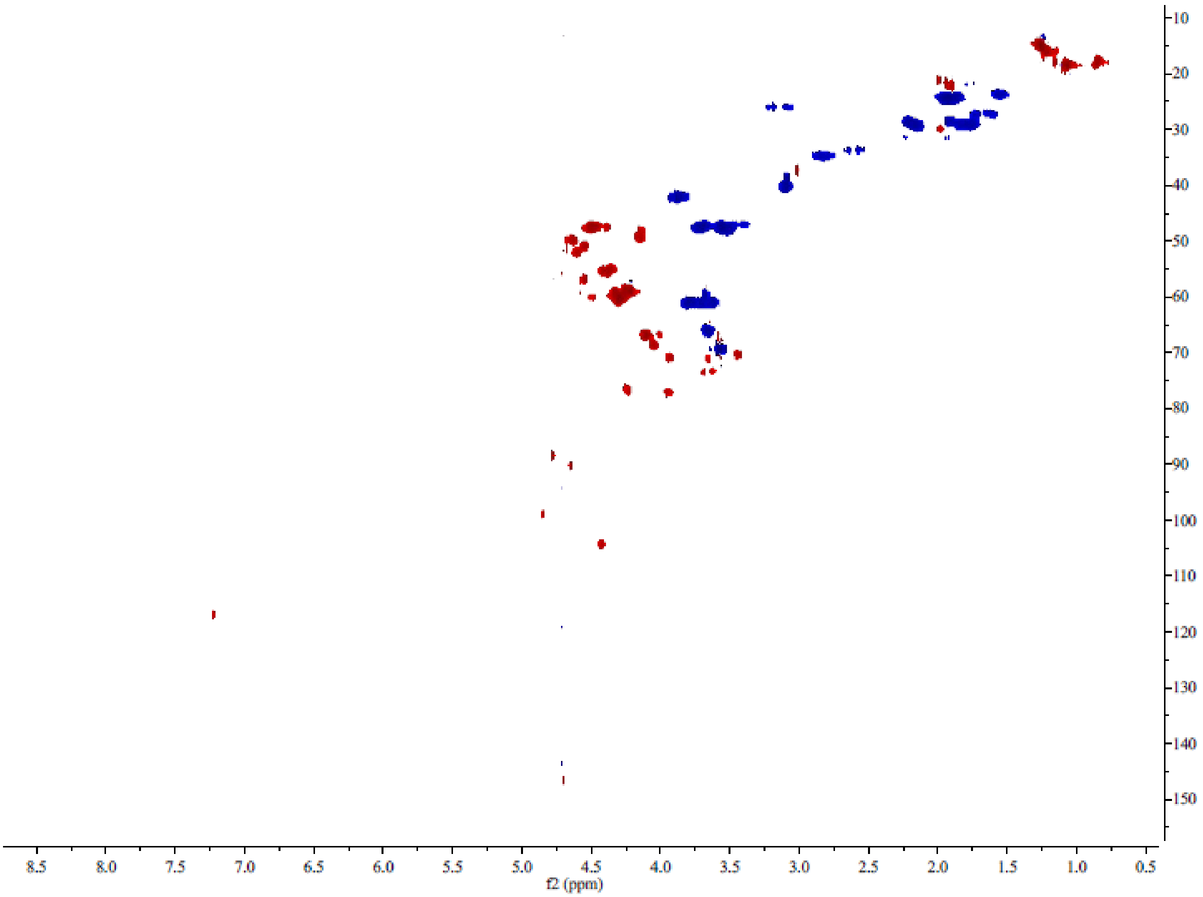

f2 (ppm)

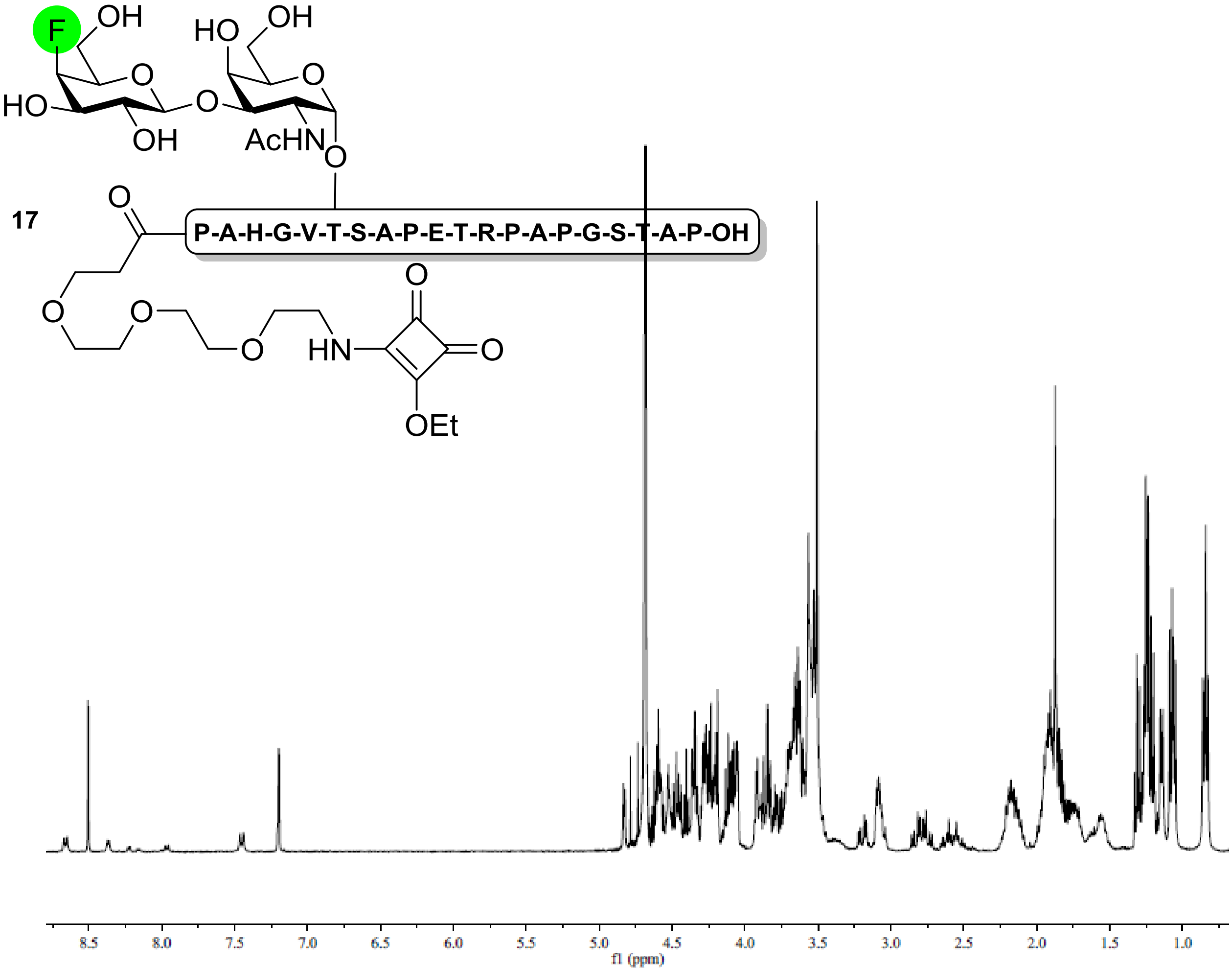
F
OH
HO
OH
O
HO
AcHN
17
P-A-H-G-V-T-S-A-P-E-T-R-P-A-P-G-S-T-A-P-OH
HN
OEt
f1 (ppm)

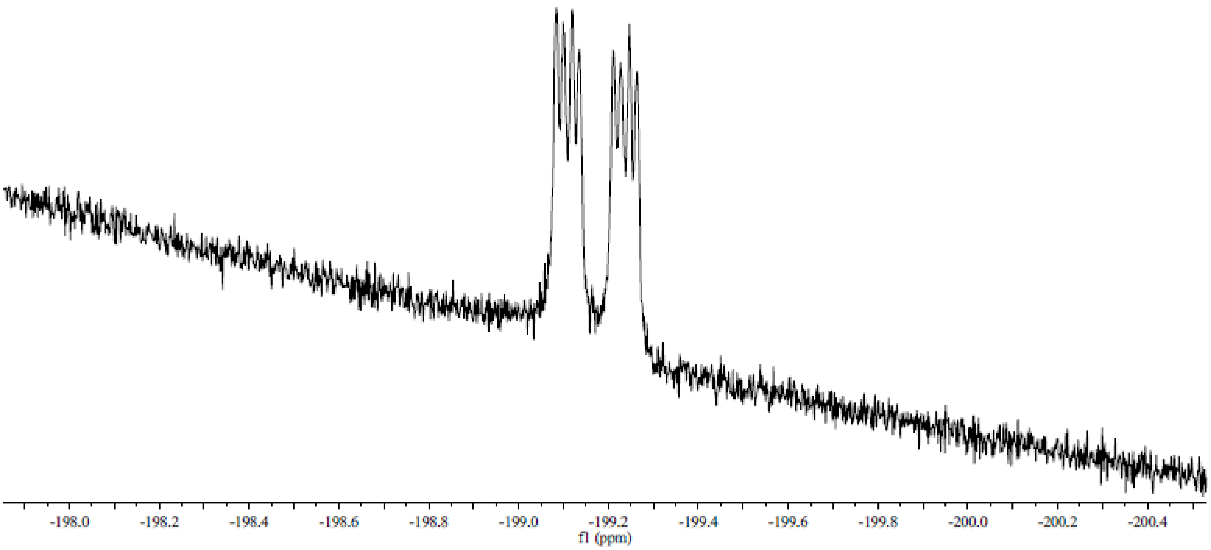
f1 (ppm)

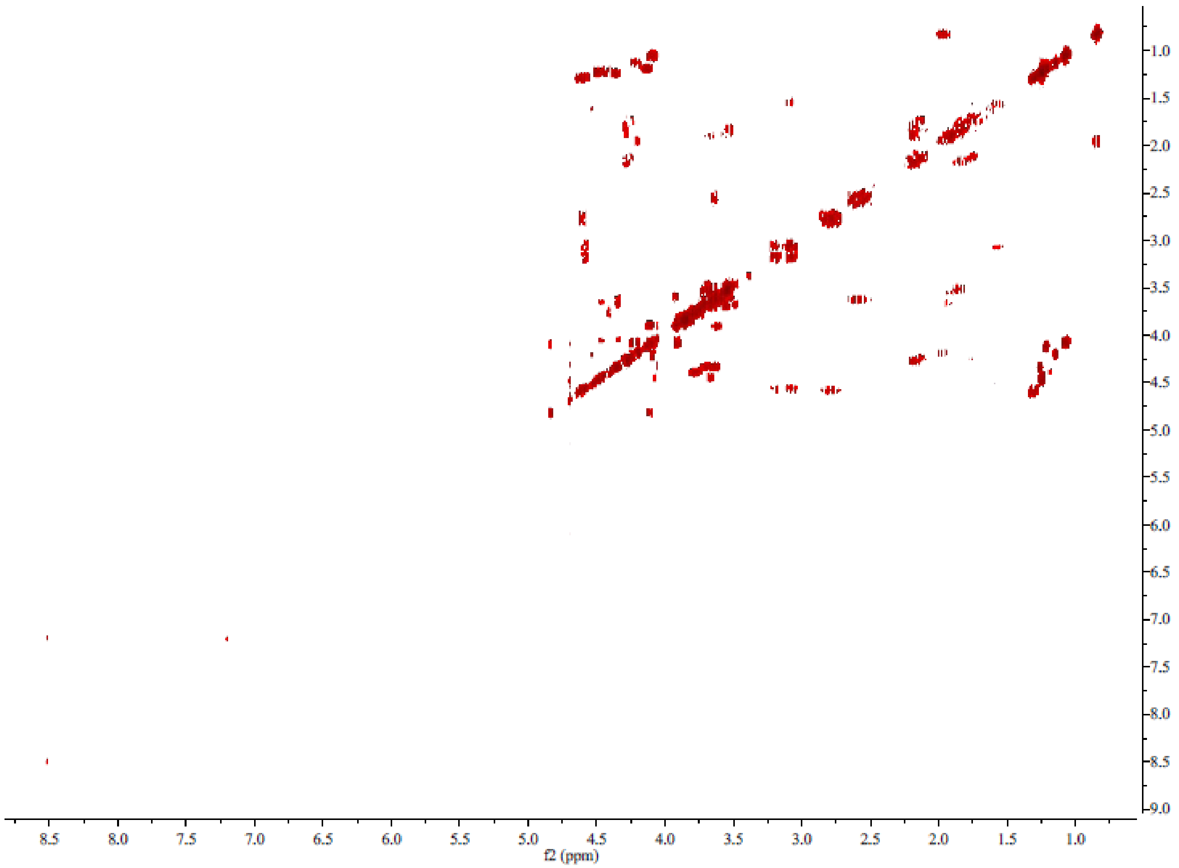
8.5
8.0
7.5
7.0
6.5
6.0
5.5
5.0
4.5
4.0
3.5
3.0
2.5
2.0
1.5
1.0
f2 (ppm)
1.0
1.5
2.0
2.5
3.0
3.5
4.0
4.5
5.0
5.5
6.0
6.5
7.0
7.5
8.0
8.5
9.0

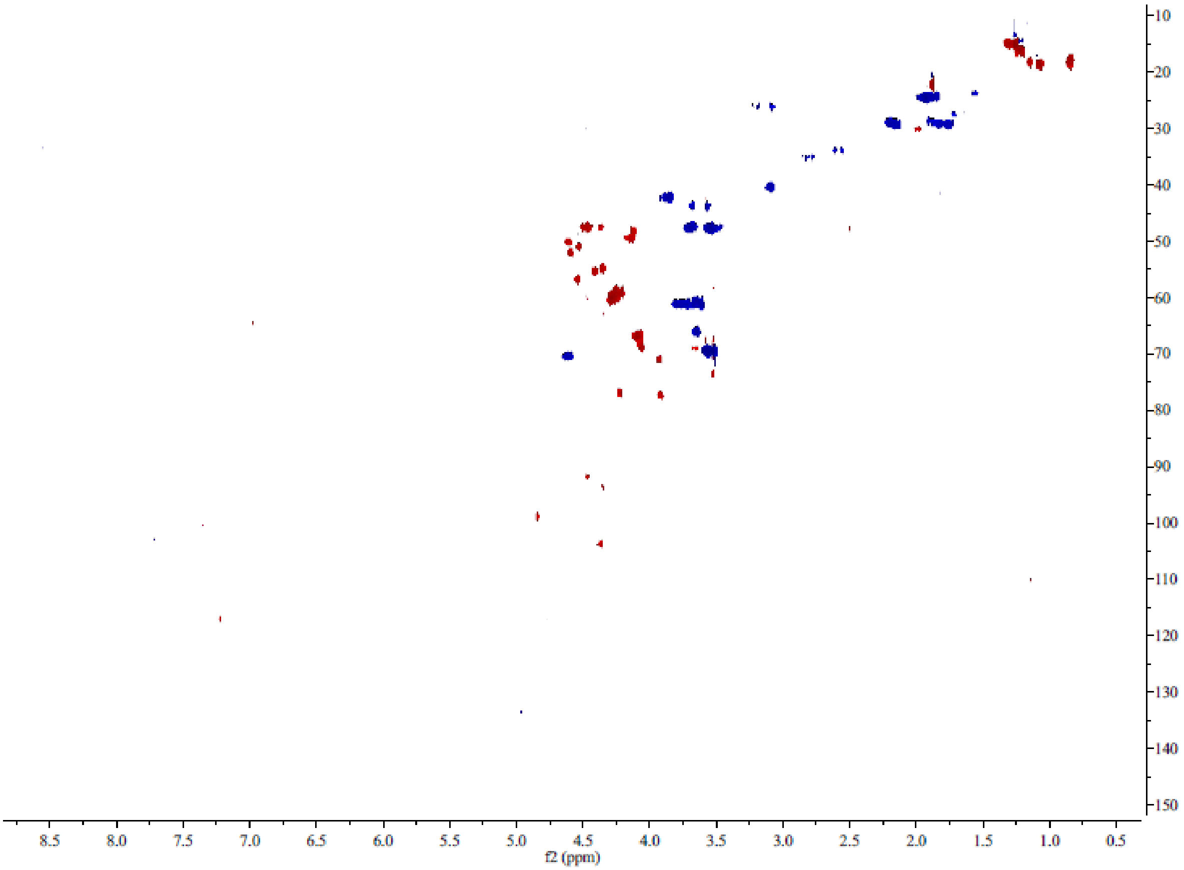
8.5
8.0
7.5
7.0
6.5
6.0
5.5
5.0
4.5
4.0
3.5
3.0
2.5
2.0
1.5
1.0
0.5
f2 (ppm)
10
20
30
40
50
60
70
80
90
100
110
120
130
140
150

6 References

[1] a) F. Zhang, W. Zhang, Y. Zhang, D. P. Curran, G. Liu, *J. Org. Chem.* **2009**, *74*, 2594-2597; b) S.-S. Weng, Y.-D. Lin, C.-T. Chen, *Org. Lett.* **2006**, *8*, 5633-5636.

[2] K.-K. T. Mong, C.-S. Chao, M.-C. Chen, C.-W. Lin, *Synlett* **2009**, 603-606.

[3] U. Ellervik, M. Jacobsson, J. Ohlsson, *Tetrahedron* **2005**, *61*, 2421-2429.

[4] A. Hoffmann-Röder, A. Kaiser, S. Wagner, N. Gaidzik, D. Kowalczyk, U. Westerlind, B. Gerlitzki, E. Schmitt and H. Kunz, *Angew. Chem. Int. Ed.*, 2010, **49**, 8498-8503.

[5] a) Anton Kaiser, *Dissertation*, Johannes Gutenberg-Universität Mainz, **2009**; b) A. Kaiser, N. Gaidzik, T. Becker, C. Menge, K. Groh, H. Cai, Y.-M. Li, B. Gerlitzki, E. Schmitt, H. Kunz, *Angew. Chem. Int. Ed.*, 2010, **49**, 3688-3692.

[6] provided by E. Schmitt, Institut für Immunologie, Universitätsmedizin Mainz, Germany

[7] provided by H. Kunz, Institut für Chemie, Johannes Gutenberg-Universität Mainz, Germany
